# Immunoinformatics design of a structural proteins driven multi-epitope candidate vaccine against different SARS-CoV-2 variants based on fynomer

**DOI:** 10.1038/s41598-024-61025-2

**Published:** 2024-05-04

**Authors:** Javad Sarvmeili, Bahram Baghban Kohnehrouz, Ashraf Gholizadeh, Dariush Shanehbandi, Hamideh Ofoghi

**Affiliations:** 1 Department of Plant Breeding and Biotechnology, University of Tabriz, Tabriz, 51666 Iran; 2 Department of Animal Biology, University of Tabriz, Tabriz, 51666 Iran; 3 Department of Immunology, Tabriz University of Medical Sciences, Tabriz, 51666 Iran; 4 Department of Biotechnology, Iranian Research Organization for Science and Technology, Tehran, 33131 Iran

**Keywords:** Computational biology and bioinformatics, Predictive medicine, Protein analysis, Protein design, Protein structure predictions

## Abstract

The ideal vaccines for combating diseases that may emerge in the future require more than simply inactivating a few pathogenic strains. This study aims to provide a peptide-based multi-epitope vaccine effective against various severe acute respiratory syndrome coronavirus 2 strains. To design the vaccine, a library of peptides from the spike, nucleocapsid, membrane, and envelope structural proteins of various strains was prepared. Then, the final vaccine structure was optimized using the fully protected epitopes and the fynomer scaffold. Using bioinformatics tools, the antigenicity, allergenicity, toxicity, physicochemical properties, population coverage, and secondary and three-dimensional structures of the vaccine candidate were evaluated. The bioinformatic analyses confirmed the high quality of the vaccine. According to further investigations, this structure is similar to native protein and there is a stable and strong interaction between vaccine and receptors. Based on molecular dynamics simulation, structural compactness and stability in binding were also observed. In addition, the immune simulation showed that the vaccine can stimulate immune responses similar to real conditions. Finally, codon optimization and in silico cloning confirmed efficient expression in *Escherichia coli*. In conclusion, the fynomer-based vaccine can be considered as a new style in designing and updating vaccines to protect against coronavirus disease.

## Introduction

Since the end of 2019, humanity has seen the spread of the contagious disease COVID-19 (2019 COronaVIrus Disease) caused by SARS-CoV-2 and its different variants^1^. Most countries have been involved in this disease to some extent regarding health, social, and economic issues. Also, some pharmaceutical and non-pharmaceutical companies have spent significant time and money to combat this disease^2^. The World Health Organization (WHO) has classified this pandemic as an emergency health problem and an ongoing global health crisis due to its capacity to spread rapidly and the multiple alterations of the virus that have resulted in variants of concern (VOCs). Following the issuance of emergency licenses for the production and distribution of vaccines, the organization declared that over 150 countries had joined the Global COVID-19 Immunization Access Initiative (COVAX) to address the issue of rapid and equitable access to vaccines around the world^3–5^. Producing and developing high-efficacy vaccines is the most effective approach to reducing disease burden, controlling its spread, and achieving eradication^6^. However, ongoing research is also being conducted on various aspects of the virus and its associated disease, encompassing the understanding of disease characteristics, mutations, variants, prevention strategies, treatment methods, vaccine efficacy, immune evasion, and the roles of neutralizing and binding antibodies.

The large enveloped virus SARS-CoV-2 with a single-stranded RNA genome prone to rapid mutation with a positive sense of approximately 29.9 kb in length, is a member of the beta-coronavirus belonging to the *Coronaviridae* that can cause infection and pneumonia in birds, mammals, and humans^7,8^. This virus is composed of four structural proteins: spike (S), nucleocapsid (N), membrane (M), and envelope (E), as well as several non-structural proteins (nsp)^9^. The availability of genomic and proteomic sequences has made it possible to obtain more genetic information, identify genes, and develop vaccines for this disease. Researchers propose using vaccines as a consistent and effective approach that could provide long-term protection against all virulent strains^10^. This study aims to develop an epitope-based vaccine that activates innate and acquired immunity. Antigenicity is attributed to the viral structural proteins, especially the S protein^11^; therefore, many epitopes have been identified for this protein's T and B-cell epitopes. The importance of the S protein in infection and pathogenesis makes it an ideal target and focus for SARS-CoV-2 vaccine design and development, as it is the leading viral antigen neutralized by antibodies during infection^7^. Epitopes located on structural proteins are part of the antigenic determinants that can initiate cellular immune responses and are recognized by specific receptors on the surface of T or B-cells^12,13^. Accordingly, identifying and studying epitopes is essential for developing diagnostic methods and epitope-based vaccines^14^.

The presence of advanced in silico immunoinformatics tools by predicting and screening candidate epitopes and designing effective vaccines against diseases reduces the burden of experimental immunity on the model organism^15^. These tools offer a precise, rapid, cost-effective approach to developing potential multi-epitope vaccines against infectious diseases^16^. On the other hand, instead of using complete proteins and attenuated pathogens, which can cause sensitization and other immune reactions such as local redness, pain swelling, or increased body temperature after vaccination, designing a multi-epitope vaccine will be more reliable^16^. Furthermore, this approach has several advantages, including engineering epitopes for greater potency, allowing immune responses to conserved epitopes, and improving immune profiles^17^, which apply to different SARS-CoV-2 strains.

In this study, epitopes of the spike, nucleocapsid, envelope, and membrane structural proteins of 32 different variants of the SARS-CoV-2 virus, which are antigenic, have been used as targets for T and B-cells in the design of potential vaccine structure. Furthermore, to enhance therapeutic efficacy and stabilize the multi-epitope state of the structure, the example of different types of antibody mimetics known as fynomer has also been proposed for the first time. This led to the combination of the chosen epitopes around the fynomer scaffold. Fynomers are small globular binding proteins with a molecular weight of 7 kDa, composed of amino acids 83 to 145 of the Src homology 3 (SH3) domain of the human tyrosine-protein kinase Fyn^18^. Their structure contains two antiparallel β-sheets and two flexible loops called RT and Src loops, essential in interactions with other proteins^19^. Advantages associated with fynomer scaffolds include fast and easy purification, no tendency to form aggregates and being monomeric, high stability with a melting temperature (Tm) ~ 70 °C, lac of cysteine residue that allows combining with other proteins without misfolding problems, expressing in *Escherichia coli* at a high level in soluble form, having a human origin and the existence of a completely conserved amino acid sequence between humans and mice, which indicates that it is non-immunogenic in mammals^20^. Based on these features, it can be claimed that fynomer not only has the potential to be engineered to produce non-hemoglobin proteins with specific binding domains and high affinity to target pathogens specifically but can also be incorporated into the structure of vaccines to increase their stability and effectiveness.

**Figure 1:**
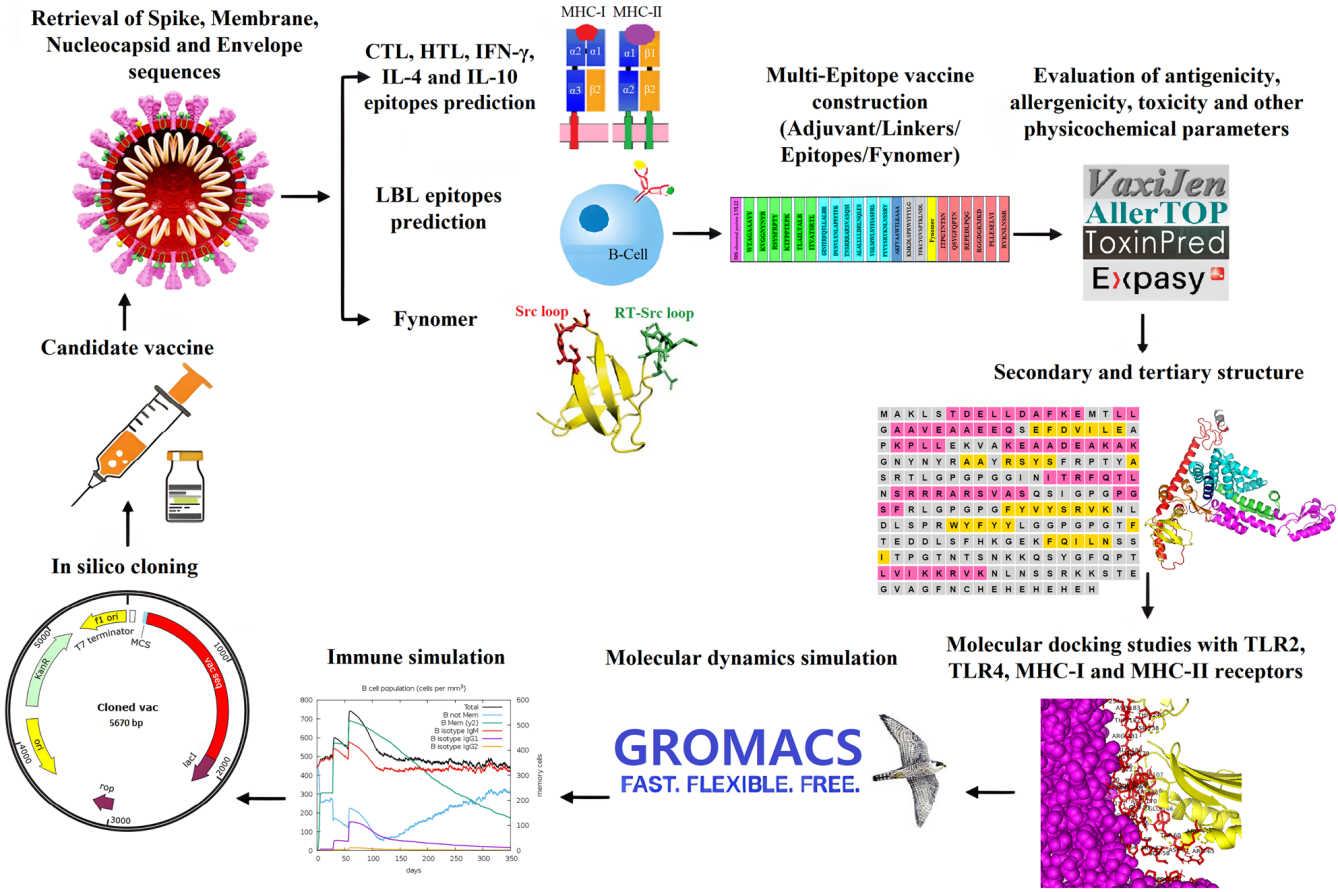
Schematic workflow of in silico multi-epitope vaccine design process.

In order to understand safety and effectiveness, the physicochemical, immunogenic, and dynamic characteristics of the designed vaccine were examined after obtaining the final structure. Finally, in silico cloning and immune simulations were also carried out (Fig. 1). Vaccination is the most effective way to prevent infectious diseases, which generally calls for the use of thorough yet precise methods. As it is crucial for the design of multi-epitope vaccines to monitor genetic and antigenic changes in the viral population globally to deal with emerging variants, this study, with consideration for changes in protein structures, suggests a candidate vaccine with the capacity to elicit humoral and cellular immune responses to deal with broad variants.

## Results

### Prediction of antigenicity and alignment of protein sequences

**Figure 2:**
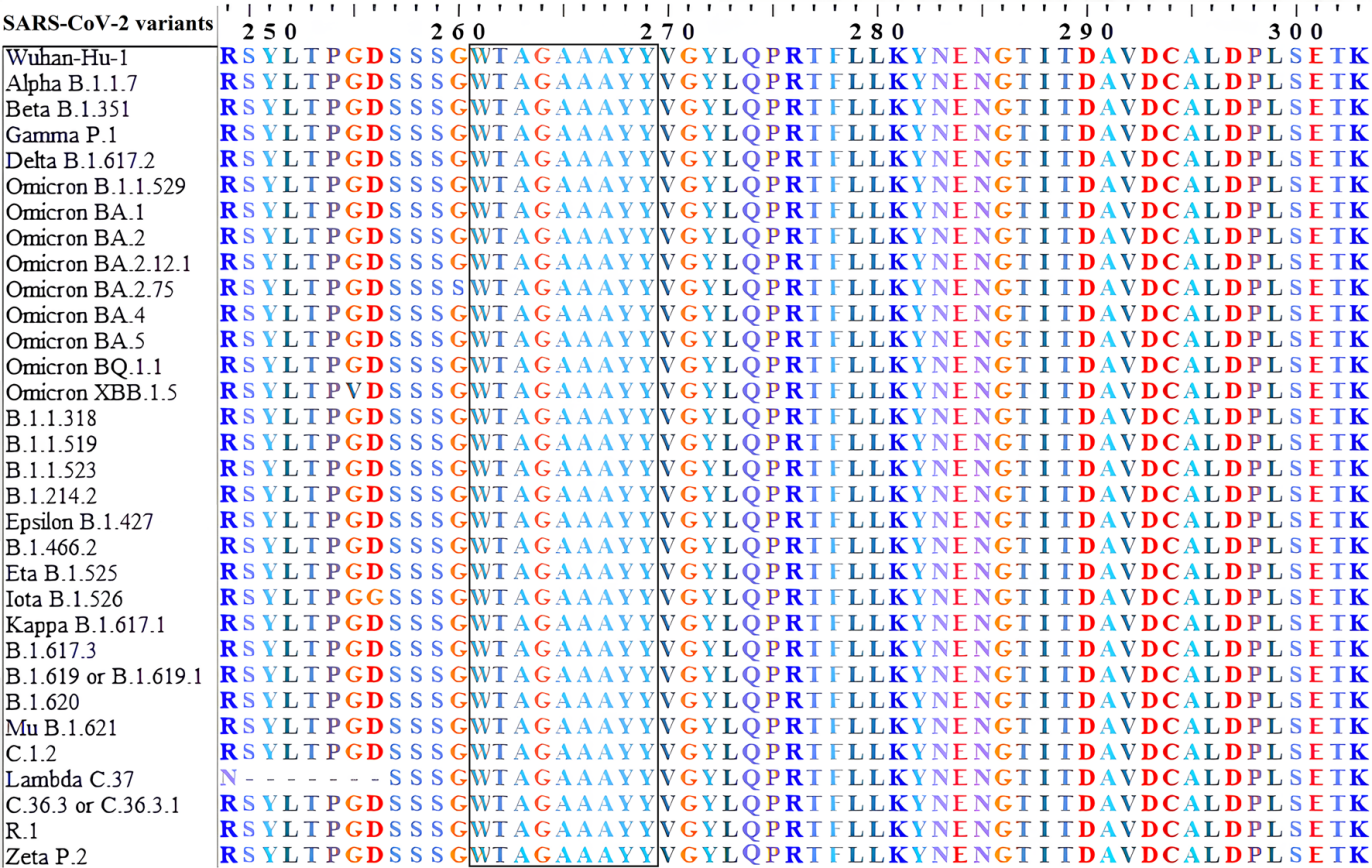
Multiple sequence alignment (MSA) for structural proteins of 32 SARS-CoV-2 variants using ClustalW. One predicted epitope was highlighted with a black box line in the conserved region.

According to the antigenicity screening of the amino acid sequences of four structural proteins (Spike, Nucleocapsid, Membrane, and Envelope) from 32 SARS-CoV-2 strains (Supplementary Tables S1–S3), the S protein had the most excellent antigenic value, followed by proteins E, M, and N, in that order. It was also determined that these proteins could serve as desirable antigens. Hence, these proteins were selected to predict T and B-cell epitopes and vaccine development. After examining the results of sequence alignment, protective and non-protective regions of these structural proteins in the desired strains were identified by the identity of the amino acid sequences (Fig. 2). In contrast to the other three proteins, spike protein has effective mutations, particularly in the RBD (Receptor binding domain) and NTD (N-terminal domain) regions, which are effective in the severity of pathogenicity, antibody escape, and disease transmission^21^. To enhance the efficacy of the designed vaccine against wild-type and mutant SARS-CoV-2, it is suggested that the vaccine design strategy be based on the identification and selection of epitopes that are recognized by both predictive tools as stimulating T and B lymphocytes in the immune system and are among the non-mutated and fully conserved parts of the virus. This approach ensures the vaccine targets parts of the virus that are less likely to undergo mutations and remain effective against different strains. However, it is essential to note that the immune response can be complex and may involve factors beyond the predicted conservancy of epitopes. Therefore, it is necessary to confirm the effectiveness of antigenically protected epitopes (ability to stimulate an immune response) and provide complete protection through experimental evidence^22^.

### Prediction of T-cell epitopes: CTL and HTL

Pathogen or vaccine peptides in the cytosol undergo fragmentation by the proteasome, which unfolds the protein and cleaves it into peptide fragments. They are transported to the endoplasmic reticulum by the protein transporter TAP. Only peptides of the right size stably bind to the MHC I groove^23^. The NetCTL 1.2 server assesses these features, evaluating the proteasomal cleavage, TAP transport efficiency, and MHC class I binding. Higher predicted values for these indicators increase the likelihood of peptide positioning in the MHC-I groove, facilitating CTL recognition^24^. NetCTL1.2 predicted 440 epitopes for S protein, 104 for N, 126 for M, and 51 for E protein. These epitopes showed binding solid affinity to multiple alleles within the 12 MHC class I supertypes. All these epitopes were included among the results obtained from IEDB as selected items by considering all MHC alleles available in this server for these structural proteins. On the other hand, the NetMHC II pan 3.2 server, which was used to predict HTL epitopes, predicted 1605 epitopes for S, 359 for N, 169 for M, and 54 for E. After checking, it was found that IEDB also suggested these same epitopes as top epitopes for the alleles that are in this server, which is a kind of confirmation of the predicted epitopes through both servers. Among all these predicted cytotoxic T lymphocytes (CTLs) and helper T lymphocytes (HTLs) epitopes, the best ones were selected based on antigenicity, allergenicity, toxicity, and most importantly, 100% conservation in all SARS-CoV-2 strains. According to these criteria, 39 CTL epitopes of S protein, 14 of N, 20 of M, and 13 of E were selected. Additionally, 52 HTL epitopes of S, 28 of N, 19 of M, and 11 of E were screened (see Supplementary Tables S4–S11). Through the use of IFNepitopes, IL4pred, and IL10pred servers, an investigation was conducted to determine the inducibility of interferon-γ (IFN-γ), interleukin-4 (IL-4), and Interleukin-10 (IL-10). The results revealed 4 epitopes in S, 1 in N, and 1 in M, all meeting the necessary criteria. It should be noted that no epitopes meeting these criteria were identified in E. In this study, to achieve the highest immunogenicity and reduce epitope overload in the final vaccine construct, only one epitope from the S and N proteins with the highest scores for the investigated criteria was selected (Supplementary Table S12). The T-cell epitopes used in the final vaccine formulation are listed in Tables 1, 2 and 3. Supplementary Fig. S1 shows the conformational positions of these epitopes on each structural protein derived from it.

**Table 1:** The selected CTL epitopes for the final vaccine construction are provided by the NetCTL and IEDB servers.

Proteins/epitopes (position)	Supertypes/HLA class I alleles	Immunogenicity score	Antigenicity/allergenicity	Toxicity	Epitope conservation (%)	Homology to human proteins
Spike/WTAGAAAY (258–266)	A1, A26, B58, B62/ HLA-A*68:23, HLA-B*15:17, HLA-C*03:03, HLA-A*26:02, HLA-A*29:02, HLA-A*32:15, HLA-B*40:13, HLA-C*12:03, HLA-A*30:02, HLA-B*15:02, HLA-A*26:01, HLA-A*32:07, HLA-B*35:01,HLA-B*27:20, HLA-A*68:01, HLA-C*14:02	0.1525	0.6306 (antigen)/non-allergen	Non-toxin	100%	Non-homolog
Spike/KVGGNYNYR (442–450)	A3/ HLA-A*68:23, HLA-A*31:01, HLA-A*32:07, HLA-A*02:50, HLA-C*12:03, HLA-B*27:20, HLA-B*40:13, HLA-A*32:15, HLA-C*03:03	0.0277	1.5212 (antigen)/non-allergen	Non-toxin	100	Non-homolog
Spike/RSYSFRPTY (490–498)	A1, A3, B27, B58, B62/ HLA-B*15:17, HLA-B*15:03, HLA-A*32:07, HLA-C*12:03, HLA-C*03:03, HLA-A*68:23, HLA-B*27:20, HLA-B*40:13, HLA-A*30:02, HLA-A*32:01, HLA-B*58:01, HLA-A*30:01, HLA-B*15:01, HLA-A*02:17, HLA-C*07:02	0.0083	0.9553 (antigen)/non-allergen	Non-toxin	100	Non-homolog
Nucleocapsid/KTFPPTEPK (361–369)	A3/ HLA-A*11:01, HLA-A*68:23, HLA-A*30:01, HLA-B*27:20, HLA-C*12:03, HLA-B*40:13, HLA-A*32:07, HLA-C*14:02, HLA-A*03:01, HLA-A*31:01, HLA-A*68:01, HLA-C*03:03, HLA-A*32:01	0.1306	0.9553 (antigen)/non-allergen	Non-toxin	100%	Non-homolog
Membrane/ITVATSRTL (168–176)	B62/ HLA-C*03:03, HLA-A*68:23, HLA-B*15:17, HLA-A*32:07, HLA-A*02:50, HLA-C*15:02, HLA-B*15:02, HLA-C*12:03, HLA-B*27:20, HLA-A*02:17, HLA-C*14:02, HLA-B*40:13, HLA-A*02:11	0.1306	0.7571 (antigen)/non-allergen	Non-toxin	100%	Non-homolog
Envelope/TLAILTALR (30–38)	A3/ HLA-B*27:20, HLA-A*68:01, HLA-A*32:07, HLA-A*02:50, HLA-A*68:23, HLA-A*31:01, HLA-C*12:03, HLA-A*32:15, HLA-C*03:03, HLA-A*33:01, HLA-B*40:13	0.1989	0.7223 (antigen)/non-allergen	Non-toxin	100%	Non-homolog

**Table 2:** The selected HTL epitopes for the final vaccine construction are provided by the NetMHC II pan 3.2 and IEDB server.

Proteins/epitopes (position)	HLA class II alleles	Antigenicity/allergenicity	Toxicity	Epitope conservation (%)	Homology to human proteins
Spike/GINITRFQTLLALHR (232–246)	HLA-DRB5*01:01, HLA-DRB1*04:04, HLA-DRB1*01:01, HLA-DRB1*11:01, HLA-DRB1*04:05, HLA-DRB1*04:01, HLA-DRB1*15:01, HLA-DRB4*01:01, HLA-DRB1*07:01, HLA-DRB1*09:01, HLA-DPA1*01:03/DPB1*02:01, HLA-DPA1*03:01/DPB1*04:02, HLA-DPA1*02:01/DPB1*01:01, HLA-DPA1*01/DPB1*04:01	0.5582 (antigen)/non-allergen	Non-toxin	100%	Non-homolog
Spike/TNSRRRARSVASQSI (676–690)	HLA-DRB1*07:01, HLA-DRB1*01:01	0.7267 (antigen)/non-allergen	Non-toxin	100%	Non-homolog
Spike/DYSVLYNLAPFFTFK (361–375)	HLA-DRB1*01:01, HLA-DRB1*04:04, HLA-DRB1*08:03, HLA-DRB1*12:01, HLA-DRB1*13:02, HLA-DRB3*02:02, HLA-DRB3*01:01, HLA-DPA1*01:03/DPB1*04:01, HLA-DPA1*01:03/DPB1*04:02, HLA-DPA1*01,03/DPB1*23:01, HLA-DQA1*01:02/DQB1*06:04, HLA-DPA1*01:03/DPB1*02:01	0.7838 (antigen)/non-allergen	Non-toxin	100%	Non-homolog
Nucleocapsid/ALALLLLDRLNQLES (218–232)	HLA-DRB4*01:01, HLA-DRB1*03:01, HLA-DRB1*11:01, HLA-DRB1*04:04, HLA-DRB1*01:01, HLA-DPA1*03:01/DPB1*04:02, HLA-DPA1*01:03/DPB1*02:01, HLA-DPA1*02:01/DPB1*01:01	0.5057 (antigen)/non-allergen	Non-toxin	100%	Non-homolog
Membrane/VGLMWLSYFIASFRL (88–102)	HLA-DRB5*01:01, HLA-DRB1*04:04, HLA-DRB1*01:01, HLA-DRB1*15:01, HLA-DRB1*04:05, HLA-DRB1*07:01, HLA-DRB1*04:01, HLA-DPA1*01:03/DPB1*02:01, HLA-DPA1*02:01/DPB1*01:01, HLA-DPA1*01/DPB1*04:01	0.6658 (antigen)/non-allergen	Non-toxin	100%	Non-homolog
Envelope/FYVYSRVKNLNSSRV (56–70)	HLA-DRB1*01:01, HLA-DRB1*04:04, HLA-DRB1*07:01, HLA-DRB1*11:01, HLA-DRB1*04:01, HLA-DRB1*04:05, HLA-DRB1*09:01, HLA-DRB1*13:02, HLA-DRB1*15:01	0.6103 (antigen)/non-allergen	Non-toxin	100%	Non-homolog

**Table 3:** The selected IFN-γ, IL-4, and IL-10 epitopes in the final vaccine construct, predicted by IFNepitopes, IL4pred and IL10pred servers.

Proteins/epitopes (position)	IFN-γ‌	IL-4	IL-10	Antigenicity/allergenicity	Toxicity	Epitope conservation (%)	Homology to human proteins
Spike/TFKCYGVSPTKLNDL (25–39)	Positive	Inducer	Inducer	1.4626 (antigen)/non-allergen	Non-toxin	100	Non-homolog
Nucleocapsid/KMKDLSPRWYFYYLG (100–114)	Positive	Inducer	Inducer	1.4297 (antigen)/non-allergen	Non-toxin	100	Non-homolog

### Prediction of linear and conformational B-cell epitopes

After carefully examining the results of 5 linear B-cell (LBL) epitope prediction servers and considering the similarities in their results, 27 epitopes were predicted for S protein, 14 for N, 12 for M, and 6 for E. 12 linear B-cell epitopes of S, 3 of N, 3 of M and 2 epitopes of E were obtained. They were chosen based on the binding score, antigenicity, allergenicity, toxicity, flexibility, hydrophilicity, accessibility on the surface, and complete conservation criteria (Table 4 and Supplementary Table S13). The positions of these LBL epitopes in the vaccine structure are shown in Supplementary Fig. S2. 9 epitopes were selected from these epitopes to be part of the final vaccine structure. Based on the results obtained from the ElliPro server, 3 B-cell conformational epitopes were also identified from the refined Three-dimensional (3D) model, which amino acid residues, sequence location, number of residues, and their scores are shown in Table 5. In Supplementary Fig. S3, a graphic representation of the 3D model of these epitopes is presented.

**Table 4:** Details of linear/continuous B-cell (LBL) epitopes in the vaccine construct.

Proteins	Epitopes (position)	Antigenicity/allergenicity	Toxicity	Epitope conservation (%)	Homology to human proteins
Spike	ITPGTNTSN (598–606)	1.0158 (antigen)/non-allergen	Non-toxin	100	Non-homolog
Spike	QSYGFQPTN (491–499)	1.4068 (antigen)/non-allergen	Non-toxin	100	Non-homolog
Spike	REPEDLPQG (208–216)	0.7997 (antigen)/non-allergen	Non-toxin	100	Non-homolog
Spike	STEIQAGNCYFP (469–480)	0.6932 (antigen)/non-allergen	Non-toxin	100	Non-homolog
Spike	IGSKPCNGVEGFN (487–499)	0.4402 (antigen)/non-allergen	Non-toxin	100	Non-homolog
Spike	NKPCNGVAGFNC (474–485)	0.5067 (antigen)/non-allergen	Non-toxin	100	Non-homolog
Nucleocapsid	RGGDGKMKD (95–103)	0.8805 (antigen)/non-allergen	Non-toxin	100	Non-homolog
Membrane	PLLESELVI (132–140)	0.5354 (antigen)/non-allergen	Non-toxin	100	Non-homolog
Envelope	RVKNLNSSR (61–69)	0.8998 (antigen)/non-allergen	Non-toxin	100	Non-homolog

**Table 5:** List of conformational/discontinuous B-cell epitopes predicted over final vaccine construct.

No	Residue	Number of residues	Score
1	_:A522, _:G523, _:N524, _:C525, _:Y526, _:F527, _:P528, _:K529, _:K530, _:I531, _:G532, _:S533, _:K534, _:P535, _:C536, _:E540, _:G541, _:F542, _:N543, _:K544, _:K545, _:N546, _:K547, _:P548, _:C549, _:N550, _:A553, _:G554, _:F555, _:N556, _:C557, _:H558, _:E559, _:H560, _:E561, _:H562, _:E563, _:H564, _:E565, _:H566	40	0.9
2	_:L67, _:A69, _:A70, _:G71, _:D72, _:K73, _:K74, _:I75, _:G76, _:V77, _:I78, _:K79, _:V80, _:V81, _:R82, _:E83, _:I84, _:V85, _:S86, _:G87, _:L88, _:G89, _:L90, _:K91, _:E92, _:A93, _:K94, _:D95, _:L96, _:V97, _:D98, _:G99, _:A100, _:P101, _:K102, _:L104, _:D114, _:E115, _:A116, _:K117, _:A118, _:K119, _:L120, _:E121, _:A122, _:A123, _:G124, _:A125, _:T126, _:V127	50	0.888
3	_:Y395, _:D396, _:Y397, _:E398, _:A399, _:R400, _:T401, _:E402, _:D403, _:D404, _:L405, _:S406, _:F407, _:H408, _:K409, _:E411, _:T430, _:T431, _:G432, _:E433, _:T434, _:G435, _:Y436, _:N470, _:R473, _:E474, _:P475, _:E476, _:D477, _:L478, _:P479, _:Q480, _:G481, _:K482, _:R484	35	0.847

### Analysis of human population coverage around the world, protection assessment, and autoimmunity identification

Population coverage analysis revealed that 12 T-cell epitopes selected in this study represented 96.77% of the worldwide human population. The percentage coverage of epitopes in other regions is also presented separately in Table 6 and Supplementary Fig. S4. Therefore, the designed multi-epitope vaccine can be recommended to combat SARS-CoV-2 in most regions (Supplementary Table S14). In this study, North America had the highest coverage (97.97%), while Central America had the least (36.29%). The low population coverage of these epitopes in Central America can be related to the fact that the populations of these regions are a complex array of different admixture processes with varying degrees of ancestral population proportions that came in different migration waves. So, results from population genetics comparisons show a wide variation in the HLA profiles from the populations that correlate with different admixture proportions^25^. Since Central America exhibits significant genetic diversity among its populations if a peptide vaccine is designed based on specific epitopes and these epitopes are not familiar or diverse in the genetic composition of Central American populations, it could lead to epitope coverage decrease. According to the IEDB conservation analysis tool results, as expected, selected T and B-cell epitopes in regions without alteration in the studied structural protein sequence showed high conservation among different variants (Supplementary Fig. S5). In addition, all selected epitopes were non-homologous with the human proteome, indicating the multi-epitope vaccine can induce a safe antigenic response but no cross-reaction with proteins in humans.

**Table 6:** Population coverage of the selected epitope included in the vaccine construct.

Population/area	Class combined		
	Coverage^a^	Average hit^b^	pc90^c^
World	96.77%	6.18	2.05
East Asia	96.58%	6.13	2.12
Northeast Asia	94.78%	4.66	1.41
South Asia	96.9%	6.04	2.01
Southeast Asia	91.84%	3.89	1.12
Southwest Asia	86.48%	3.71	0.74
Europe	97.87%	6.88	2.5
East Africa	90.14%	3.74	1.01
West Africa	91.73%	4.03	1.15
Central Africa	88.91%	3.59	0.9
North Africa	94.15%	5.04	1.49
South Africa	92.14%	3.28	1.12
West Indies	91.4%	4.11	1.12
North America	97.97%	6.48	2.61
Central America	36.29%	1.3	0.16
South America	86.76%	4.08	0.76
Oceania	89.12%	3.92	0.92
Average	89.4	4.53	1.36
Standard deviation	13.76	1.39	0.65

^a^Coverage of population on projected.

^b^Population recognized by HLA combinations/epitope hits on the average number.

^c^90% of the population recognized by HLA combinations/epitope hits on the minimum number.

### Making the final multi-epitope vaccine

**Figure 3:**
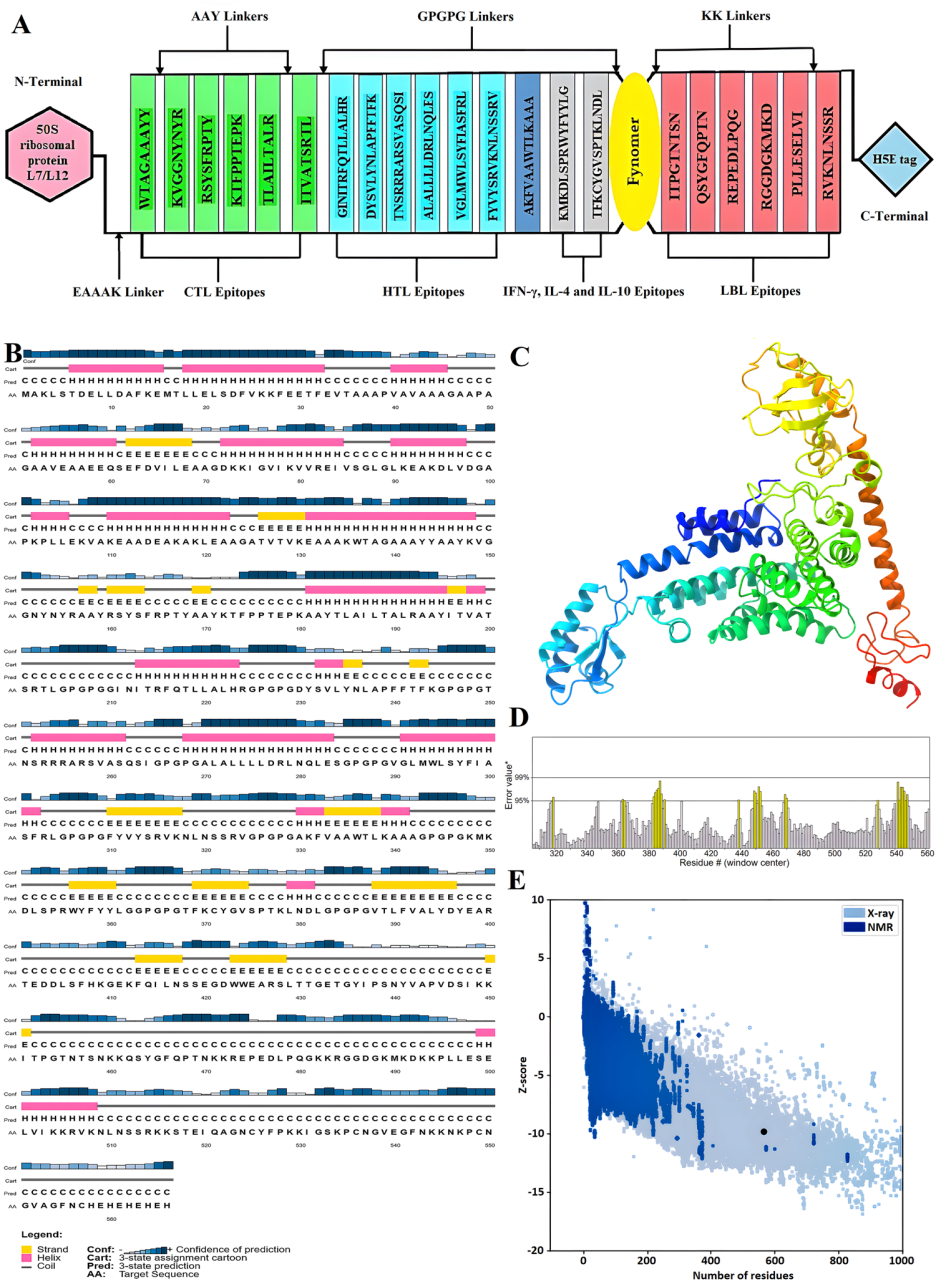

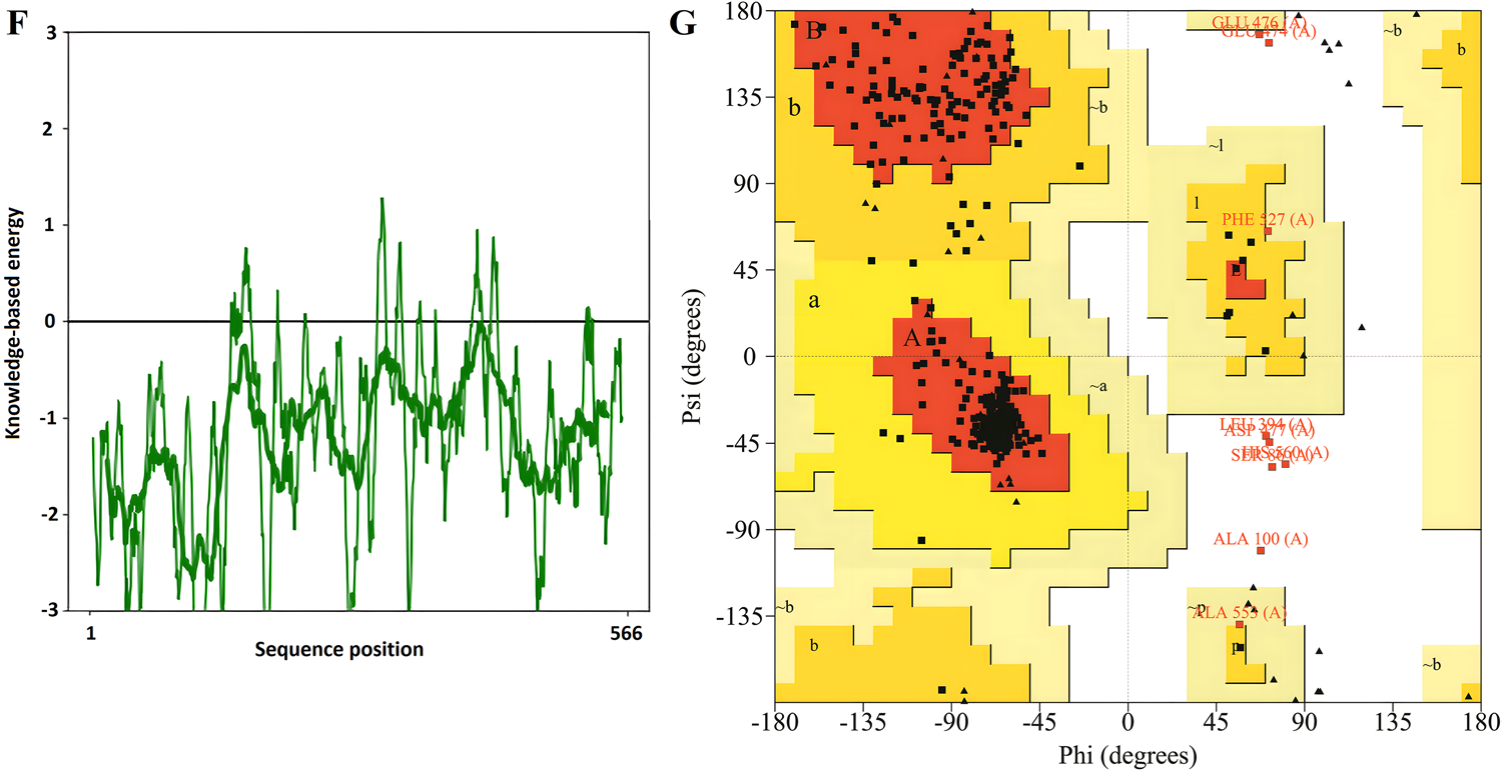
(**A**) Schematic Presentation of the final multi-epitope vaccine construct. (**B**) The secondary structural prediction of the vaccine. (**C**) The three-dimensional refined vaccine model is visualized to represent the helical, sheet, and loop regions. (**D**) Validation of the vaccine structure by ERRAT with a score of 95.5036. (**E**) ProSA validation of predicted structure with Z-score of −9.78 and (**F**) plots the residues scores to check the local model quality. (**G**) Analysis of the Ramachandran plot utilizing the PROCHECK server showed 94.6%, 3.5%, 0.4%, and 1.5% residues laying in favored, additional allowed, allowed, and disallowed regions, respectively.

The structure of the linear vaccine includes epitopes CTL, HTL, IFN-γ, IL-4, IL-10, LBL, Pan HLA-DR reactive epitope (PADRE), 50S ribosomal protein L7/L12 (rpIL) adjuvant, fynomer sequence, linkers (EAAAK, AAY, GPGPG, and KK) and finally the H5E tag. Figure 3A illustrates the general outline of this structure.

### Evaluation of antigenicity, allergenicity, toxicity, solubility, and physicochemical properties of the vaccine

Antigenicity, allergenicity, toxicity, and solubility were also assessed for the final sequence of the designed vaccine, and it met all the criteria. The vaccine's antigenicity was confirmed by VaxiJen v2.0 servers^26^ with a score of 0.6387 and ANTIGENpro^27^ with a score of 0.9238, indicating the final vaccine had a high antigenicity. AllergenFP 1.0 and AllerTOP 2.0 servers^28,29^ showed that the final vaccine is non-allergenic and does not induce allergic reactions. Based on the results of the SVM prediction mode in the ToxinPred server^30^, the entire final vaccine sequence, which includes the adjuvant sequence, all epitopes, linkers, fynomer sequence, and H5E tag, does not contain any toxin part. The values predicted by SolPro^31^ (0.7368) and Protein-sol server^32^ (0.496) also show that the vaccine has good solubility (Table 7). Solubility is a critical factor in vaccine development because it influences the distribution, stability, efficacy, vaccine formulation, administration, adsorption, manufacturing process, storage conditions, bioavailability of the vaccine components, and overall success of vaccination programs^33^.

**Table 7:** Antigenicity, allergenicity, solubility, toxicity, and physicochemical properties prediction results for final vaccine construction.

Final vaccine	Vaxijen score	Antigen pro score	Allergen FP/AllerTOP result	Solubility by SolPro	Solubility by protein-sol	Toxicity	Mol. weight (kDa)
	0.6387	0.9238	Non-allergen	0.7368	0.496	Non-Toxin	60.773

	Theoretical pI	Charge	Half-life (in vitro)	Half-life (in vivo)	Instability index	Aliphatic index	Hydropathicity (GRAVY)
	9.36	19.00	30 h	> 20 h > 10 h	34.09 (stable)	73.02	−0.368

According to the results of the evaluation of the physicochemical properties of the vaccine, the final structure has 566 residues and a molecular weight of 60.77 kDa. This structure has a theoretical isoelectric point (pI) of 9.36, indicating that it is essential. There were 56 negatively charged residues (Asp + Glu) and 75 positively charged residues (Arg + Lys). The charge is 19.00, which decreases in an alkaline environment, and it is generally preferable to have positive charge values. The coefficient of extinction is 71,740 M^−1^ cm^−1^, with absorption values (0.1%) (g/l) 1185, including all cysteine pairs under aqueous conditions at 280 nm. In addition, the estimated half-life of the vaccine in mammalian reticulocytes (in vitro) is 30 h, in yeast (in vivo) approximately 20 h, and in *E. coli* (in vivo) 10 h, which indicates the stability of the vaccine in different phases. The half-life of a vaccine varies across different organisms, indicating its stability and effectiveness in different biological environments. A shorter half-life may require more frequent administration, affecting the dosing schedule and practicality. However, a longer half-life can offer prolonged protection but may pose safety concerns or require rapid adaptation to emerging viral variants. The optimal half-life depends on the vaccine's nature, target pathogen, and practical administration considerations^34^. The prediction showed that the structure of the vaccine, with a 34.09 (< 40) instability index, has a high degree of stability to elicit an immune response. The aliphatic index of the vaccine is 73.02, which indicates it has a thermostable nature, and when the aliphatic index of a protein increases, it becomes resistant to heat. The overall average hydrophilicity (GRAVY) was −0.368, and lower GRAVY scores indicate better solubility, which suggests that the vaccine candidate is hydrophilic, improving interaction with blood and water and making it more straightforward to identify the target^35^. All the results of the physicochemical properties analysis supported that the vaccine structure meets the criteria for vaccine formulation (Table 7 and Supplementary Material SM1). Since the designed vaccine lacs any transmembrane helices, a problem regarding expression in vaccine production is not expected. Additionally, the absence of signal peptides in the vaccine structure indicates the prevention of protein localization^36^ (Supplementary Figs. S7 and S8).

### The secondary structure of the vaccine construct

The secondary structure of the vaccine was predicted using PSI-blast-based secondary structure prediction (PSIPRED)^37^, and it was found to consist of 32.50% helix, 22.09% strand, and 54.59% coil. Additionally, the secondary structure was provided by the Self-Optimized Prediction Method with Alignment (SOPMA)^38^ tool with default parameters including 39.39% alpha-helix, 18.20% extended strand, 5.83% beta-turn, and 42.58% random coil. A graphical representation of the secondary structure features is shown in Fig. 3B. The high percentage of random coil, as can be understood from the figure, indicates the presence of epitopes in different regions of the construct^39^.

### Modeling, refinement, and validation of the 3D structure of multi-epitope vaccine

After creating the 3D vaccine model using the Robetta server^40^, one model was selected as the best prediction model, and based on this model, the 3D structure of the final vaccine was made. Later than refining the 3D structural model obtained from the modeling stage by GalaxyRefne server^41^, model 1 was selected as the best final vaccine model based on various parameters, including GDT-HA (0.9832), RMSD (0.287), MolProbity (1.954), Clash score (15.9) and weak rotamers (0.5) among other refined models (Fig. 3C and Supplementary Table S15). The Z-score for the vaccine structure in the PROSA diagram was −9.78. This score is close to the range of native proteins of similar size, indicating the lowest error rate and accuracy of the simulation and the overall reliability of the predicted model (Fig. 3E, F). In addition, the quality factor of 95.5036 obtained using the ERRAT server indicates the optimal quality of the protein model^42^ (Fig. 3D). Grouping of amino acids based on phi and psi angles by Ramachandran plot^43,44^ using PROCHECK analysis in the PDBsum server for the refined structure, revealed that 94.6% of the residues were classified in the most favorable, 3.5% additional allowed, 0.4% generously allowed, and 1.5% disallowed region (Fig. 3G). According to the results, the total residuals in the desired area were in the ideal value range, i.e., more than 90%, which confirms the reliability of this model.

### Molecular docking of the vaccine construct with TLR3, TLR4, MHC-I, and II receptors and binding affinity evaluation

The process of protein–protein docking between refined 3D models of the final vaccine with immune receptors TLR2 and TLR4 and MHC-I and II receptors, utilizing the HADDOCK web server 2.4, classifieds the structures as refined models with water into multiple clusters by percentage values. The most reliable clusters are those whose complexes have the lowest HADDOCK score^45^. Table 8 displays the statistical parameters and their respective values for each docked vaccine-receptor complex. Supplementary Figs. S9-S12 shows the HADDOCK Refinement interface server's graphical results for each complex. The HADDOCK score, RMSD, and other presented criteria were evaluated for complexes (Table 9). Better connection is indicated by a low HADDOCK score (negative score)^46^. Also, the buried surface area (BSA) indicates the proximity and less surface of the protein exposed to water. On the other hand, the RMSD scores allow identifying the sets with the lowest energy and minimum structural deviation. Furthermore, the low RMSD score for the docked complexes represents a good-quality model.

**Table 8:** Statistics of the highest-scored vaccine-TLR2, TLR4, MHC-I, and MHC-II docked clusters. The most reliable cluster was selected based HADDOCK score for each complex. A negative value of the HADDOCK score projects good protein–protein interaction potential.

Complexes	Vac-TLR2	Vac-TLR4	Vac-MHC-I	Vac-MHC-II
HADDOCK score (kJ mol^−1^)	−143.8 ± 3.7	−141.9 ± 3.4	−93.1 ± 11.8	−74.0 ± 6.1
Cluster size (nm)	116	23	8	18
RMSD from the overall lowest energy structure (nm)	2.8 ± 1.6	0.7 ± 0.4	0.6 ± 0.3	25.5 ± 1.3
Van der Waals energy (kJ mol^−1^)	−112.3 ± 3.9	−100.2 ± 5.8	−93.4 ± 10.7	−84.7 ± 7.8
Electrostatic energy (kJ mol^−1^)	−337.2 ± 29.0	−428.1 ± 32.4	−439.5 ± 39.3	−226.9 ± 18.6
Desolvation energy (kJ mol^−1^)	−13.8 ± 2.7	−19.7 ± 6.0	−1.4 ± 3.0	−28.0 ± 5.7
Restraints violation energy (kJ mol^−1^)	498.3 ± 31.7	635.9 ± 30.0	895.1 ± 67.0	840.4 ± 61.2
Buried surface area	3737.9 ± 70.6	3128.7 ± 84.0	3019.9 ± 156.7	2661.8 ± 143.3
Z-score	−2.5	−2.7	−1.9	−1.8

**Table 9:** Statistics of a single cluster were obtained after applying refinements on top-ranked docked vaccine-TLR2, TLR4, MHC-I, and MHC-II structures.

Complexes	Vac-TLR2	Vac-TLR4	Vac-MHC-I	Vac-MHC-II
HADDOCK score (kJ mol^−1^)	−211.6 ± 2.4	−234.4 ± 2.2	−227.0 ± 3.4	−181.9 ± 2.3
Cluster size (nm)	20	20	20	20
RMSD from the overall lowest energy structure (nm)	0.6 ± 0.4	0.6 ± 0.3	0.6 ± 0.4	0.6 ± 0.4
Van der Waals energy (kJ mol^−1^)	−129.0 ± 2.7	−111.3 ± 7.8	−107.2 ± 8.3	−92.1 ± 5.9
Electrostatic energy (kJ mol^−1^)	−349.7 ± 31.1	−504.1 ± 21.7	−613.6 ± 30.6	−261.0 ± 20.0
Desolvation energy (kJ mol^−1^)	−12.7 ± 3.1	−22.2 ± 2.8	2.9 ± 1.3	−37.6 ± 4.0
Restraints violation energy (kJ mol^−1^)	0.0 ± 0.0	0.0 ± 0.0	0.0 ± 0.0	0.0 ± 0.0
Buried surface area	3919.4 ± 85.7	3088.6 ± 24.0	3353.8 ± 30.8	2989.4 ± 87.4
Z-score	0.0	0.0	0.0	0.0

**Figure 4:**
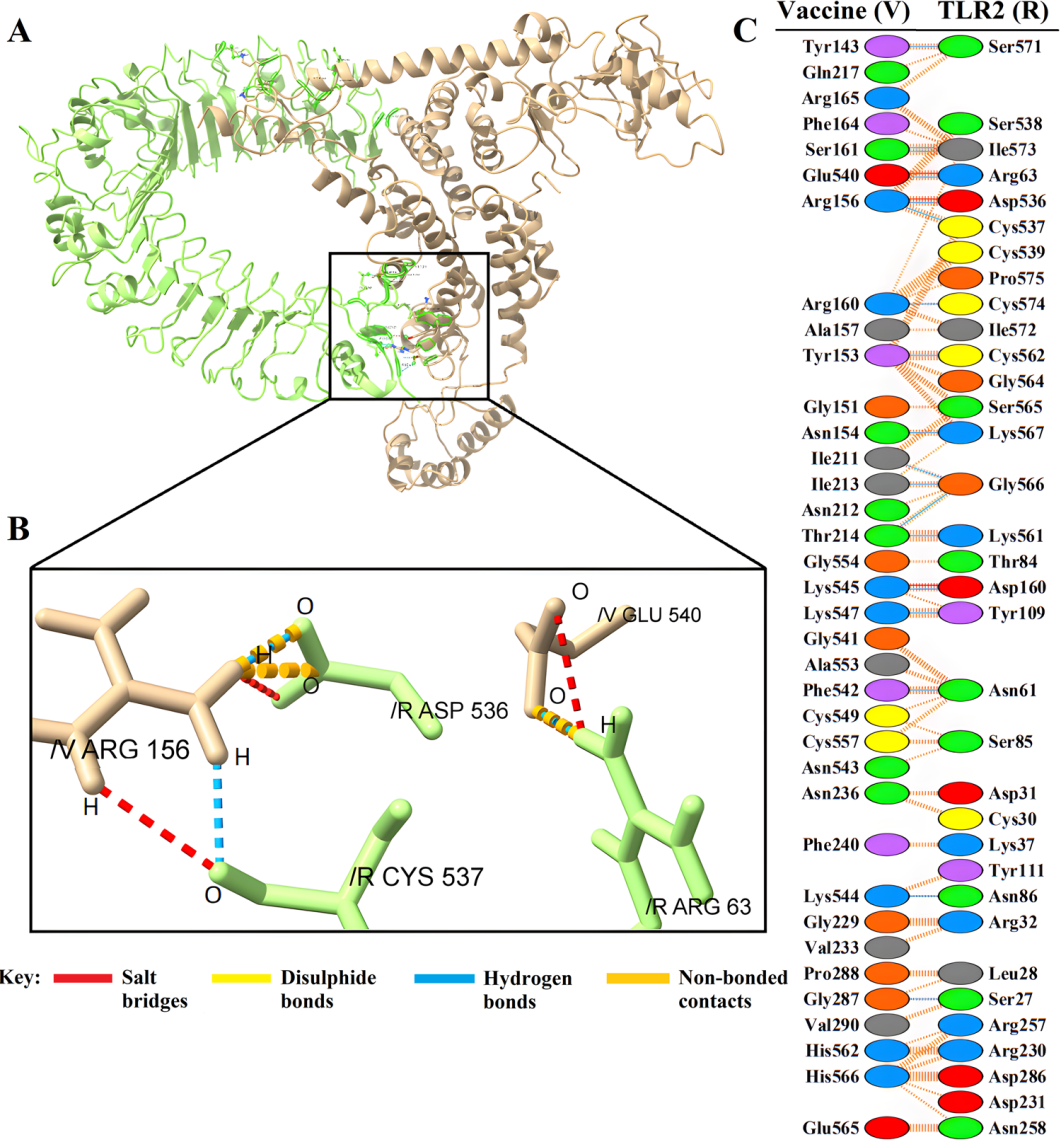
(**A**) Visualization of docking results for the vaccine-TLR2 complex. The vaccine construct is shown in gold, while TLR2 is depicted in green. (**B**) Magnified residues and their atoms are shown as sticks and labeled with chain, code, and number. Hydrogen bonds, salt bridges, and other interactions were represented by colored dashed lines. (**C**) Map of total interacting residues and bonds between the vaccine and TLR2 protein chains.

**Figure 5:**
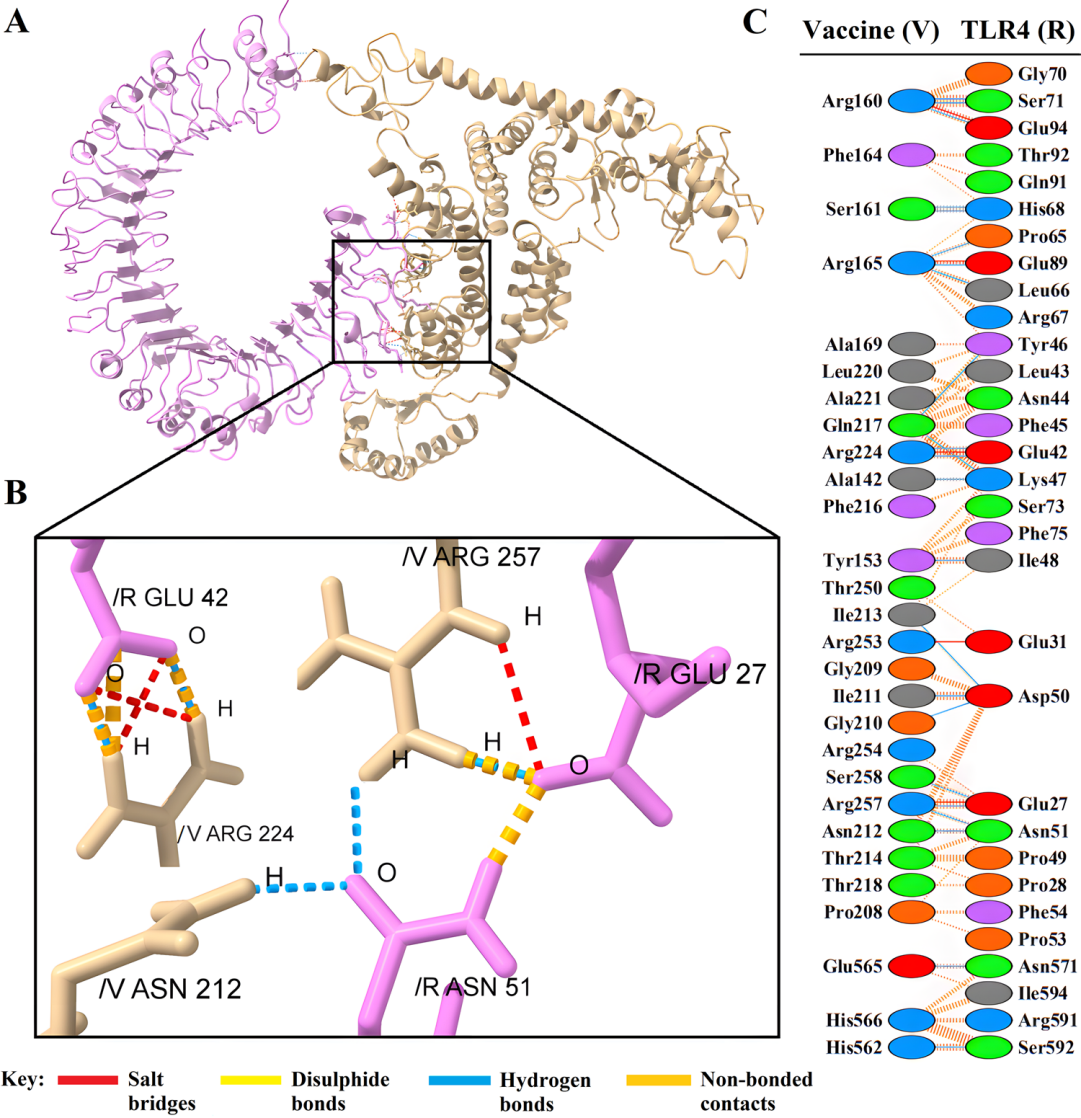
(**A**) Visualization of docking results for the vaccine-TLR4 complex. The vaccine construct is shown in gold, while TLR4 is depicted in violet. (**B**) Magnified residues and their atoms are shown as sticks and labeled with chain, code, and number. Hydrogen bonds, salt bridges, and other interactions were represented by colored dashed lines. (**C**) Map of total interacting residues and bonds between the vaccine and TLR4 protein chains.

**Figure 6:**
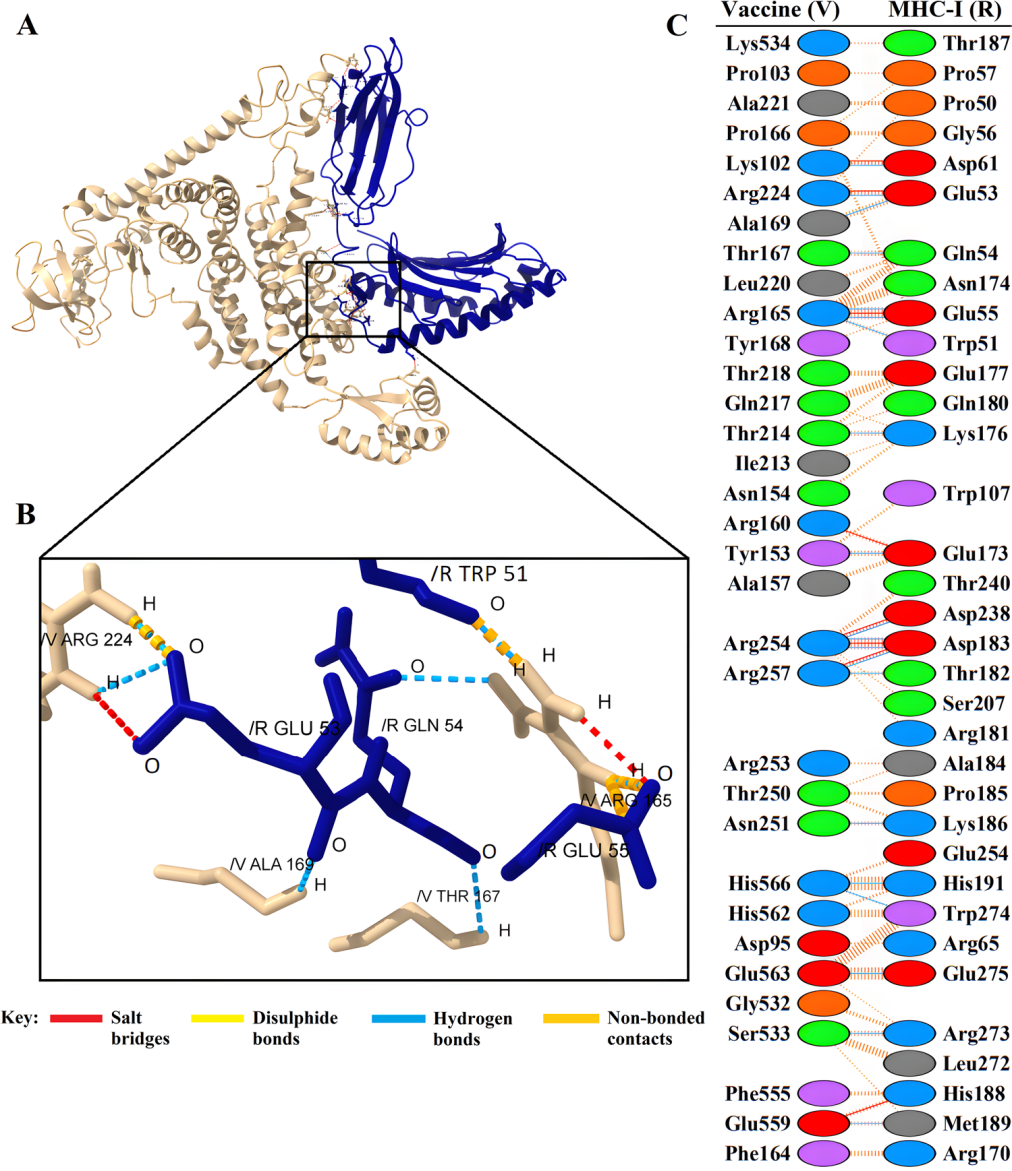
(**A**) Visualization of docking results for the vaccine-MHC-I complex. The vaccine construct is shown in gold, while MHC-I is depicted in navy. (**B**) Magnified residues and their atoms are shown as sticks and labeled with chain, code, and number. Hydrogen bonds, salt bridges, and other interactions were represented by colored dashed lines. (**C**) Map of total interacting residues and bonds between the vaccine and MHC-I protein chains.

**Figure 7:**
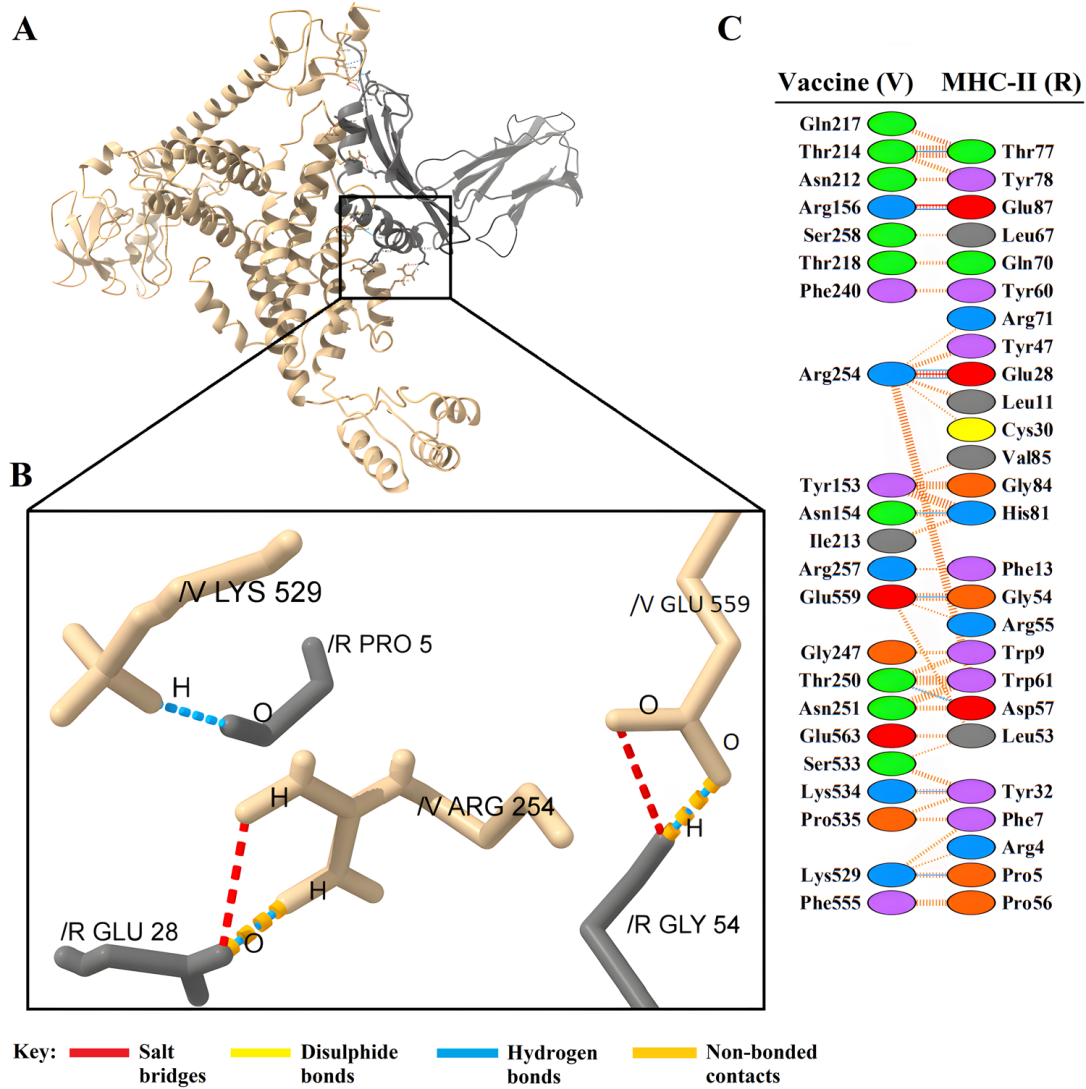
(**A**) Visualization of docking results for the vaccine-MHC-II complex. The vaccine construct is shown in gold, while MHC-II is depicted in gray. (**B**) Magnified residues and their atoms are shown as sticks and labeled with chain, code, and number. Hydrogen bonds, salt bridges, and other interactions were represented by colored dashed lines. (**C**) Map of total interacting residues and bonds between the vaccine and MHC-II protein chains.

An overview of molecular docking and the amino acids that directly interact at the binding sites of the vaccine structure and receptors are shown in Figs. 4A, B, 5A, B, 6A, B, 7A, B. Amino acids and, in more detail, their atoms predicted to be involved in vaccine-receptor interactions are presented in Supplementary Materials S2–S5.

According to PDBsum results, there were 36 residues of the vaccine and 34 residues of TLR2 in the complex between the vaccine and TLR2, and the interface area (Å^2^) for the vaccine and TLR2 was 1965 and 1953, respectively. For the vaccine-TLR4 complex, 28 vaccine residues interacted with 31 residues of TLR3, and the interface area was 1552 Å^2^ for the vaccine and 1457 Å^2^ for TLR4. The interacting residues of the vaccine-MHC-I complex were 33 residues for the vaccine and 34 residues for MHC-I, and the interface region was 1677 Å^2^ for the vaccine and 1606 Å^2^ for MHC-I. Also, for the vaccine-MHC-II complex, 22 vaccine residues interacted with 26 MHC-II residues, and the interface region for the vaccine was 1426 Å^2^, while this region was 1358 Å^2^ for MHC-II. On the other hand, the molecular interaction established between the vaccine with TLR2; 3 salt bridges, 18 hydrogen bonds, and 155 non-bonding contacts; with TLR4, 5 salt bridges, 23 hydrogen bonds, and 187 non-bonding contacts; with MHC-I, 8 salt bridges, 20 hydrogen bonds and 196 non-bonded contacts and finally with MHC-II; there were 2 salt bridges, 9 hydrogen bonds and 113 non-bonded contacts (Figs. 4C, 5C, 6C, and 7C). Hydrogen bonds are essential in molecular recognition because they are important in achieving a stable compound^47^. Electrostatic bonds also play an essential role in the interactions produced in complex^47^. Since, in this study, most of the hydrogen bond distances between vaccine-receptor interacting residues are around 2–3 Å, there is a high interaction between them^48^. In protein–protein complexes, salt bridges can be crucial for maintaining the stability of the interaction interface. Also, they can contribute to the specificity and affinity of the interaction between two proteins and be involved in the recognition and binding of the interacting partners^49^. In addition, the Ramachandran plots presented in the PDBsum results were also investigated for structural validation of the docked sets, which were also confirmatory (Supplementary Figs. S13–S16).

After binding affinity analysis using the PRODIGY (PROtein binDIng enerGY prediction) web server, ΔG values (Gibbs free energy) for TLR2-vaccine, TLR4-vaccine, MHC-I-vaccine, and MHC-II-vaccine complex were obtained −18.4, −11.2, −13.7, −9.7 kcal mol^−1^ (kilocalories per mole), respectively. The negative values of ∆G indicate that all four docked complexes are energetically feasible. The dissociation constant (Kd) presents binding affinity and bond strength. As binding affinity and Kd share an inverse correlation, a lower Kd signifies a heightened binding affinity. This implies that vaccine-receptor bonds are securely and tightly bound when the dissociation constant is lower (Table 10).

**Table 10:** Binding affinities of the docked complexes of the vaccine with TLR2, TLR4, MHC-I and MHC-II, as predicted by the PRODIGY server.

Complexes	Gibbs free energy (kcal mol^−1^)	Kd (M)
Vaccine-TLR2	− 18.4	1.1E−13
Vaccine-TLR4	− 13.7	2.2E−10
Vaccine-MHC class I receptor	− 11.8	4.5E−09
Vaccine-MHC class II receptor	− 9.7	1.3E−07

### Energy minimization and molecular dynamics simulation of vaccine structure

**Figure 8:**
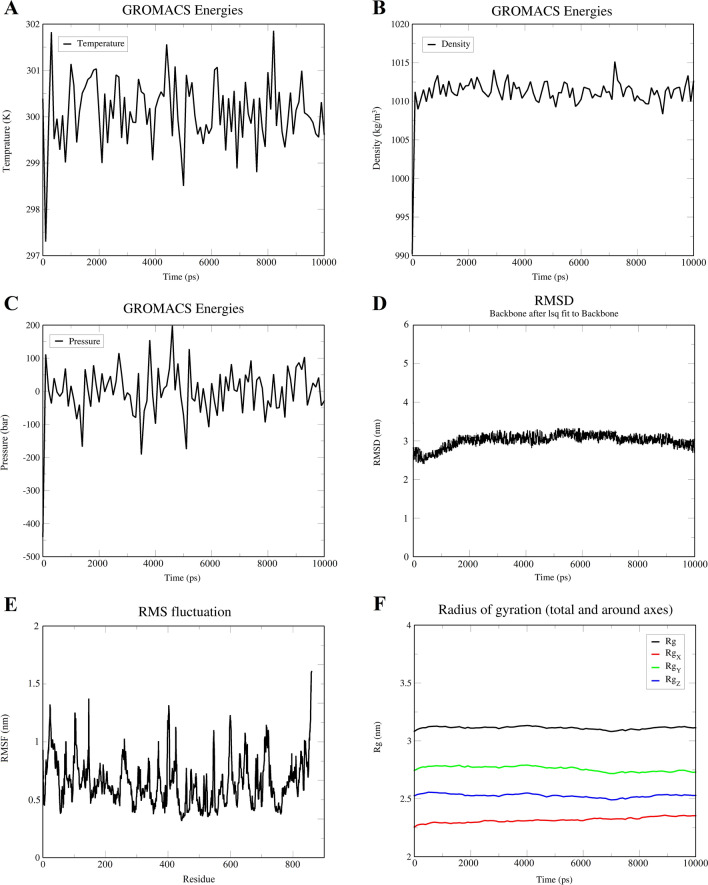
The results of molecular dynamics simulation of vaccine for analysis of structural stability. (**A**) Graph showing the equilibrated temperature during energy minimization. (**B**) Graphical presentation of density during simulation. (**C**) Graph showing the pressure of the system during simulation. (D) RMSD plot of the vaccine construct indicating stability. (**E**) RMSF plot illustrates high fluctuations, the peak-like regions with a higher degree of flexibility. (**F**) The Rg plot showing the vaccine construct stays compact around its axes, supporting its stability during simulation.

Molecular dynamics simulation (MDS), a technique for studying the atomic behavior of molecular systems, was employed in this study to estimate and evaluate the stability and physical movements of the vaccine structure under various conditions^50^. The purpose of molecular dynamics simulation for the designed vaccine is to observe how it functions in a real-life biological system^51^. With the aid of GROMACS software, some analyses, including energy minimization, pressure assessment, temperature, and potential energy calculations, were carried out^52^. Through the steepest descent algorithm, energy minimization was performed for the vaccine, and the energy of the protein was considered minimized when it was less than 1000 kJ mol^−1^. Energy minimization was applied for 2,482 steps, where the force reached less than 1000 kJ mol^−1^. The system's potential energy was computed as −3.06 e5228478 kJ mol^−1^ with a total drift of −300,757 kJ mol^−1^ and an average potential energy of −3.06 e43701 kJ mol^−1^. The average temperature at the end of 5000 NVT steps was 299.85 K, with a temperature drift of 1.14 K (Fig. 8A). The calculated average density of the system was 1011.18 kg m^−3^ with a total drift of 0.154 kg m^−3^ (Fig. 8B). The pressure of the system was 0.327 bar with a total drift of 4.57 bar (Fig. 8C). After a simulation time of 10 ns, a trajectory analysis was performed to confirm the flexibility and stability of the candidate vaccine. The RMSD plot illustrates the fluctuations in the overall of the vaccine and conveys its stability over time. During the 10 ns simulation process, the RMSD of the vaccine has relatively mild fluctuations, which indicates the construct is stable (Fig. 8D). The RMSF and particularly the high peaks in the RMSF demonstrate the flexibility in the vaccine structure. The RMSF plot, particularly the high peaks in it, demonstrates the flexibility of the vaccine structure (Fig. 8E). Also, the stability and compactness of the structure during the simulation have been confirmed by the radius of the gyration plot (Rg) (Fig. 8F).

### Codon optimization and in-silico cloning of vaccine

**Figure 9:**
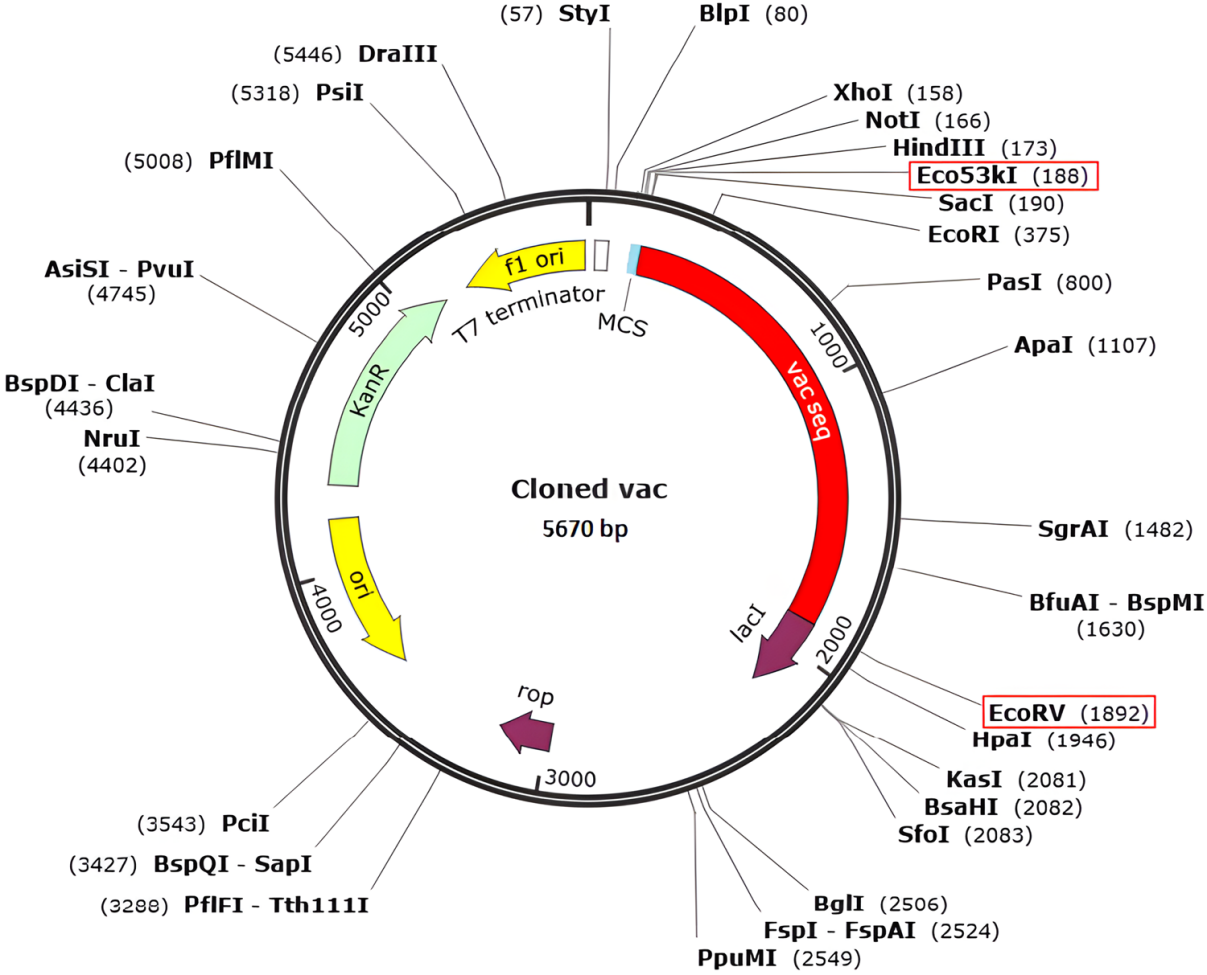
In silico restriction cloning of the designed vaccine into the pET-28a (+) expression vector. The red bar represents the codon-optimized gene of the vaccine, and the black circle represents the vector backbone.

The candidate vaccine nucleotide sequence was obtained after codon optimization to maximize protein expression in the *E. coli* strain K12 as the expression host organism^53^. For the 1698 nucleotide optimized codon sequence, the values for the guanine-cytosine content (GC-Content) and codon adaptation index (CAI Value) were 0.9335 and 50.73, respectively (Supplementary Material SM6). Generally, a CAI above 0.8 or almost 154 and GC between 30 and 70%^35^ indicates good protein expression in the host system. This also supports the practical expression of the vaccine formulated in this strain. Figure 9, the SnapGene software output, shows the cloning of the vaccine sequence inside the pET-28a ( +) vector to generate a recombinant plasmid for developing an efficient in silico cloning strategy.

### Immune simulation

**Figure 10:**
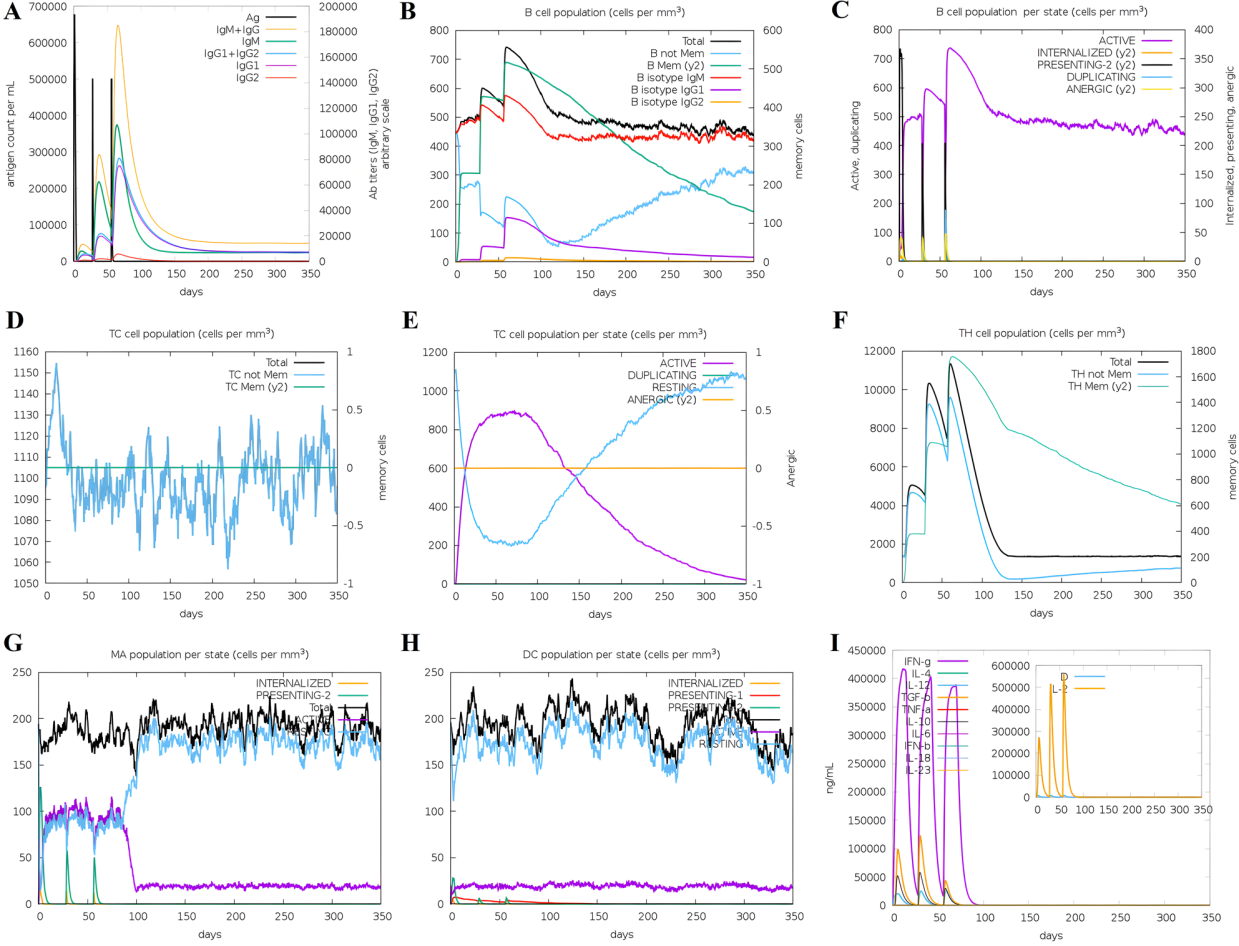
In silico simulation of immune response triggered by the designed vaccine as an antigen after three subsequent injections. (**A**) Antigen and subtypes of immunoglobulin levels are represented as different colored peaks. The Immunoglobulin (IgG) production represents the proliferation of primary, secondary, and tertiary immune responses after the vaccine administration. (**B,C**) B lymphocytes by total count and population per entity state (active, presenting, internalized, duplicating, or anergic). (**D**) Cytotoxic T-cell population. (**E**) Cytotoxic T-cell population per state. (**F**) Helper T-cell population. (**G**) Macrophages population per state. (**H**) Dendritic cell population per state. (**I**) The concentration of cytokines and interleukins is at three different stages.

Based on immunological simulation data, administration of this vaccine with three injections potentially induces a range of immunoglobulins^54^ (Fig. 10). In the initial response, the level of IgM increases, while in the second and third responses produced by the simulation, the levels of IgM + IgG, IgG1 + IgG2, IgG1, and IgG2 were significantly higher than in the first response. Subsequent administration of three vaccine injections resulted in a decrease in the concentration of specific antigens with typically high immunoglobulin concentrations, i.e., IgG1 + IgG2, IgM, and IgG + IgM (Fig. 10A). In addition, some high-stability B-cell isotypes are identified that provide the potential for isotype switching and memory formation (Fig. 10B, C). There was also an increase in the production of CTL/HTL cells and memory cells (TCs) during vaccination, indicating immunogenicity in the presence of T-cell epitopes in the vaccine framework (Fig. 10D–F). In general, according to these results, after the initial activation and proliferation of immune cells, the immune system undergoes a contraction phase, which leads to a decrease in some immunoglobulins, including IgM and IgG, between time intervals after vaccination. This reduction in response does not necessarily indicate vaccine failure, and it is a normal part of the immune response. In these conditions, other immune system components, such as memory T and B-cells, play an essential role in maintaining immunity. By creating immunological memory, they enable faster and more efficient responses in re-exposure to future pathogens, which depends on the incubation time of the infection, memory response quality, and memory B-cell antibody levels. Therefore, even if the level of some antibodies decreases after the initial response, the immune system is not defenseless^55^. Also, each time of exposure, there was an increase in natural killer cells (NK cells), dendritic cells (DCs), and macrophage activities (Fig. 10G, H). High levels of IFN-γ, IL-23, IL-10, and IL-12 are markers of a favorable immune response, which increased significantly after exposure (Fig. 10I). Finally, immune simulations for 3 and 12 doses confirm that the vaccine induces a robust immune response and that the level of immunity increases even with repeated exposures in pandemic situations. It is important to note that interpreting vaccine efficacy and protection involves a comprehensive assessment of various immune components, including antibodies, memory cells, and other immune responses. Additionally, the specific dynamics of the immune response may vary depending on the vaccine and the individual characteristics of the immune system^56^.

## Discussion

The SARS-CoV-2 pandemic has raised many questions about human weakness and susceptibility to this pathogen and viral diseases that may emerge. COVID-19 has inspired researchers to develop effective drugs and up-to-date vaccines to help control the epidemic because it threatens public health^57^. Despite the chance that new variants could emerge as a result of genetic mutations in the SARS-CoV-2 genome, it is evident that there is a lot more research that needs to be done on potential candidates. However, new vaccine candidates may have fewer or milder side effects, which would satisfy those concerned about the severity of side effects from other vaccines^58^. Using live or attenuated viral vaccines has several drawbacks, like the risk of reversion to a more acute strain, being expensive, being time-consuming, causing allergic reactions, and having other destructive immunological responses^59^. So, ensuring you are getting a safe and effective vaccine is essential. Designing epitope-based vaccines in silico alongside immunoinformatics approaches is highly efficient because it enables researchers to quickly predict and accurately evaluate potential antigenic epitopes while saving time and money^47^.

We designed a multi-epitope vaccine based on a fynomer antibody mimetic that can induce a robust immune response against SARS-CoV-2 and is a candidate to prevent the COVID-19 pandemic. With the help of computational tools and molecular modeling, it is also possible to generate humoral and cellular immunity as immune responses for a set of short immunogenic sequences, i.e., epitopes, instead of large proteins or whole genomes that are typically employed in recombinant vaccine technology^60,61^. It is important to note that the specific design of a vaccine depends on the characteristics of the target virus, the goals of the vaccination strategy, and ongoing research findings. Researchers and vaccine developers carefully consider these factors when creating vaccines that offer the best protection against the targeted pathogen^62^. This study has provided a comprehensive and improved understanding of the design of a vaccine against the coronavirus due to the utilization of four structural proteins from various coronavirus strains to screen for T and B-cell epitopes. The decision to include additional proteins in a vaccine design alongside the spike protein is often strategic based on various considerations. While the spike protein is a significant target for immune response due to its high antigenicity, other reasons, including broad protection, stability, efficacy, enhanced immune response, reducing escape mutants, and pre-existing immunity, are considered for the inclusion of other components in the vaccine^63^. Including multiple proteins in a vaccine offers several advantages. It broadens the immune response, providing potential protection against various virus variants. This strategy safeguards against mutations in the spike protein by ensuring other components can still trigger an immune response. Different proteins may offer enhanced stability, production feasibility, or immunogenicity, optimizing vaccine efficacy. Diversifying antigens stimulates various aspects of the immune system, potentially yielding a more robust and comprehensive response for improved protection. Including other proteins reduces the risk of the virus developing escape mutants, enhancing immune detection. Additionally, accounting for pre-existing immunity to the spike protein by incorporating other proteins can boost overall vaccine effectiveness^64^. The epitopes derived from the structural proteins used in the analysis of T lymphocytes tended to interact with a variety of MHC-I and MHC-II alleles. Epitopes were prioritized in conservation, antigenicity, allergenicity, non-toxicity, comprehensive coverage in human populations, lack of homology with human proteins, and effective molecular interaction with the relevant HLA alleles. They were selected as candidate CTL and HTL epitopes for the vaccine (Tables 1, 2 and 3). Additionally, predictive tools were employed to evaluate B-cell epitopes for linearity, surface accessibility, antigenicity, flexibility, allergenicity, and toxicity (Table 4).

The final product of this study was a vaccine with a 566 amino acid length that included 6 CTL epitopes, 6 HTL epitopes, 2 stimulating epitopes IFN-γ, IL-4 and IL-10, 9 LBL epitopes, fynomer sequence with 61 amino acids, 50S ribosomal protein L7/L12 adjuvant, PADRE sequence and an H5E tag (Fig. 3A). With the aid of the appropriate tools, the vaccine structure was also assessed for antigenicity, sensitivity, toxicity, physicochemical properties, solubility, and the presence or absence of transmembrane helices (TMHs) and signal peptides^36^. All of the scores in different parameters for this proposed construct increase the likelihood that this vaccine can be shown to be safe and effective against SARS-CoV-2 and confirm its applicability (Table 7). The second and third structures of the multi-epitope construct play a critical role in vaccine formulation, and one of the fundamental challenges of structural biology is to identify discrepancies between experimental and theoretical predictions of protein structures^65^. Secondary structure analysis showed that the vaccine structure includes alpha helices, extended strands, beta turns, and random coiled structures, and identified which of these structures were present in the sequence amino acid regions provided to the software (Fig. 3B). The 3D structure of the vaccine was predicted by reliable software based on deep learning and then reconstructed with the help of other software in terms of conformational shape, energy minimization, and atom distance (Fig. 3C). Refinement and validation of the vaccine model showed that the quality of the predicted model was good. The result was also confirmed by Ramachandran plot predictions^66^ (Figs. 3D–G).

Protein–protein docking aimed to investigate the interaction and binding affinity of the vaccine structure with TLR2, TLR4, and MHC class I and II receptors. The expression of TLR2 and TLR4 receptors in the plasma membrane of different cell types, including immune cells, is linked to identifying pathogen molecular patterns and triggers of the immune response. By presenting microbe peptide fragments or vaccine epitopes on their surface, MHC molecules allow T lymphocytes to identify cells such as macrophages and stimulate and activate the immune response^67^. The docking results in the absorption of binding energy between the complexes, indicating a high degree of binding affinity, efficient docking, and stability of the complexes, which are expressed as negative binding energy values^68^ (Tables 8, 9 and 10). In the docking analysis of the vaccine with receptors, the presence of salt bridges and hydrogen bonds is proof of the existence of interaction (Figs. 4, 5, 6 and 7). Studies on the structural integrity of the vaccine in a simulation environment closely related to natural systems were performed by MDS. This structure is characterized by its system balance, stability, and high flexibility, as demonstrated by the results of plots RMSD, RMSF, and Rg, which align with the findings of other studies^69^. In order to imitate the usual immune responses, an immune simulation was performed, and simultaneously with the repeated injection of the vaccine, a significant increase in immunoglobulins was observed. This means that memory B-cells have been created. In addition, elevated levels of CTL, HTL, and IFN-γ are critical in ensuring a humoral and adaptable immune response^70^. Due to an overall improvement in the immune response to the second and third doses of the vaccine, the immune simulation has produced results consistent with the anticipated immune response. In other words, three injections generated a sufficient immunogenic response (Fig. 10). However, if the vaccine is administered through 12 consecutive injections, the immune system will respond more strongly (Supplementary Fig. S17). The expression of the vaccine construct in a suitable expression vector is necessary for the production of recombinant proteins^71^. In this regard, the sequence was reverse transcribed and adapted for the *E. coli* strain K12 before cloning into the pET-28a ( +) vector to ensure the translation efficiency of the designed vaccine construct in a specific expression system. An appropriate codon adaptation index and high GC content suggest a high protein expression level in the host. Finally, the gene of the vaccine construct, which has cleavage sites for specific restriction enzymes, was successfully cloned in the vector's multiple cloning site (MCS) (Fig. 9).

From the immunoinformatic approaches used in this study for SARS-CoV-2 in the design of multi-epitope vaccines against other pathogens such as MERS-CoV^47^, Nipah virus^72^, Zika^73^, dengue^74^, Hendra virus^75^, Malaria^76^, *Acinetobacter baumannii*^77^, *Leishmania infantum*^78^, *Pseudomonas aeruginosa*^79^, *Klebsiella pneumoniae*^80^, Kaposi sarcoma^81^ and several other examples have also been used. A similar strategy has also been used to make a vaccine against cancer antigens. The results obtained from computational and immunoinformatics tools and molecular dynamics simulation analyses confirm the reduction of antigen growth, the induction of appropriate immune response, and the safety and stability of the candidate vaccine. Therefore, the multi-epitope vaccine designed based on fynomer has promising results against SARS-COV-2, which requires further studies in vitro conditions to understand the efficacy and validation of the vaccine.

## Conclusions

The present study employed computational approaches to design a multi-epitope vaccine based on four viral structural proteins, conserved epitopes, and a fynomer antibody mimetic scaffold. These vaccines with such structures can be used to describe the immunogenic target in a novel way. This vaccine had favorable immunological properties with the ability to stimulate the innate and acquired immune systems and high population coverage. It also displayed acceptable physicochemical properties, stable structures, and proper binding with immune receptors. Thus, the product can be presented as a potential vaccine against the COVID-19 outbreak. However, these computational works require experimental validation to prove their effectiveness, which is possible by conducting additional studies and clinical trials at the in vitro and in vivo levels to determine the immunogenicity and safety of the vaccine.

## Methods

### Retrieval of SARS-CoV-2 protein sequences and antigenicity prediction

First, the complete sequences of structural proteins S, N, M, and E from the SARS-CoV-2 proteome for 32 different SARS-CoV-2 strains, including the variants Wuhan-Hu-1^8^, B.1.1.7 (Alpha), B.1.351 (Beta), P.1 (Gamma), B.1.617.2 (Delta), B.1.1.529 (Omicron), BA.1 (Omicron), BA.2 (Omicron), BA.2.12.1 (Omicron), BA.2.75 (Omicron), BA.4 (Omicron), BA.5 (Omicron), BQ.1.1 (Omicron), XBB.1.5 (Omicron), B.1.1.318, B.1.1.519, B.1.1.523, B.1.214.2, B. 1.427 or B.1.429 (Epsilon), B.1.466.2, B.1.525 (Eta), B.1.526 (Iota), B.1.617.1 (Kappa), B.1.617.3, B.1.619 or B.1.619.1, B.1.620, B.1.621 (Mu), C.1.2, C.37 (Lambda) C.36.3 or C.36.3.1, P.2 (Zeta) and, R.1 from the National Center for Biotechnology Information (NCBI) database (http://www.ncbi.nlm.nih.gov/genbank/), Bacterial and Viral Bioinformatics Resource Center (BV-BRC) (https://www.bv-brc.org) and ViralZone from Expasy (https://viralzone.expasy.org) were retrieved in FASTA format to use these sequences in predicting suitable epitopes for the making a multi-epitope vaccine against COVID-19. Additionally, the accuracy of some of these stored sequences was verified by similar sequences in the UniProtKB/Swiss-Prot (https://www.expasy.org/resources/uniprotkb-swiss-prot).

The antigenicity of these structural proteins was predicted using ANTIGENpro on scratch protein (http://scratch.proteomics.ics.uci.edu/) and VaxiJen (http://www.ddg-pharmfac.net/vaxijen/Vax iJen/VaxiJen.html). In ANTIGENpro, a threshold above 0.8^82^ and VaxiJen above 0.4^83^ for the viral category is considered to test the antigen level. In the following stage of the study, sequences with scores above the threshold were further analyzed. A set of predicted CTL, HTL, and LBL epitopes were included in an epitope bank created using these amino acid sequences.

### Prediction of T-cell epitopes

CTL epitopes using the NetCTL 1.2 server^84^ (https://www.cbs.dtu.dk/services/NetCTL/) and the Immune Epitope Database (IEDB) servers^85^ (https://tools.iedb.org/mhci/) were predicted. Epitope prediction by NetCTL is performed with 94–99% specificity and 54–89% sensitivity^86^. In this study, the most prevalent HLA class I alleles (A1, A2, A3, A24, A26, B7, B8, B27, B39, B44, B58, and B62) were tested on all nine amino acid peptide sequences to predict and evaluate potential CTL epitopes^87^. For the TAP transporter, C-terminal proteasomal cleavage and epitope recognition parameters, the default thresholds of 0.05, 0.15, and 0.75 were used, respectively^36^. Additionally, another set of epitopes for all HLA class I alleles in the IEDB was identified by the stabilized matrix method (SMM). Epitopes with percentile rank ≤ 2, IC_50_ < 200 nM, and high rank were investigated and considered strong binders^88^. Epitopes predicted for different alleles by NetCTL 1.2 and IEDB servers were selected for further analysis. Based on both servers' outcomes, common epitopes predicted by multiple alleles and favorable for the desired indices were picked for additional analysis.

On the other hand, HTL epitopes with a length of 15 residues were predicted by HLA class II alleles, including human HLA-DR, HLA-DP, and HLA-DQ alleles, using NetMHC II pan 3.2 server^89^. Based on the determined percentiles of 2, 10, and more than 10%, the presented peptides were categorized as strong, moderate, and non-adhesive, respectively^36^. Using the SMM-align method (NetMHCII 1.1) in the server IEDB^90^, another group of HTL epitopes of the same length by 54 HLA class II alleles from the set of HLA-DR, HLA-DQ, and HLA-DP alleles were identified^85^. Epitopes with IC_50_ < 200 nM, percentile rank ≤ 2, and predicted by multiple alleles by both methods as strong binders were considered for further analyses. IEDB predicts the peptide binding to each MHC-II molecule using artificial neural networks (ANN) on a dataset trained with more than 500,000 binding affinity (BA) and eluted ligand mass spectrometry (EL) measurements and provides reliable results^88^. Also, using the IFNepitope^91^ (https://crdd.osdd.net/raghava/ifnepitope/), IL4pred^92^ (https://webs.iiitd.edu.in/raghava/il4pred/design.php) and IL10pred^93^ (https://webs.iiitd.edu.in/raghava/il10pred/predict3.php), HTL epitopes that specifically induce IFN-γ, IL-4 and IL-10 have been evaluated. The IFNepitope server detects with a maximum accuracy of 81.39% of all MHC class II binding overlapping peptides in a protein or antigen capable of inducing IFN-γ from CD4^+^ T-cells through methods such as machine learning strategy, motif-based analysis, and hybrid integrity^94^.

For further analysis, each selected epitope (CTL and HTL) was subjected to a series of selectivity filters (i.e., immunogenicity, antigenicity, allergenicity, and toxicity), which were predicted with the aid of an IEDB class I immunogenicity server, VaxiJen v2.0, AllerTOP 2.0 and ToxinPred, respectively.

### Prediction of LBL epitopes

This study predicted the linear (continuous) B-cell epitopes with default parameters by five servers: ABCPred^95^, BCPreds^96^, BepiPred^97^, ElliPro^98^, and SVMtrip^99^. To improve accuracy, ABCpred (http://www.imtech.res.in/raghava/abcpred/) uses a recurrent neural network to distinguish between epitopes and non-epitopes^95^. BCPreds prediction (http://ailab.ist. psu.edu/bcpred/) is based on kernel methods and the SVM model with an AUC value of 0.758^96^. IEDB Emini is the tool that allows access to the B-cell level in this server^100^. The training dataset for the random forest algorithm, which is used to train the BepiPred-2.0 web server (http://www.cbs.dtu.dk/services/BepiPred/) based on machine learning, includes numerous linear B-cell epitopes from the IEDB database^97^. The ElliPro tool (https://tools.iedb.org/ellipro/) from the IEDB server was also employed to predict linear B-cell epitopes using default parameters. SVMTrip (http://sysbio.unl. edu/SVMTriP/) uses a support vector machine to integrate similarity, tri-peptide affinity, and antigen epitope prediction, and its AUC reaches 0.702^99^.

Here, BepiPred was first used for the primary prediction, and the results from this server were checked and completed using four other servers. A linear B-cell epitope predicted by BepiPred was only if it was predicted by at least one of the other four servers. Predicting the surface accessibility of epitope sequences from structural proteins is also crucial for placing these sequences in the solvent-exposed region of antigens for B-cell fusion^101^, which Emini tool IEDB^100,102^ has investigated. As a result, the non-surface-placed epitopes are also eliminated. Linear B-cell epitopes identified on the surface of structural proteins were selected based on their antigenic, non-allergic, and non-toxic nature.

### Multiple alignment and selection of fully conserved epitopes

Following multiple alignments of the desired structural protein sequences for various strains using ClustalW^103^ supplied by MEGA^104^ and Bioedit software, the modifications made, such as deletions, additions, and substitutions, were identified in each strain^68^. Then, the predicted CTL, HTL, and LBL epitopes were compared with the results of this alignment, and epitopes that remained constant in all strains were selected as fully conserved epitopes for inclusion in the final vaccine construct^68^.

### Population coverage, epitope protection analysis, and autoimmunity identification

The IEDB population coverage analysis tool (https://tools.iedb.org/population/) with default parameters was employed to test the immune response of CTL and HTL epitopes for a vaccine designed against their respective HLA genotype frequencies and adequate coverage of the human population worldwide^105^. On the other hand, the IEDB conservancy analysis tool (http://tools.iedb.org/conservancy) that is part of the IEDB (http://www.immuneepitope.org) was used to help select T and B-cell epitopes with a favorable level of conservation or variability and to assess the degree of conservation of the selected epitopes in comparison to other similar sequences^106^. This server output page displays the degree of epitope conservation from 0 (minimum) to 100% (maximum)^107^. In addition, to avoid inducing host autoimmune diseases and cross-reactions, each selected epitope was further analyzed with BLASTp against the UniProt database to check their homology to the human proteome, and any vaccine epitope with an identity equal to or higher than 35% was considered a homologous protein with the human proteome and removed. Therefore, only the non-homologous epitopes of the human proteome were selected for vaccine construction^108,109^.

### Multi-epitope vaccine design

In this study, an overlapping method was considered to prevent the selection of repetitive epitopes when choosing the appropriate epitopes from the various epitopes obtained for making a vaccine. Epitope types were chosen based on immunogenicity, non-allergenicity, high population coverage, lack of overlap with human proteins, and accessibility. They were then incorporated into the final structure of the multi-epitope vaccine^81^. The final vaccine consists of 566 amino acid residues, which include 6 CTL epitopes, 6 HTL epitopes, 2 cytokine-inducing epitopes (IFN-γ, IL-4, and IL-10), 9 linear B-cell epitopes, PADRE universal epitope, and fynomer sequence (VTLFVALYDYEARTEDDLSFHKGEKFQILNSSEGDWWEARSLTTGETGYIPSNYVAPVDSI). In the structure of this vaccine candidate, CTL epitopes were linked by the AAY (Ala-Ala-Tyr) linkers, the GPGPG (Gly-Pro-Gly-Pro-Gly) linkers linked HTL epitopes, and the LBL epitopes were linked by KK (Lys-Lys) linkers^110,111^. Fynomer was added to the vaccine structure by the GPGPG linker between the T and B-cell epitope regions (Supplementary Fig. S6). PADRE, which has the amino acid sequence AKFVAAWTLKAAA, is a simple carrier epitope. Combined with an adjuvant, it provides an effective response and can be used in making multi-epitope and recombinant vaccines^112^. To activate helper T-cells (CD4^+^ T-cells) and reduce the polymorphism of HLA-DR molecules in the population, this epitope has been added to the vaccine structure with the GPGPG linker. In addition, to enhance and heighten the long-term immune response, the adjuvant 50S ribosomal protein L7/L12 (rpIL) (Locus RL7_MYCTU) (accession number P9WHE3 in NCBI) was added to the N-terminal of the vaccine via the EAAAK linker^113^. Since the Food and Drug Administration (FDA) and the European Medicines Agency (EMA) discourage the 6His tag due to its potential adverse immune response, alternative tags, including humanized versions derived from human BLAST proteins such as H3A (HAAHAH), H5T (HTHTHTHTH), and H5E (HEHEHEHEH), were designed and tested, showing favorable potential in purification and functional properties in human biopharmaceutical production^114^. Finally, for this structure, a tag H5E was put on the C-terminal of the construct to aid in protein purification and identification.

### Evaluation of antigenicity, allergenicity, toxicity, solubility and physicochemical properties

The VaxiJen v2.0 server (http://www.ddg-pharmfac.net/vaxijen/VaxiJen/VaxiJen.html) with a threshold value of 0.4 with viral databases was used to extract prediction models of total protein antigenicity to verify the antigenicity of the vaccine. VaxiJen relies on the automatic cross-covariance (ACC) transformation of protein sequences into uniform vectors with original amino acid features^83^. AntigenPro is another server for this, a protein antigenicity predictor that uses a sequence-based, unaligned, pathogen-independent approach^82^. Scores of more than 0.4 for the VaxiJen viral model and more than 0.8 for ANTIGENpro are considered in this study as indicators of antigenicity. The AllergenFP 1.0 (http://ddg-pharmfac.net/Aller genFP/) and the AllerTOP 2.0 (https://www.ddg-pharmfac.net/AllerTOP/) were utilized to evaluate allergenicity. AllergenFP uses a binary classification to categorize allergens and non-allergens. Both servers use automatic cross-covariance (ACC) transformation to convert strings into uniform vectors after E-descriptors^28,29^ describe the dataset. ToxinPred forecasts the toxicity of the vaccine and each of its epitopes. This server employs the SVM model and dataset with 1805 toxic peptides (≤ 35 residues)^30^ to distinguish between toxic and non-toxic peptides. The Protein-sol (https://protein-sol.manchester.ac.uk) and the SolPro (http://scratch.proteomics.ics.uci.edu) perform solubility checks. In contrast to SolPro, which is an SVM-based tool for predicting the solubility of a protein sequence with an estimated overall accuracy of more than 74% on tenfold cross-validation^31^, Protein-sol takes advantage of existing data on the solubility of *E. coli* proteins in a cell-free expression system^32^. The ExPASy ProtParam server predicted several physicochemical characteristics of the vaccine structure, including molecular weight, theoretical isoelectric point value (pI), charge, extinction coefficient, half-life, instability index, aliphatic index, and GRAVY score^115^ (https://web.expasy.org/protparam/). Additionally, SignalP4.1^116^ (https://www.cbs.dtu.dk/services/SignalP/) and the TMHMM v2.0 server^117^ (https://www.cbs.dtu.dk/services/TMHMM/) examined the vaccine structure for the presence of signal peptides and transmembrane helices, respectively.

### Secondary structure prediction

The secondary structure of the vaccine construct has various characteristics, such as extended strands, alpha helix regions, random coils, and beta strands. These were analyzed using the SOPMA servers^38^ (https://npsa-prabi.ibcp.fr/cgi-bin/npsa_automat.pl?page=/NPSA/npsa_sopma.html) and the PSIPRED v4.0^118^ (http://bioinf.cs.ucl.ac.uk/psipred/). Both servers carry out the prediction process with an accuracy of over 80%. PSIPRED employs two feed-forward neural networks (FFN) to analyze PSI-BLAST (Position-Specific Iterated-BLAST) outputs^118^. After entering the vaccine sequence into SOPMA, the output width was set to 70, and the parameters for the number of conformational states, similarity threshold, and window width were set to 4 (Helix, sheet, turn, and coil), 8, and 17, respectively.

### Modeling, refinement, and validation of 3D structure

Robetta (https://robetta.bakerlab.org/), an online protein structure prediction service that continuously evaluates different aspects of a prediction (coverage, local accuracy, completeness, etc.), modeled the final 3D vaccine structure via a cameo social project^40,119^. Protein folding in this server is based on deep learning, RoseTTAFold, TrRosetta, and an interactive submission interface that allows for custom sequence alignments for homology modeling, constraints, local fragments, and more in a fast and accurate manner^120^.

In the next step, the generated 3D model was refined by GalaxyRefne^121^ (http://galaxy.seoklab.org/cgi-bin/submit.cgi?type=REFINE). Using molecular dynamics simulations, this server first rebuilds and repacks the side chains, followed by general relaxation and structural disruption. According to CASP10 assessments, this method has performed best in improving the local structure quality^122^. GDT-HA, RMSD, Molprobity, clash score, and Ramachandran plot evaluated the quality of the refined model.

Model validation was performed using the ProSA web server^65^ (https://prosa.services.came.sbg.ac.at/prosa.php) and the SAVES v6.0 server (https://saves.mbi ucla.edu). Based on the Z-score, ProSA evaluates and confirms the overall and local quality of the predicted model and calculates an overall quality score for the desired input structure. If the Z-score is outside the specified range for the native protein, the probability of errors in the predicted structure increases. A local quality score plot is shown in the 3D molecular viewer^65^ to make it easier to spot problematic areas of the model. The subsets of ERRAT^123^, VERIFY3D^124^, PROVE^125^, PROCHECK^126^, and WHATCHECK^127^, which are made available to users through SAVES v6.0, can be used to predict the structural quality of the chosen model and the evaluation of various stereochemical parameters of the protein structure. The PROCHECK tool evaluates the anticipated model's stereochemical quality by analyzing the model's overall geometry with residues through the residue geometry^126,128^. To generate the Ramachandran plot, there are several tools and servers available, including the PDBsum server^129^, MolProbity^130^, STAN Server^131^, and RAMPAGE^43^. However, in this study, we used the PDBsum server. Using UCSF ChimeraX software, the final model of the vaccine structure was drawn^132^.

### Prediction of conformational B-cell epitopes

After predicting, refining, and verifying the 3D model of the vaccine, conformational (discontinuous) B-cell epitopes were predicted using the ElliPro server^98^ (http://tools.iedb.org/ellipro). Based on the geometrical features of the protein structure, solvent accessibility, and flexibility, this method predicts B-cell conformational epitopes, which are longer than other epitopes. The minimum score and maximum distance (Angstrom), two prediction parameters, were regarded as 0.8 and 6, respectively. ElliPro is a reliable tool for identifying antibody epitopes and predicting the B-cell conformational epitopes with an AUC score of 0.732^98^.

### Molecular docking and binding affinity analysis

The 3D structures of the TLR2, TLR4, MHC-I, and MHC-II receptors were retrieved from the Protein Data Bank (PDB) (https://www.rcsb.org) with the identifiers 2Z7X, 2Z63, 1I1Y, and 1KG0, respectively. Then, all ligands and water molecules attached to these structures were eliminated. The CPORT^133^ (Consensus Prediction Of Interface Residues in Transient complexes) predicted the active and inactive amino acid residues involved in the protein–protein interactions. These results were then submitted to the HADDOCK 2.4 server (https://wenmr.science.uu.nl/haddock2.4/) for docking^134^ (Supplementary Table S16). HADDOCK (High Ambiguity Driven protein–protein DOCKing) uses Python scripts to compute the structure using crystalline and NMR structures^135^. Based on the lowest HADDOCK score, we selected the best cluster and corrected it with the HADDOCK correction interface. Using the PRODIGY web server, the binding affinity of each docked complex was determined by selecting the best-refined structure from the best cluster. The server offers web services for identifying biological interfaces and predicting affinity in complexes using crystallography^136,137^. The binding affinity of the four docked complexes was analyzed using this server at 37 °C, and its values ​​were represented as Kd. Additionally, ΔG values​​ in kcal mol^−1^ for the complexes were also obtained. Finally, Residues involved in interactions between vaccine and target receptors were visualized with ChimeraX and mapped by PDBsum^138^.

### Energy minimization and molecular dynamics simulation

The GROMACS version 5.1, a Linux-based and open-source program, was employed to conduct molecular dynamic simulation and energy minimization^139^. This program provides various calculation types, preparation, and analysis tools and is supported by several advanced techniques for free energy computing^51^. The vaccine pdb file was employed to initiate molecular dynamics simulations. The topology file generation required for energy minimization and equilibrium was performed under the OPLS (Optimised Potential for Liquid Simulation-All Atom) force field^140^. This structure fits inside an octagonal box of water molecules, represented by the three-point charge model spc216, with the boundary at most minuscule 10 Å away from any protein atom. After determining the net charge of the dissolved protein (vaccine), charged ions were added to the system for neutralization. The LINCS algorithm helped to limit covalent bonds with hydrogen atoms^141^, and long-range electrostatic interactions were treated with Particle Mesh Ewald (PME) using a 10 Å real-space line cutoff^69^. With the main chain atoms constrained to initial coordinates, this system was first minimized under 50,000 steps of steepest descent energy minimization to remove close contacts and spatial overlap. Then, it was put into a two-step NVT (constant number of particles, volume, and temperature) and NPT (constant number of particles, pressure, and temperature) equilibrium phase^142^. The restrained system was gradually heated up to 300 °K (kelvins) under constant volume conditions at 100 ps. The system was then equilibrated for 1 ns using the constant isothermal-isobaric set at 1 atmosphere and 300 °K without any restrictions, which was accomplished by the V-Rescale thermostat and Parrinello-Rahman barostat with an integration time step of 2 fs^143,144^. Using the PME method, electrostatic forces for both NVT and NPT were calculated^145^. In the last step of MD generation, a complete simulation was conducted for 10 ns, and the structure coordinates were recorded every 10 ps. The GROMACS tool was then utilized to analyze the trajectory files. So, The Root Mean Square Deviation (RMSD) for the backbone, The Root Mean Square Fluctuation (RMSF) for the side chain, and the radius of gyration (Rg) were determined to check convergence. The graphs obtained from various analyses were drawn using the Xmgrace design tool^146^.

### Codon optimization and in-silico simulation of vaccine

The Java Codon Adaptation Tool (JCat) (http://www.prodoric.de/JCat) was used for codon optimization and reverse translation. JCat performs codon adaptation for most selected prokaryotic and eukaryotic organisms sequenced^53^. After codon optimization in the *E. coli* strain K12, the vaccine cDNA sequence was created, which can be used for efficient expression in this strain. Further sequence evaluation included selecting parameters to avoid rho-independent transcription terminators, prokaryotic ribosome binding sites, and restriction enzyme cleavage sites. The result includes two measures, guanine-cytosine (GC) content, and codon adaptation index (CAI) score, that assess protein expression levels^147^. Additionally, restriction enzyme sites *Eco53KI* and *EcoRV* were added to the N and C-terminal of the optimized codon sequence for in-silico cloning in the pET-28a ( +) vector using SnapGene 6.2 software^148^ (From Insightful Science, available at https://www.snapgene.com), respectively.

### Simulation of the immune system

Multi-epitope vaccine immunological simulations were carried out on the C-IMMSIM server (https://kraken.iac.rm.cnr.it/C-IMMSIM/) to characterize the immunogenicity of chimeric peptides and real immunogenicity profiles^54^. This server is an immune response agent-based simulator and a flexible tool that uses position-specific scoring matrices (PSSM) and machine learning to predict immune epitopes and interactions. At the same time, it simulates three distinct anatomical regions in mammals, including the thymus, bone marrow, and tertiary lymph organs^149^. To simulate immunity, the vaccine was administered in three injections, each containing 1000 molecules, with a four-week interval between them. The random seed, simulation volume, and simulation step parameters were set to 12,345, 10 μl, and 1050, respectively. According to studies, the minimum interval for two injections should be four weeks. The three injections were followed by time steps of 1, 84, and 168, each representing approximately 8 h of real-life^113^. It is suggested that consecutive vaccine injections be administered at specific time intervals to evaluate the effects of vaccine exposure on repeated SARS-CoV-2 infections.

## Supplementary Information

Supplementary Information.

Supplementary Tables.

## Data Availability

All data generated or analyzed during this study are included in this published article (and its Supplementary Information files).

## Abbreviations

CPORT: Consensus prediction of Interface residues in transient complexes
HADDOCK: High ambiguity driven protein–protein docking
MDS: Molecular dynamics simulation
OPLS: Optimised potential for liquid simulation
PRODIGY: Protein binding energy prediction
Rg: Radius of gyration
RMSD: Root mean square deviation
RMSF: Root mean square fluctuation
rplL: 50S ribosomal protein L7/L12
SH3: Src homology 3
SMM: Stabilized matrix method
VOC: Variant of concern
