# Author Correction: Immunoinformatics design of a structural proteins driven multi-epitope candidate vaccine against different SARS-CoV-2 variants based on fynomer

**DOI:** 10.1038/s41598-024-82829-2

**Published:** 2024-12-17

**Authors:** Javad Sarvmeili, Bahram Baghban Kohnehrouz, Ashraf Gholizadeh, Dariush Shanehbandi, Hamideh Ofoghi

**Affiliations:** 1 Department of Plant Breeding and Biotechnology, University of Tabriz, Tabriz, 51666 Iran; 2 Department of Animal Biology, University of Tabriz, Tabriz, 51666 Iran; 3 Department of Immunology, Tabriz University of Medical Sciences, Tabriz, 51666 Iran; 4 Department of Biotechnology, Iranian Research Organization for Science and Technology, Tehran, 33131 Iran

Correction to: *Scientific Reports* 10.1038/s41598-024-61025-2, published online 04 May 2024

The original version of this Article contained an error in the Methods section, under the subheading ‘Prediction of T-cell epitopes’.

“For the TAP transporter, epitope recognition, and C-terminal proteasomal cleavage parameters, the default thresholds of 0.15, 0.05, and 0.75 were used, respectively^36^.”

now reads:

“For the TAP transporter, C-terminal proteasomal cleavage and epitope recognition parameters, the default thresholds of 0.05, 0.15, and 0.75 were used, respectively^36^.”

The original Article has been corrected.

