# Supplementary material for: Immunoinformatics design of a structural proteins driven multi-epitope candidate vaccine against different SARS-CoV-2 variants based on fynomer
Source: Sci Rep. 2024 May 4;14:10297. doi: 10.1038/s41598-024-61025-2 (PMC11069592; doi:10.1038/s41598-024-61025-2)
Supplement: Supplementary file 1 — Supplementary Information. [file 41598_2024_61025_MOESM1_ESM.docx]

List of Supplementary Figures


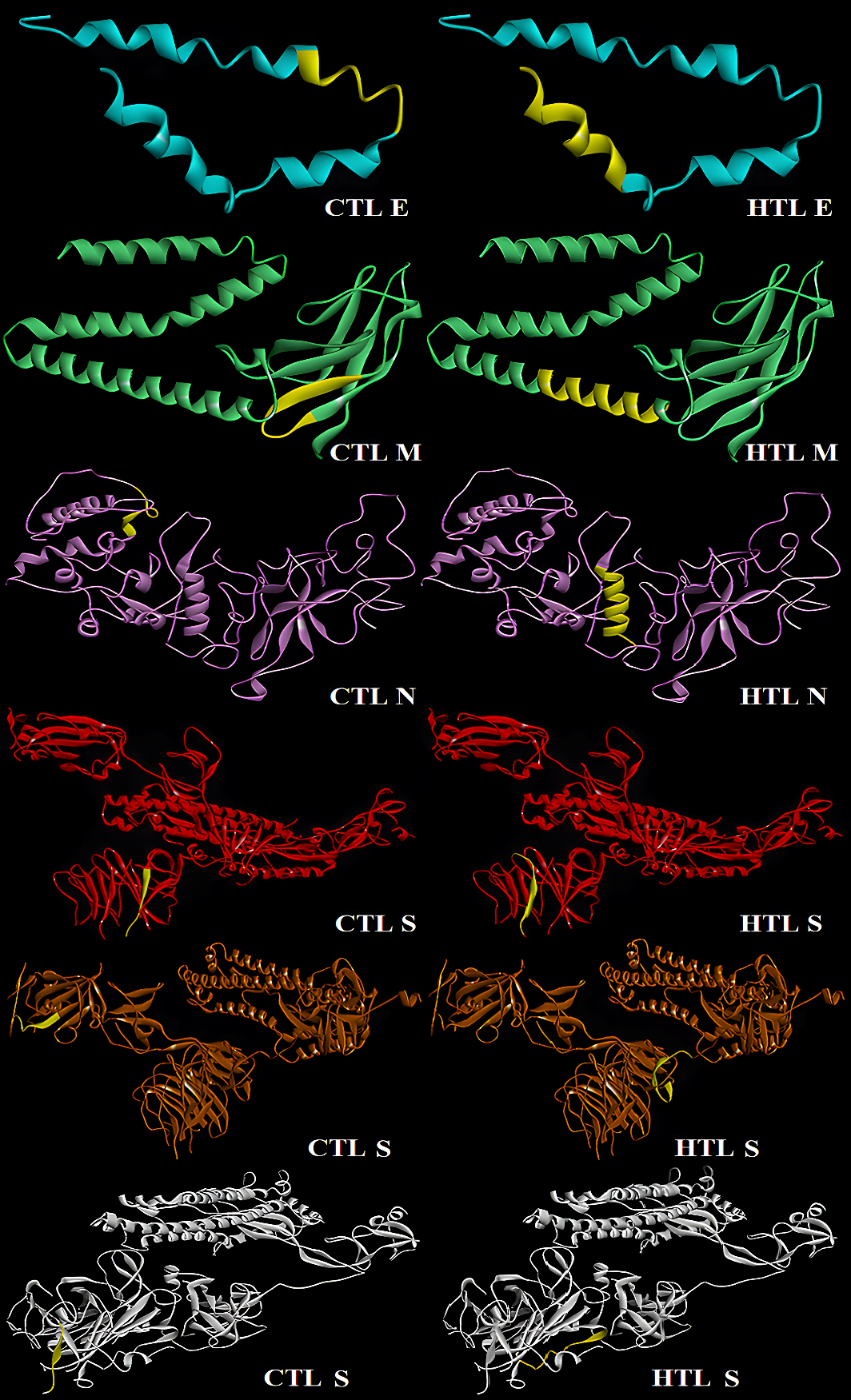


**Supplementary Figure S1.** CTL and HTL epitopes (yellow) are visualized on their respective structural proteins.


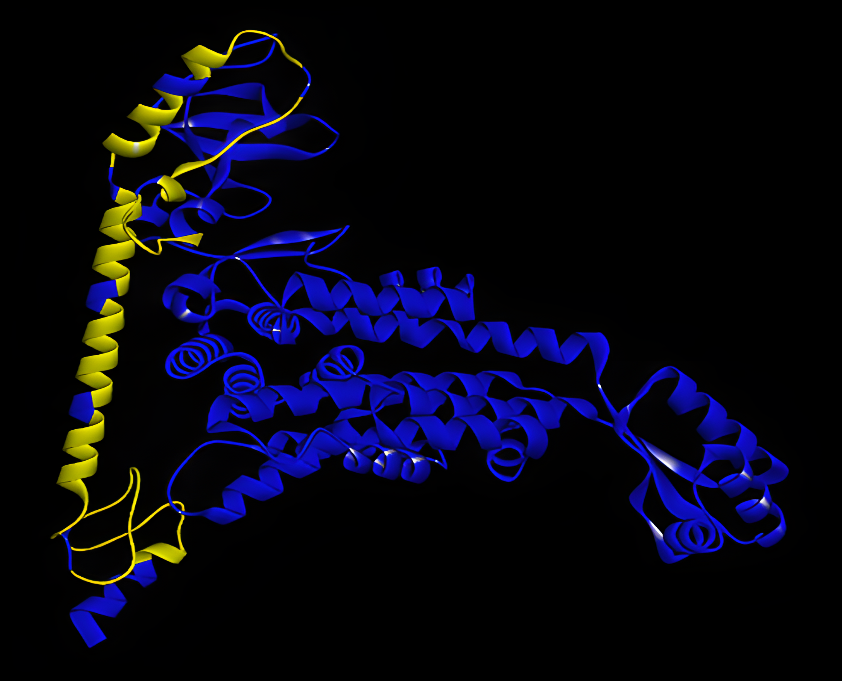


**Supplementary Figure S2.** Continuous B-cell epitopes (yellow) in the construction of the vaccine.


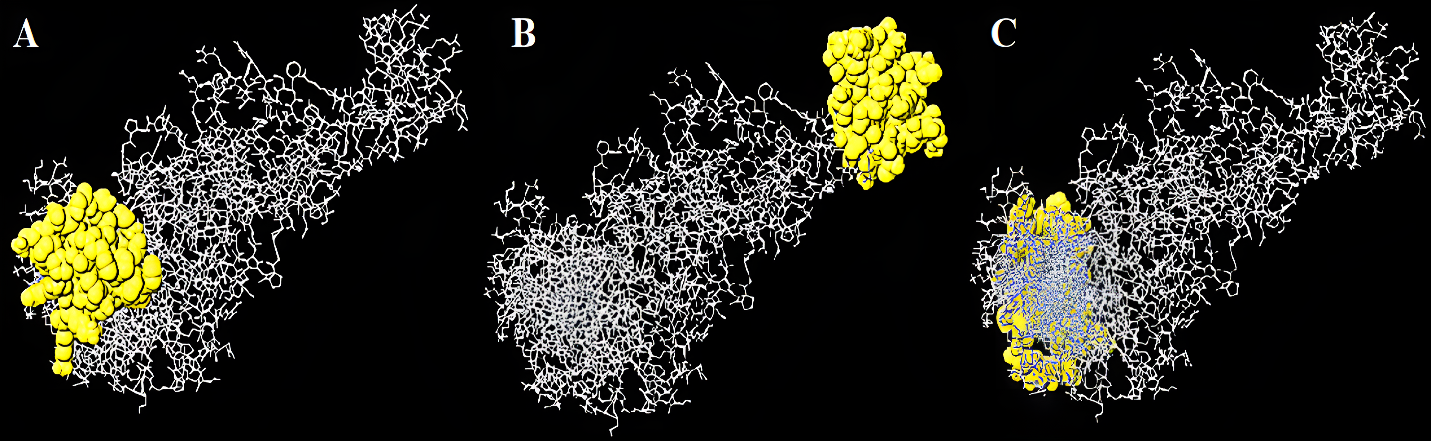


**Supplementary Figure S3.** Predicted discontinuous epitopes on the surface of the designed vaccine. The yellow regions are the conformational B-cell epitopes, while the grey regions are the residues.


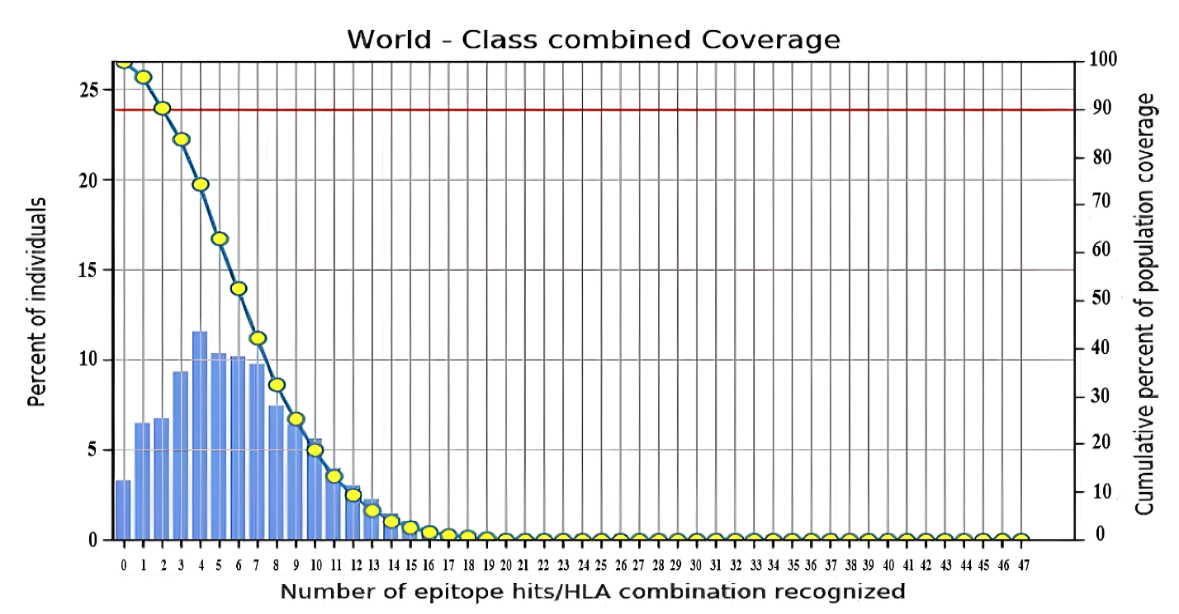


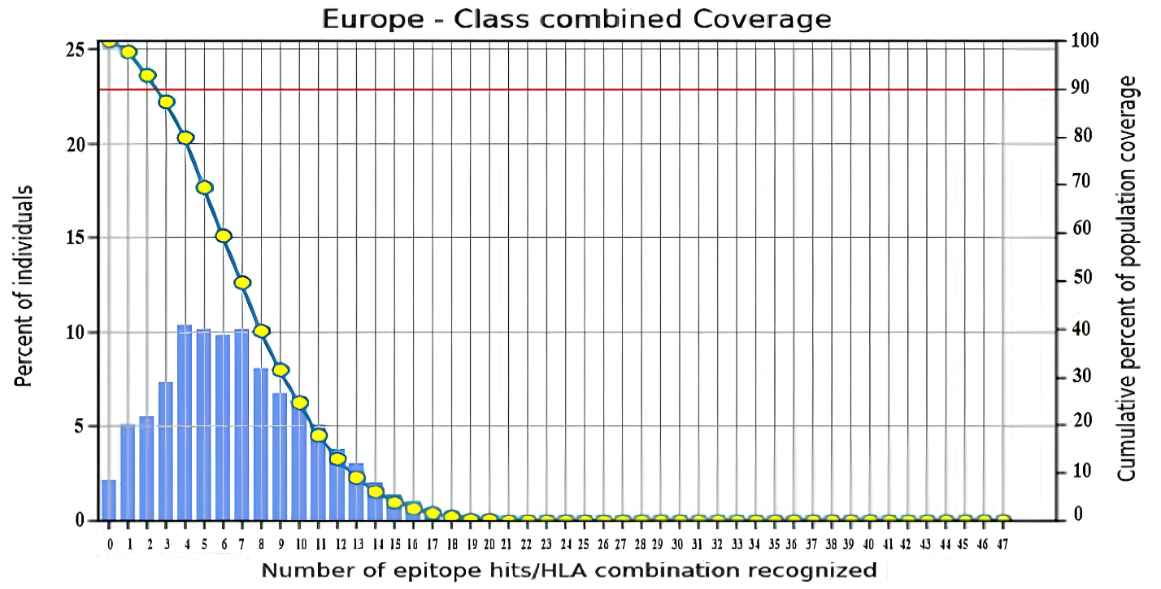

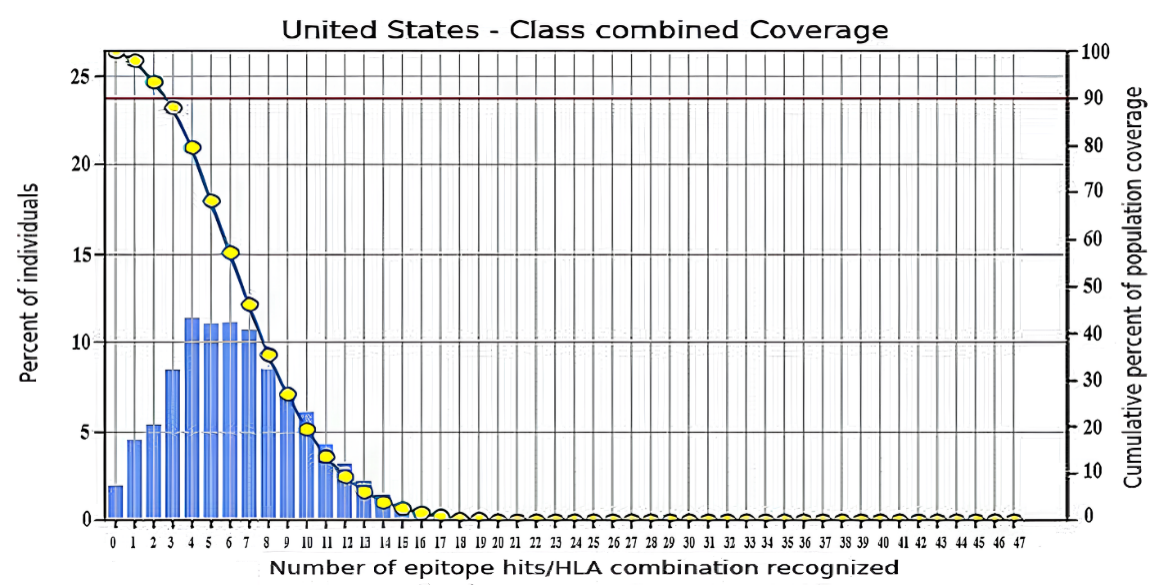


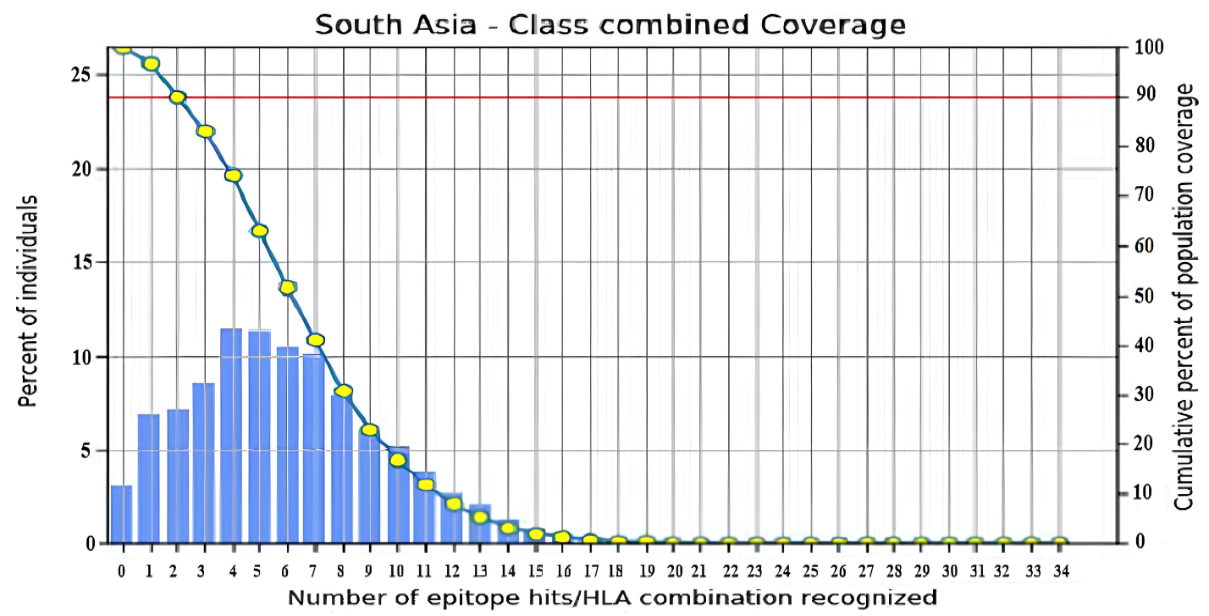


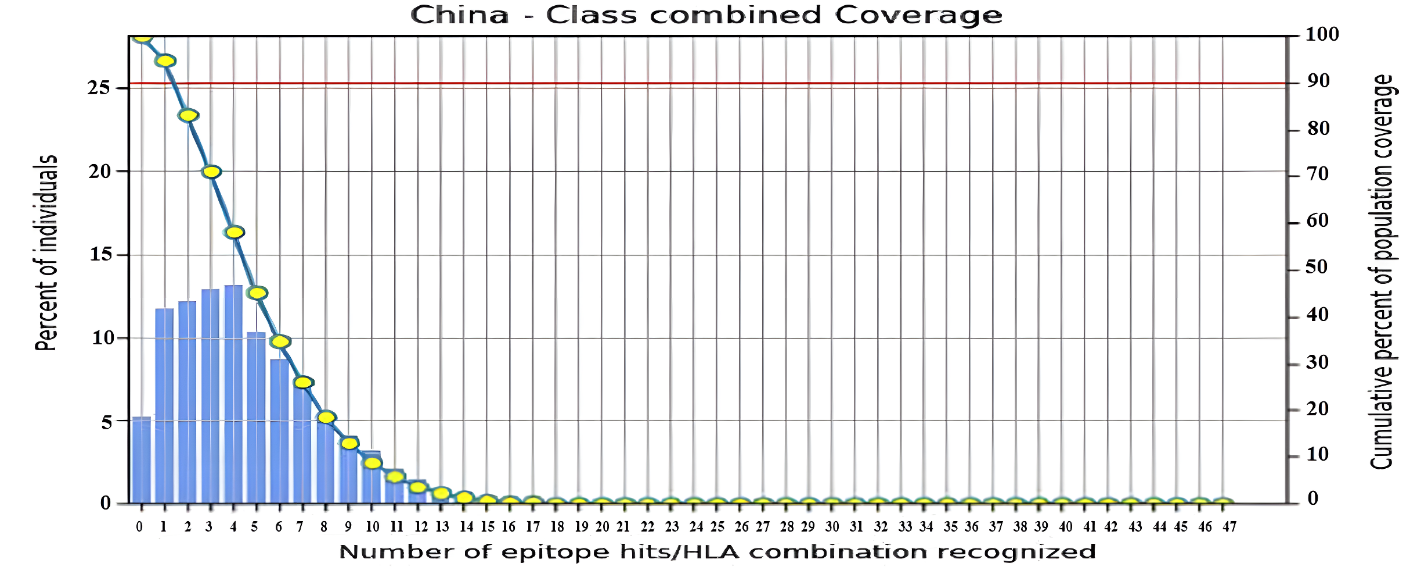


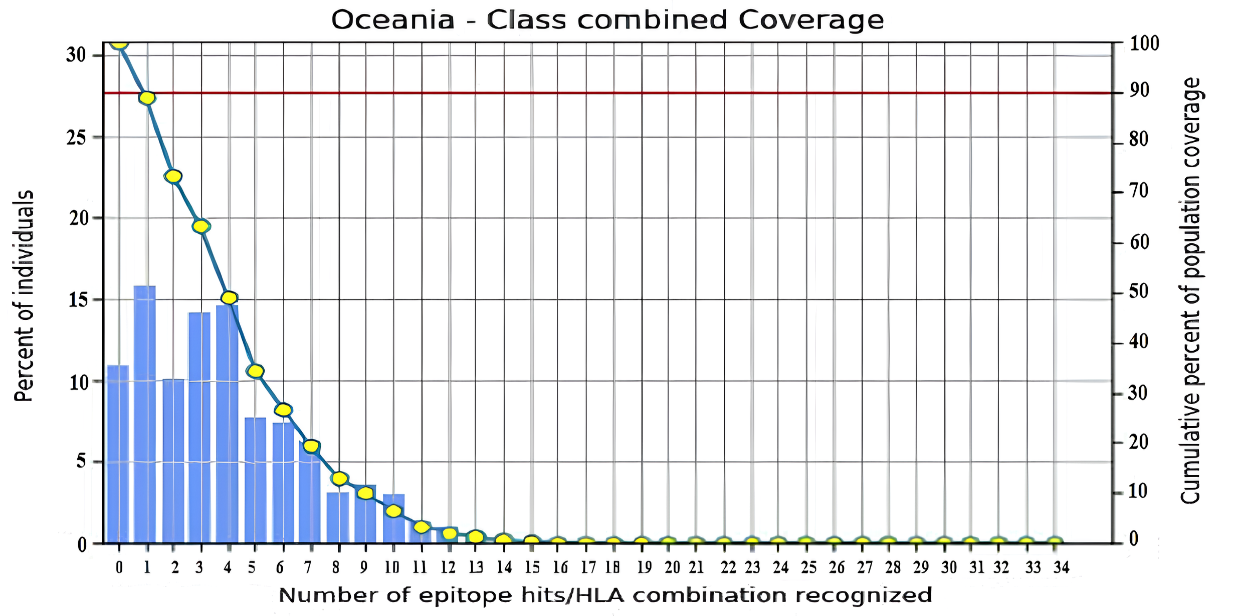


**Supplementary Figure S4.** Plots showing population coverage.

**
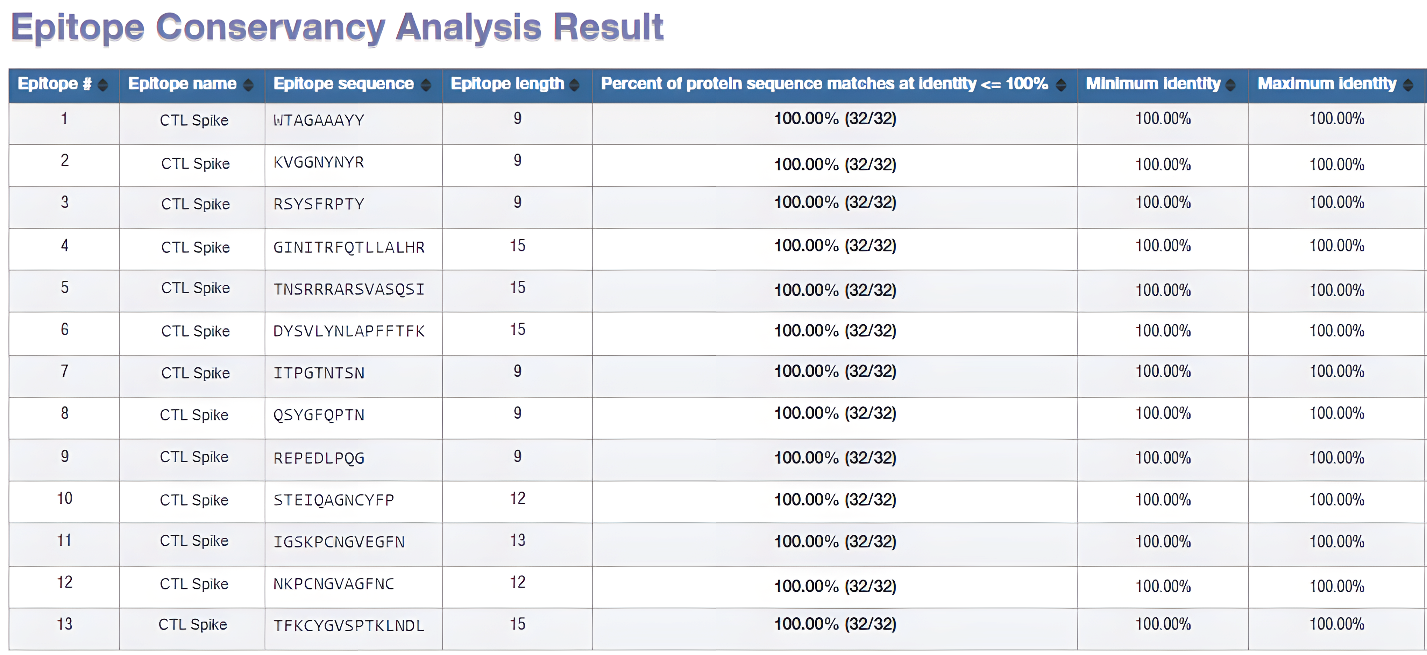
**

**
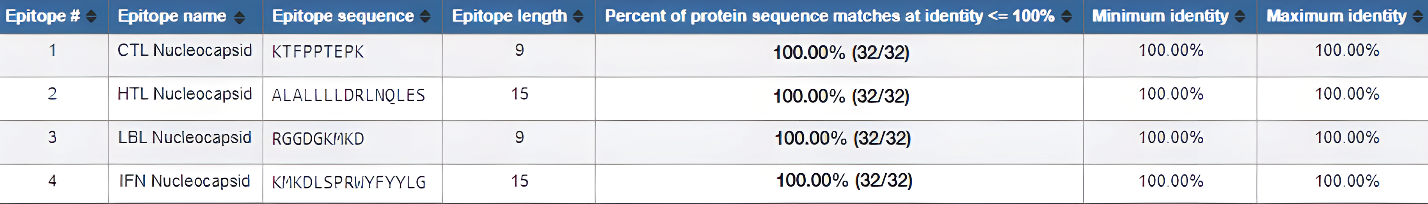
**

**
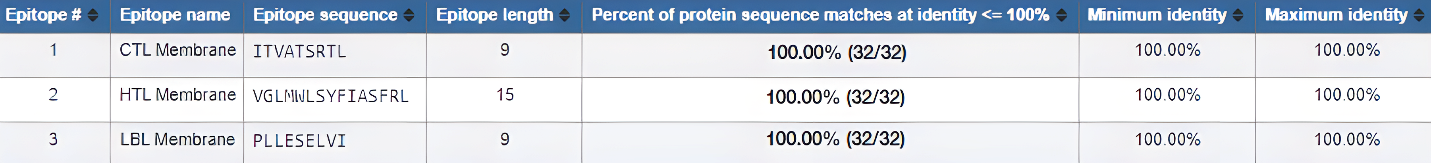
**

**
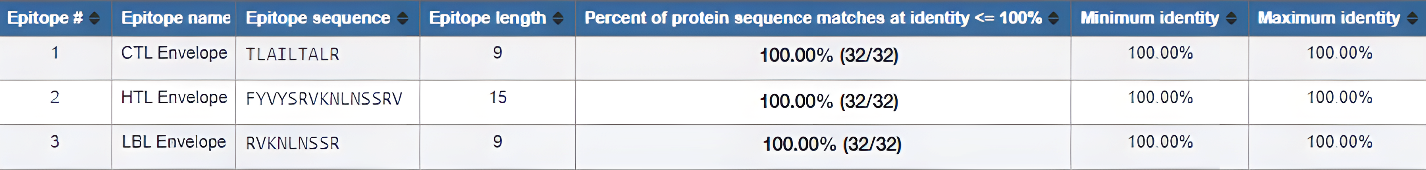
**

**Supplementary Figure S5.** Prediction of the conservation of all T and B-cell epitopes used in the final structure of the vaccine.


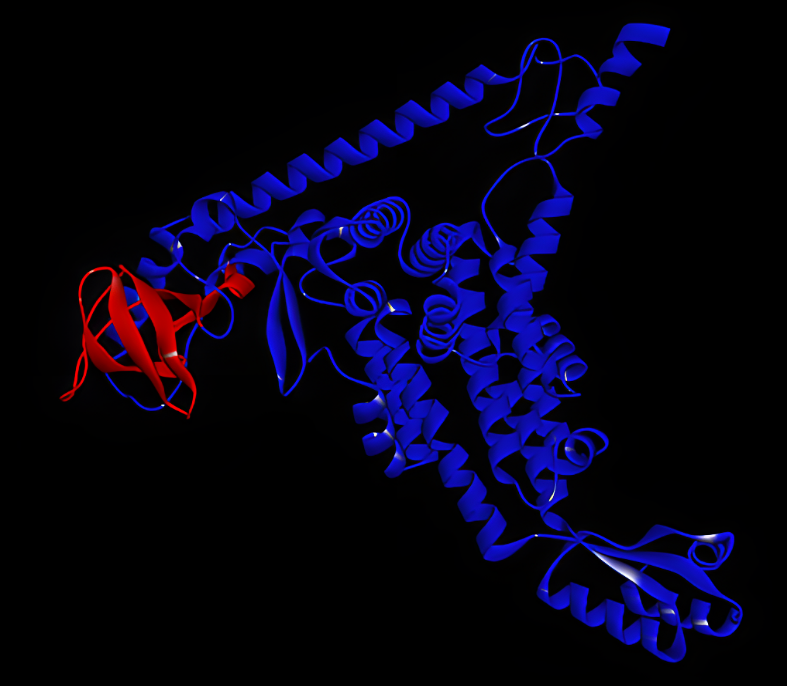


**Supplementary Figure S6.** Fynomer structure (red) in the structure of the designed vaccine.


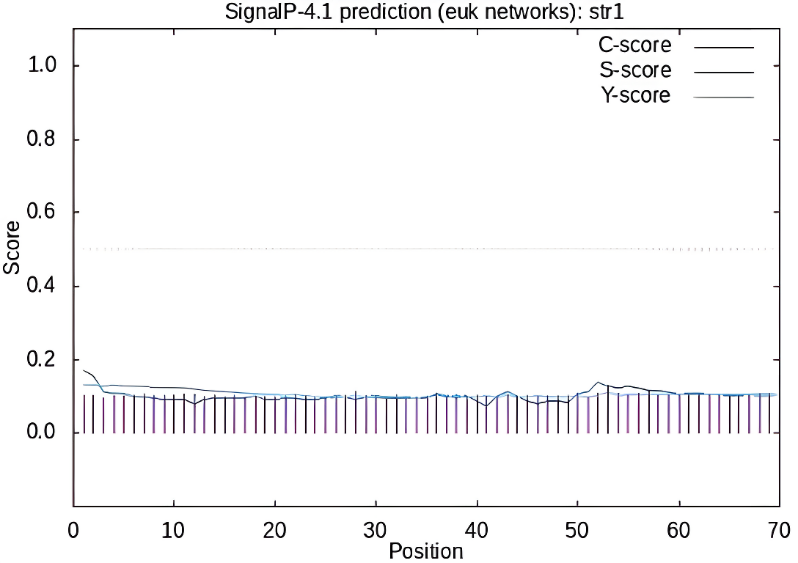


**Supplementary Figure S7.** Signal Peptide.

Measure Position Value

max. C 19 0.107

max. Y 70 0.106

max. S 60 0.111

mean S 1-69 0.097

D 1-69 0.101 0.450 NO

Name=Sequence SP='NO' D=0.101 D-cutoff=0.450 Networks=SignalP-noTM


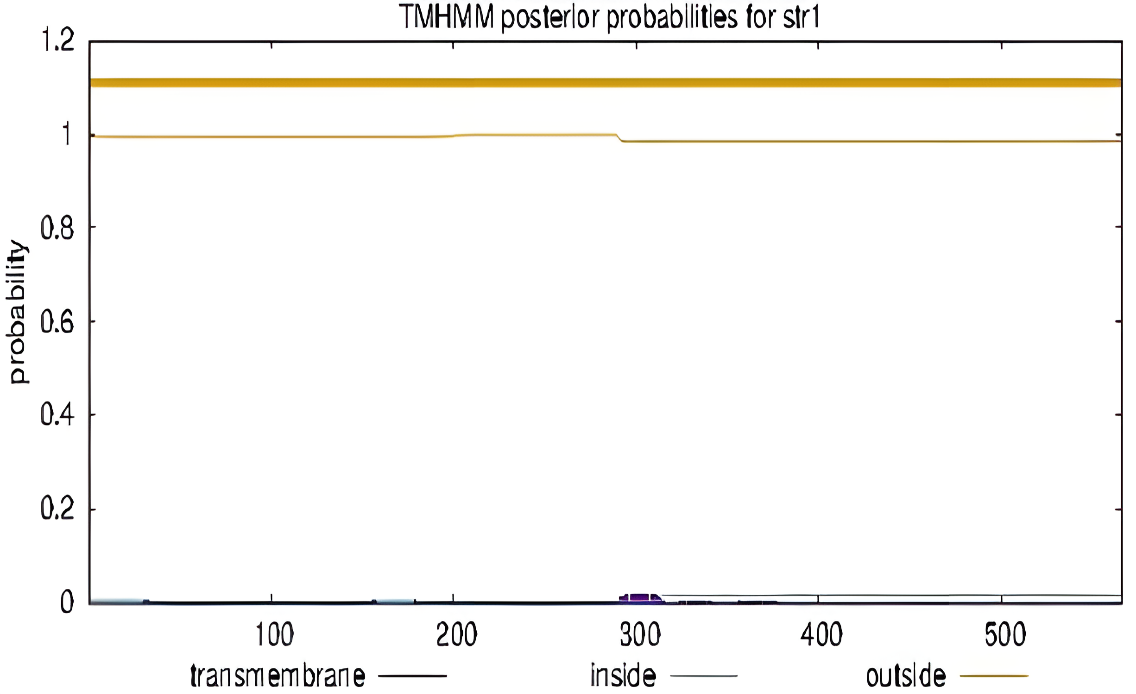


**Supplementary Figure S8.** Transmembrane Helix (TMHMM result).

- Length: 566
- Number of predicted TMHs: 0
- Exp number of AAs in TMHs: 0.52945
- Exp number, first 60 AAs: 0.04915
- Total prob of N-in: 0.00595
- TMHMM2.0 outside 1 566

**
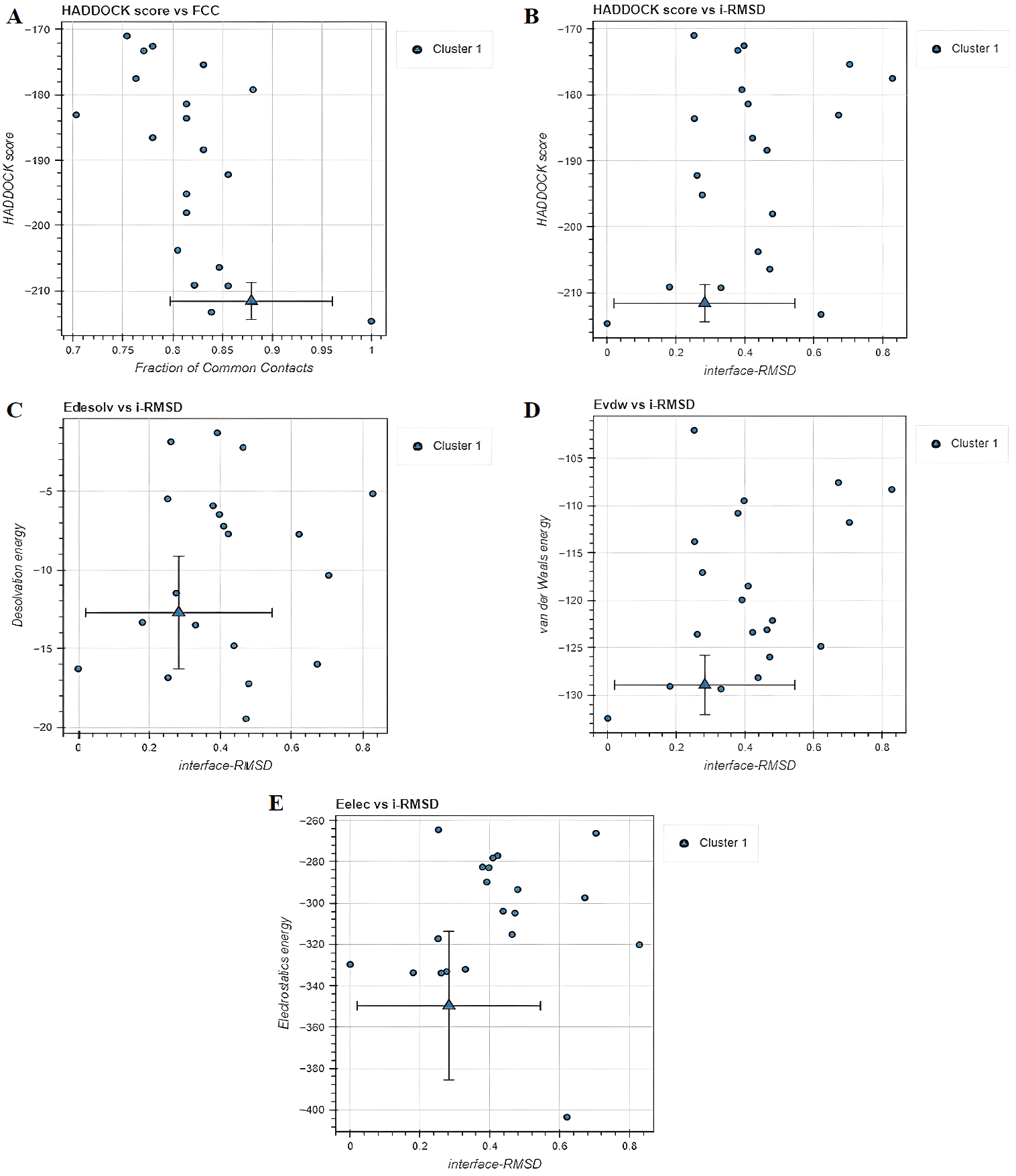
**

**Supplementary Figure S9.** Results analysis related to the HADDOCKH run of the Vac-TLR2 complex. (**A**) HADDOCK score vs FFC (Fraction of shared contacts). (**B**) HADDOCK score vs i-RMSD (interface-RMSD). (**C**) Edesolv (Desolvation energy) vs i-RMSD. (**D**) Evdw (van der Waals energy) vs i-RMSD. (**E**) Eelec (Electrostatics energy) vs i-RMSD.

**Explain:** The results and graphics presented below are based on water-refined models obtained by the HADDOCK server. The cluster is calculated based on the interface ligand RMSDs calculated by HADDOCK, and the interface is automatically defined based on all observed contacts. Colored dots with associated error bars indicate the cluster averages and standard deviations. Different structural analyses, i.e., FCC, i-RMSD, Edesolv, Evdw, Eelec, and Eair, have been made according to the clusters obtained from HADDOCK. The average values ​​are calculated on the top 4 structures of each cluster based on the HADDOCK score.

i-RMSD: interface‌-RMSD on the backbone, including P, O, N, C, and CA atoms, was calculated for all residues involved in intermolecular interaction using a 10 Å cutoff.

FCC: Fraction of shared contacts. The intermolecular contacts are defined based on the best HADDOCK model using a cutoff of 5 Å.

a.u: Arbitrary Units; HADDOCK score and FFC units are arbitrary.

Edesolv: Desolvation energy.

Evdw: van der Waals energy.

Eelec: Electrostatics energy.

Eair: Restraints energy.

**
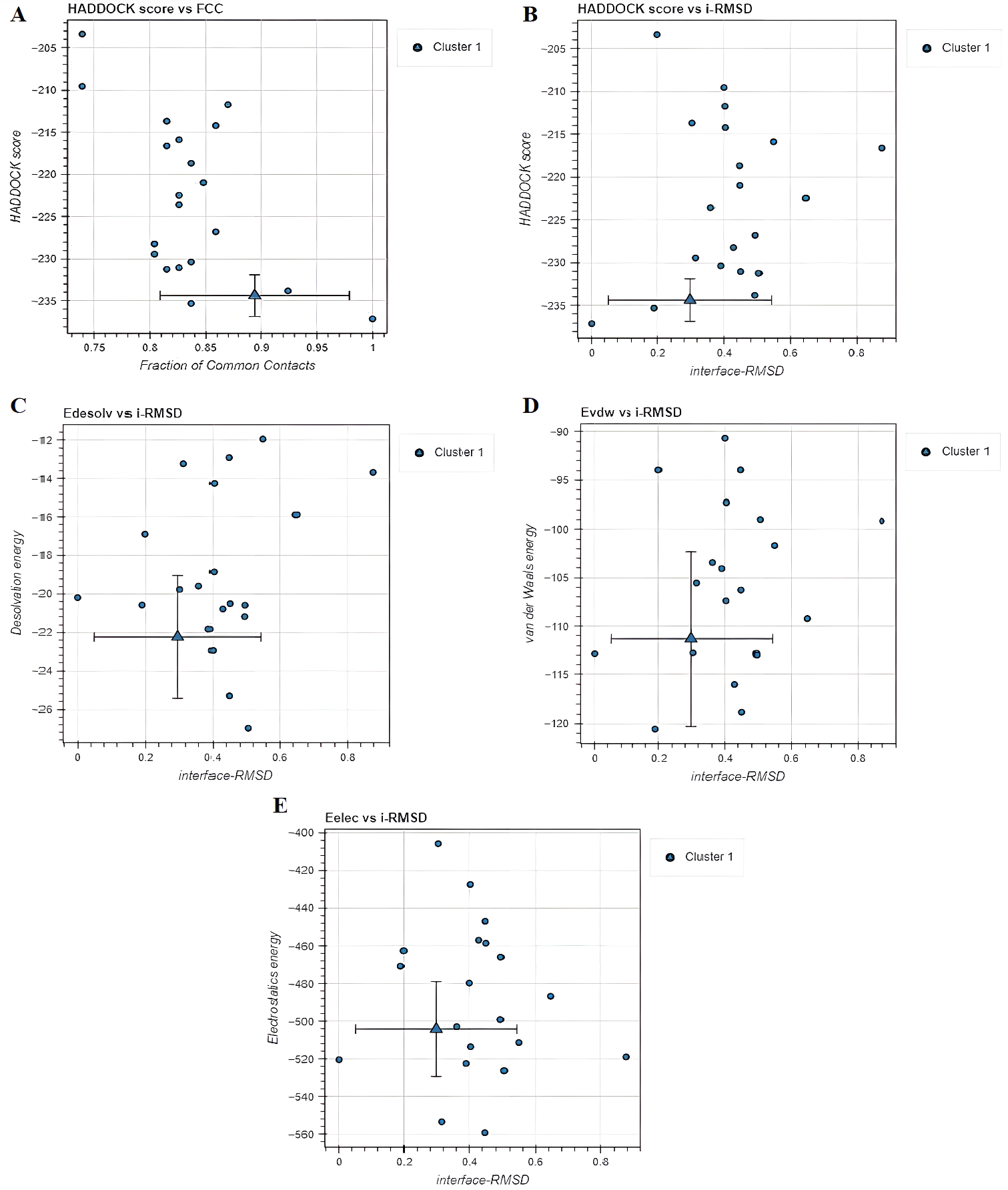
**

**Supplementary Figure S10.** Results analysis related to the HADDOCKH run of the Vac-TLR4 complex. (**A**) HADDOCK score vs FFC (Fraction of shared contacts). (**B**) HADDOCK score vs i-RMSD (interface-RMSD). (**C**) Edesolv (Desolvation energy) vs i-RMSD. (**D**) Evdw (van der Waals energy) vs i-RMSD. (**E**) Eelec (Electrostatics energy) vs i-RMSD.

**
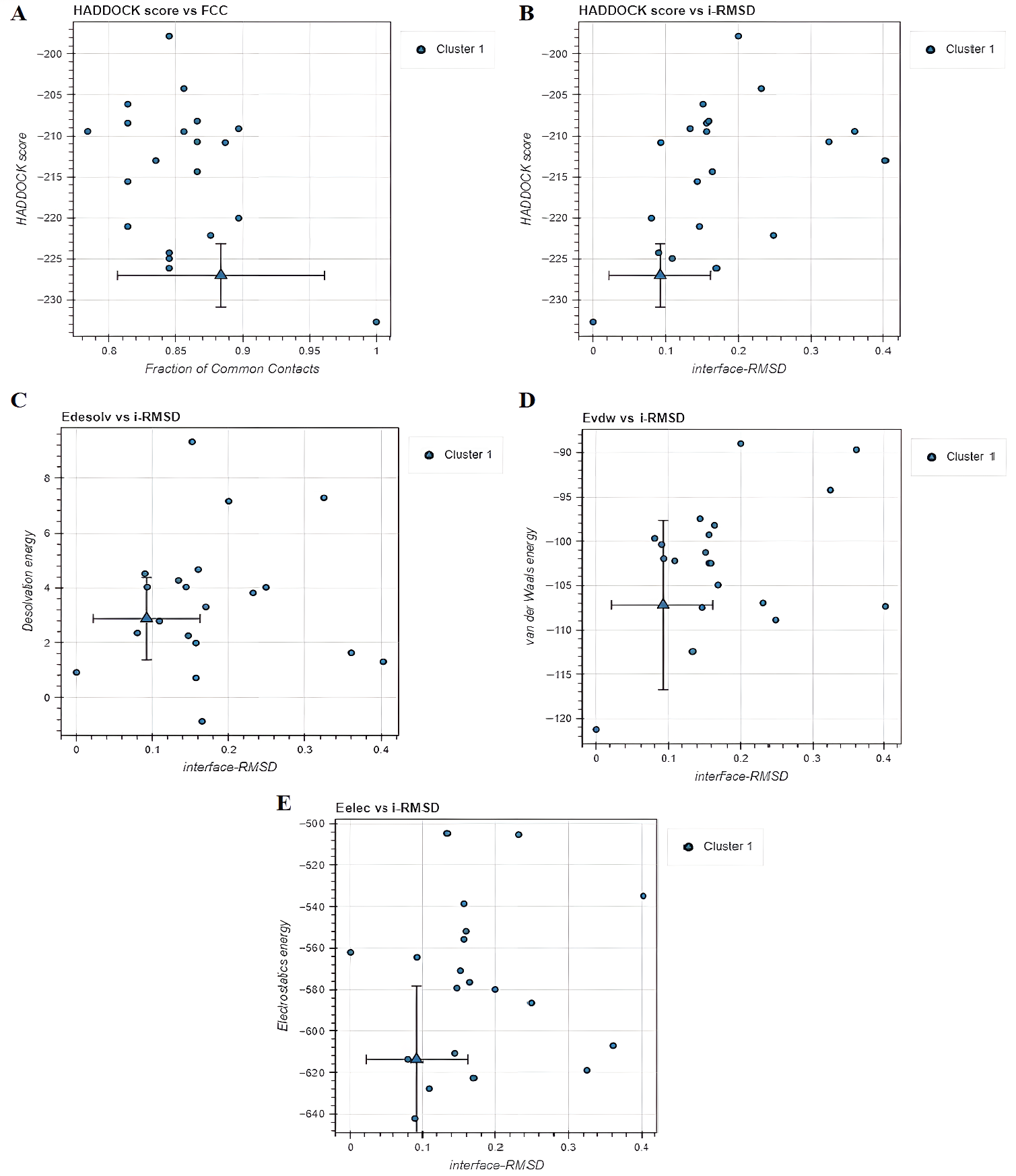
**

**Supplementary Figure S11.** Results analysis related to the HADDOCKH run of the Vac-MHC-I complex. (**A**) HADDOCK score vs FFC (Fraction of shared contacts). (**B**) HADDOCK score vs i-RMSD (interface-RMSD). (**C**) Edesolv (Desolvation energy) vs i-RMSD. (**D**) Evdw (van der Waals energy) vs i-RMSD. (**E**) Eelec (Electrostatics energy) vs i-RMSD.

**
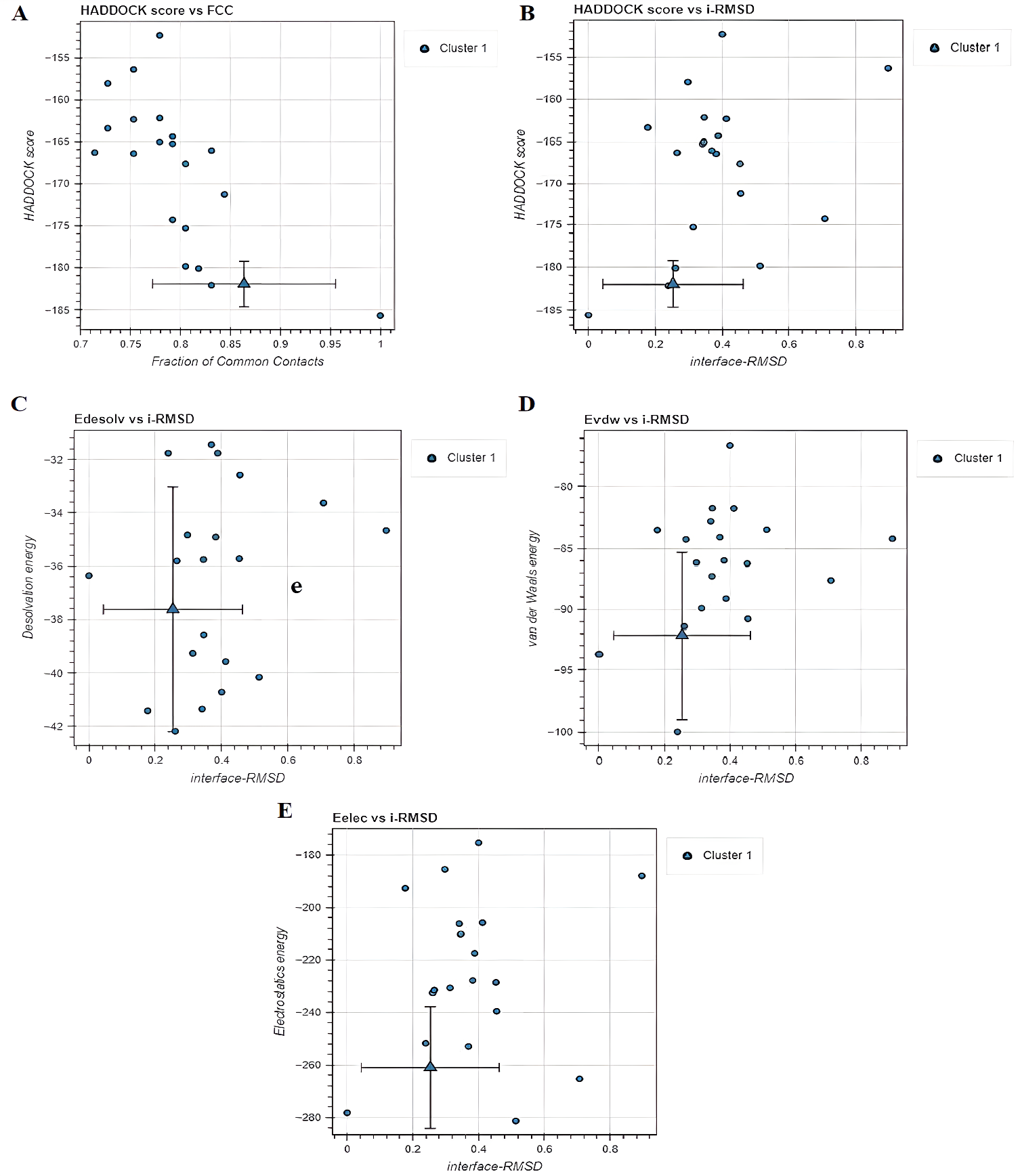
**

**Supplementary Figure S12.** Results analysis related to the HADDOCKH run of the Vac-MHC-II complex. (**A**) HADDOCK score vs FFC (Fraction of shared contacts). (**B**) HADDOCK score vs i-RMSD (interface-RMSD). (**C**) Edesolv (Desolvation energy) vs i-RMSD. (**D**) Evdw (van der Waals energy) vs i-RMSD. (**E**) Eelec (Electrostatics energy) vs i-RMSD.


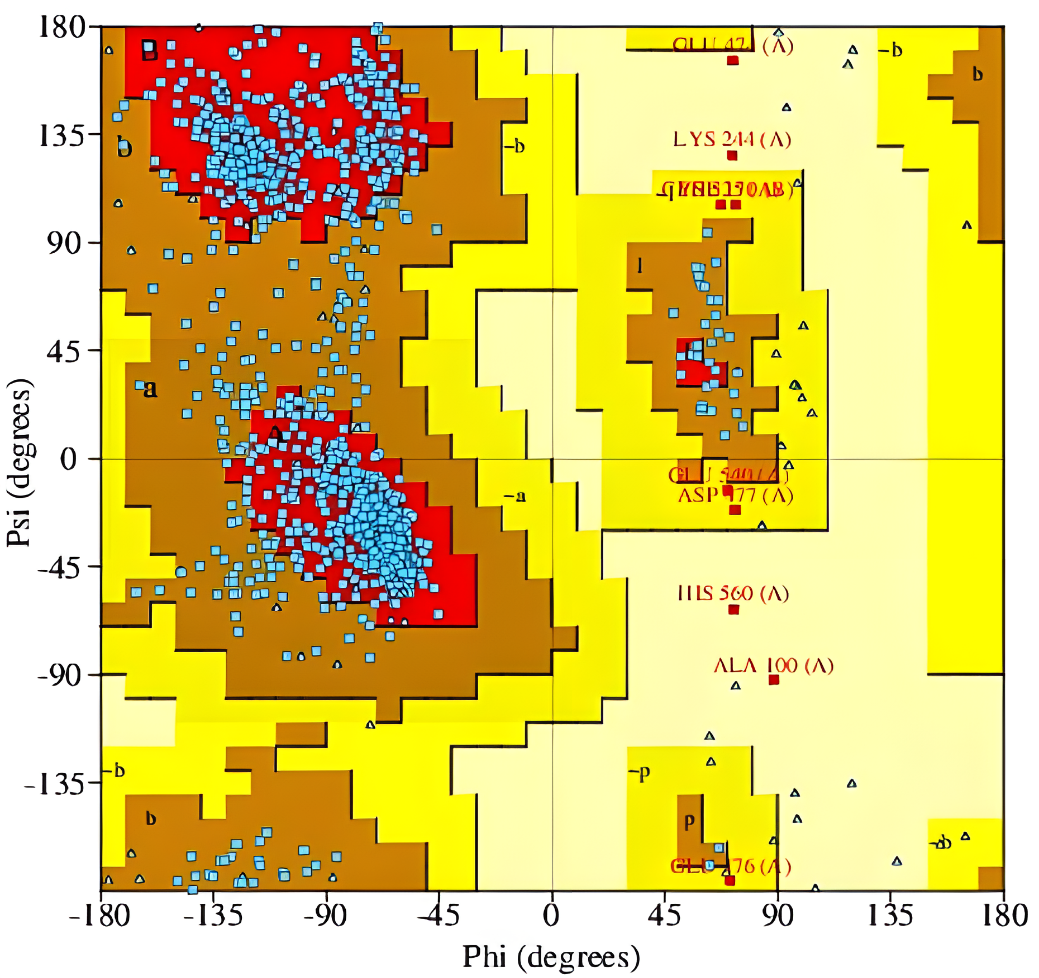


**Supplementary Figure S13.** Ramachandran plot analysis of vaccine-TLR2 docked complex.

Residues in most favored regions [A, B, L] 762 78.6%

Residues in additional allowed regions [a, b, l, p] 199 20.5%

Residues in generously allowed regions [~a, ~b, ~l, ~p] 5 0.5%

Residues in disallowed regions 4 0.4%

Number of non-glycine and non-proline residues 970 100.0%

Number of end-residues (excl. Gly and Pro) 3

Number of glycine residues (shown as triangles) 82

Number of proline residues 60

Total number of residues 1115


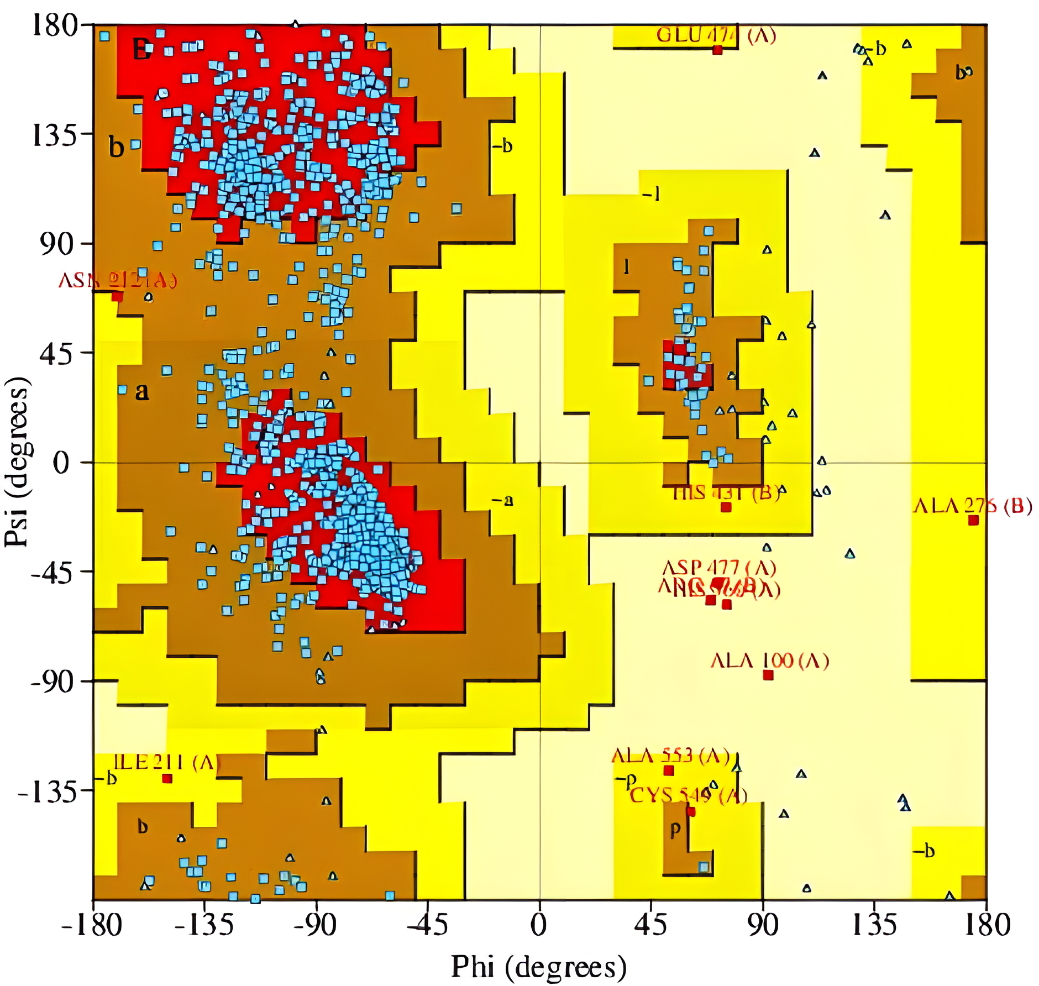


**Supplementary Figure S14.** Ramachandran plot analysis of vaccine-TLR4 docked complex.

Residues in most favored regions [A, B, L] 776 79.0%

Residues in additional allowed regions [a, b, l, p] 195 19.9%

Residues in generously allowed regions [~a, ~b, ~l, ~p] 6 0.6%

Residues in disallowed regions 5 0.5%

Number of non-glycine and non-proline residues 982 100.0%

Number of end-residues (excl. Gly and Pro) 3

Number of glycine residues (shown as triangles) 89

Number of proline residues 62

Total number of residues 1136


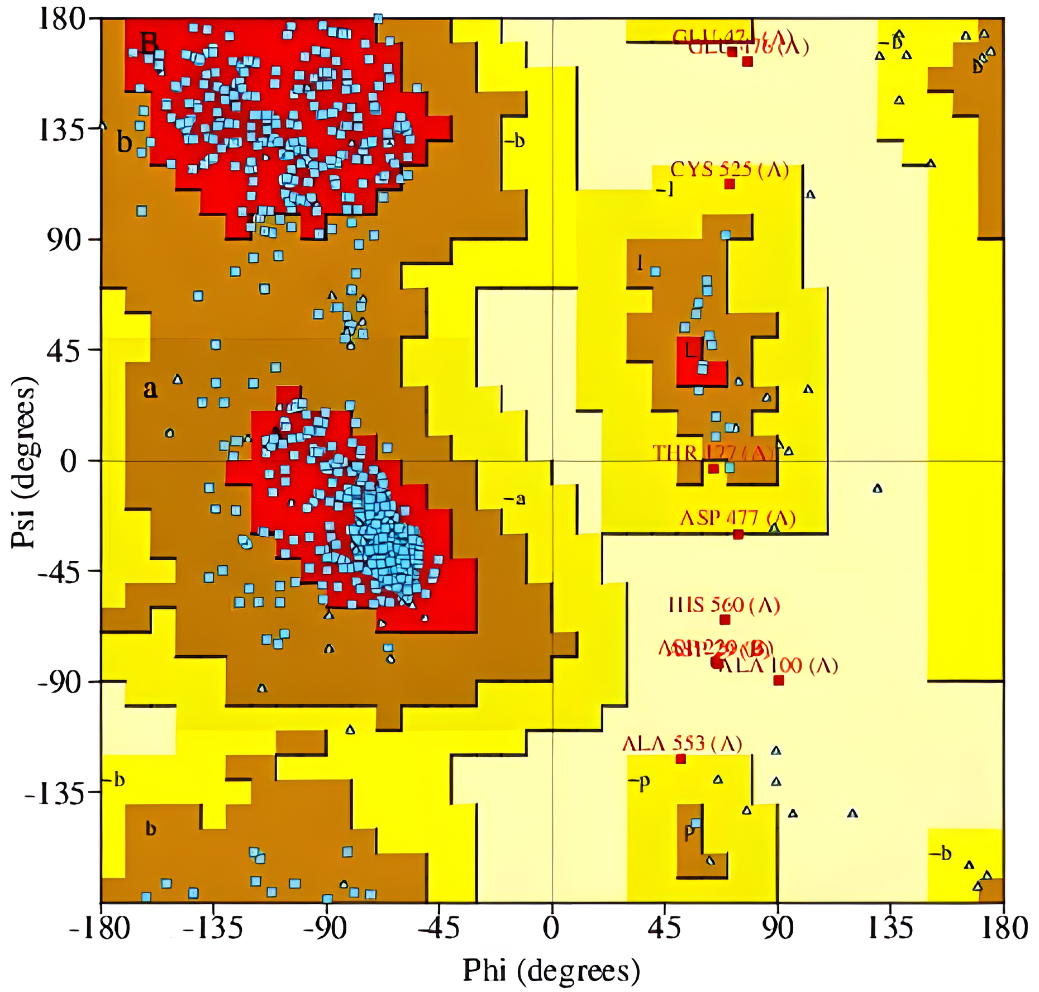


**Supplementary Figure S15.** Ramachandran plot analysis of vaccine-MHC-I docked complex.

Residues in most favored regions [A, B, L] 608 86.2%

Residues in additional allowed regions [a, b, l, p] 87 12.3%

Residues in generously allowed regions [~a, ~b, ~l, ~p] 3 0.4%

Residues in disallowed regions 7 1.0%

Number of non-glycine and non-proline residues 705 100.0%

Number of end-residues (excl. Gly and Pro) 3

Number of glycine residues (shown as triangles) 81

Number of proline residues 52

Total number of residues 841

**
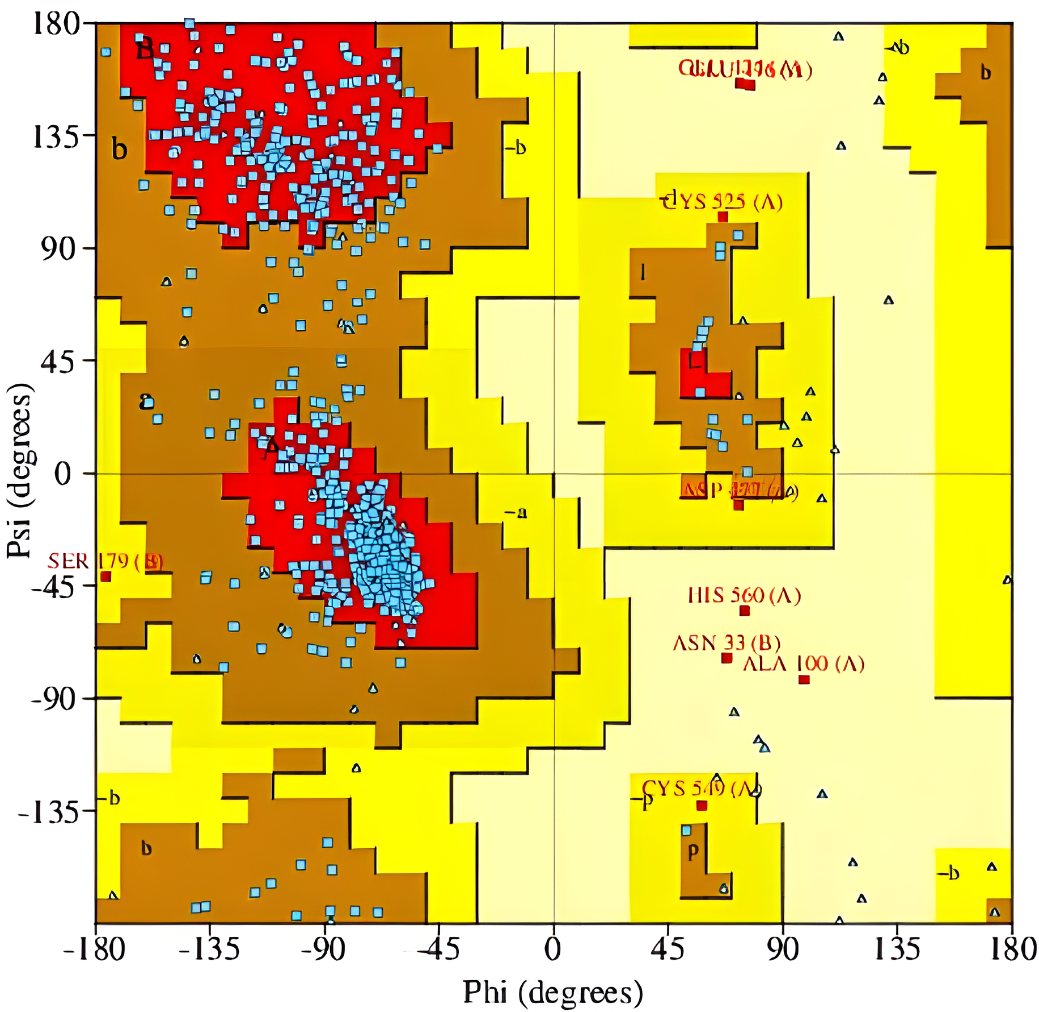
**

**Supplementary Figure S16.** Ramachandran plot analysis of vaccine-MHC-II docked complex.

Residues in most favored regions [A, B, L] 533 85.0%

Residues in additional allowed regions [a, b, l, p] 85 13.6%

Residues in generously allowed regions [~a, ~b, ~l, ~p] 4 0.6%

Residues in disallowed regions 5 0.8%

Number of non-glycine and non-proline residues 627 100.0%

Number of end-residues (excl. Gly and Pro) 4

Number of glycine residues (shown as triangles) 73

Number of proline residues 50

Total number of residues 754

**
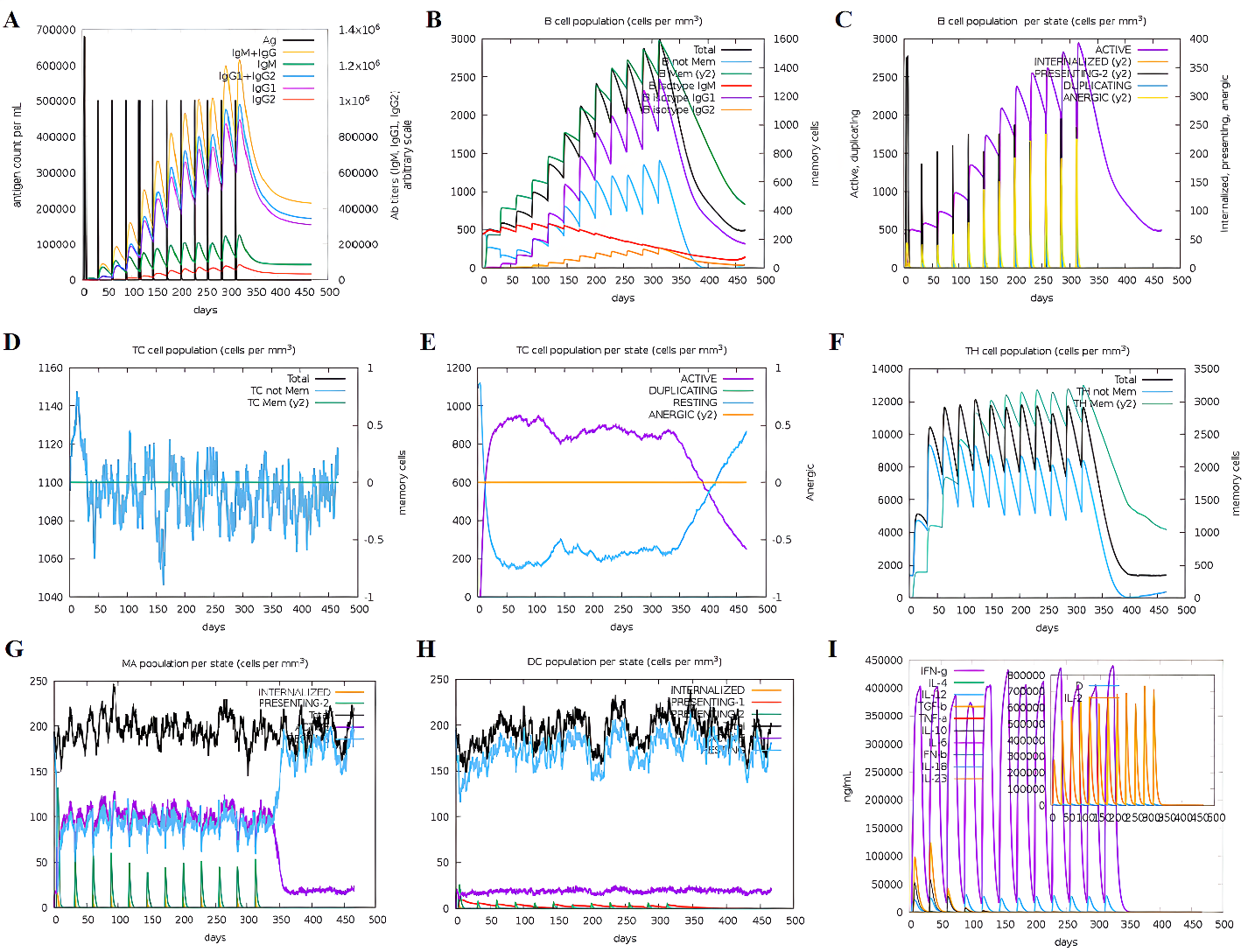
**

**Supplementary Figure S17.** In silico simulation of immune response triggered by the designed vaccine as an antigen after 12 subsequent injections. (**A**) Antigen and subtypes of immunoglobulin levels are represented as different colored peaks. The immunoglobulin (IgG) production represents the proliferation of primary, secondary, and tertiary immune responses after the vaccine administration. (**B**) Moreover, (**C**) B lymphocytes by total count and population per entity state (active, presenting, internalized, duplicating, or anergic). (**D**) Cytotoxic T-cell population. (**E**) Cytotoxic T-cell population per state. (F) Helper T-cell population. (**G**) Macrophages population per state. (**H**) Dendritic cell population per state. (**I**) The concentration of cytokines and interleukins is at three different stages.

**Supplementary Materials**

**Supplementary Material SM1:** Physico-chemical property of the candidate vaccine.

**Number of amino acids:** 566

**Molecular weight:** 60773.29

**Theoretical pI:** 9.36

**Amino acid composition:**

Ala (A) 67 11.8%

Arg (R) 23 4.1%

Asn (N) 22 3.9%

Asp (D) 20 3.5%

Cys (C) 5 0.9%

Gln (Q) 9 1.6%

Glu (E) 36 6.4%

Gly (G) 61 10.8%

His (H) 7 1.2%

Ile (I) 17 3.0%

Leu (L) 48 8.5%

Lys (K) 52 9.2%

Met (M) 5 0.9%

Phe (F) 24 4.2%

Pro (P) 41 7.2%

Ser (S) 33 5.8%

Thr (T) 32 5.7%

Trp (W) 6 1.1%

Tyr (Y) 26 4.6%

Val (V) 32 5.7%

Pyl (O) 0 0.0%

Sec (U) 0 0.0%

(B) 0 0.0%

(Z) 0 0.0%

(X) 0 0.0%

Total number of negatively charged residues (Asp + Glu): 56

Total number of positively charged residues (Arg + Lys): 75

**Atomic composition:**

Carbon C 2746

Hydrogen H 4284

Nitrogen N 738

Oxygen O 801

Sulfur S 10

**Formula:** C_2746_H_4284_N_738_O_801_S_10_

**Total number of atoms:** 8579

**Extinction coefficients:**

Extinction coefficients are in units of M^-1^ cm^-1^, at 280 nm measured in water.

Ext. coefficient 71990

Abs 0.1% (=1 g/l) 1.185, assuming all pairs of Cys residues form cystines

Ext. coefficient 71740

Abs 0.1% (=1 g/l) 1.180, assuming all Cys residues are reduced

**Estimated half-life:**

The N-terminal of the sequence considered is M (Met).

The estimated half-life is: 30 hours (mammalian reticulocytes, in vitro).

>20 hours (yeast, in vivo).

>10 hours (Escherichia coli, in vivo).

**Instability index:**

The instability index (II) is computed to be 34.09

This classifies the protein as stable.

**Aliphatic index:** 73.02

**Grand average of hydropathicity (GRAVY):** -0.368

**Supplementary Material SM2.** Vaccine (Chain A)‌-TLR2 (Chain B) PDBsum interacting molecules.

Hydrogen bonds

<----- A T O M 1 -----> <----- A T O M 2 ----->

Atom Atom Res Res Atom Atom Res Res

no. name name no. Chain no. name name no. Chain Distance

1. 1237 OH TYR 143 A <--> 10590 O SER 571 B 2.71

2. 1333 OH TYR 153 A <--> 10511 SG CYS 562 B 2.98

3. 1342 OD1 ASN 154 A <--> 10545 NZ LYS 567 B 2.92

4. 1374 NH2 ARG 156 A <--> 10242 OD1 ASP 536 B 2.72

5. 1374 NH2 ARG 156 A <--> 10250 O CYS 537 B 3.04

6. 1414 NH1 ARG 160 A <--> 10615 SG CYS 574 B 3.03

7. 1426 OG SER 161 A <--> 10600 N ILE 573 B 2.93

8. 1426 OG SER 161 A <--> 10608 O ILE 573 B 2.77

9. 1885 O ILE 211 A <--> 10533 N GLY 566 B 2.79

10. 1897 N ILE 213 A <--> 10537 O GLY 566 B 3.25

11. 1910 OG1 THR 214 A <--> 10499 NZ LYS 561 B 2.97

12. 1910 OG1 THR 214 A <--> 10537 O GLY 566 B 2.94

13. 2586 O GLY 287 A <--> 5260 N SER 27 B 2.85

14. 4999 OE1 GLU 540 A <--> 5586 NH2 ARG 63 B 2.67

15. 5019 O PHE 542 A <--> 5558 ND2 ASN 61 B 3.02

16. 5038 NZ LYS 544 A <--> 5813 O ASN 86 B 2.83

17. 5051 NZ LYS 545 A <--> 6518 OD2 ASP 160 B 2.69

18. 5075 NZ LYS 547 A <--> 6020 OH TYR 109 B 2.89

Non-bonded contacts

<----- A T O M 1 -----> <----- A T O M 2 ----->

Atom Atom Res Res Atom Atom Res Res

no. name name no. Chain no. name name no. Chain Distance

1. 1236 CZ TYR 143 A <--> 10590 O SER 571 B 3.67

2. 1237 OH TYR 143 A <--> 10589 C SER 571 B 3.54

3. 1237 OH TYR 143 A <--> 10590 O SER 571 B 2.71

4. 1309 CA GLY 151 A <--> 10529 OG SER 565 B 3.64

5. 1326 CB TYR 153 A <--> 10522 CA GLY 564 B 3.58

6. 1326 CB TYR 153 A <--> 10523 C GLY 564 B 3.73

7. 1326 CB TYR 153 A <--> 10525 N SER 565 B 3.88

8. 1326 CB TYR 153 A <--> 10529 OG SER 565 B 3.55

9. 1329 CD2 TYR 153 A <--> 10528 CB SER 565 B 3.78

10. 1330 CE1 TYR 153 A <--> 10250 O CYS 537 B 3.63

11. 1330 CE1 TYR 153 A <--> 10251 CB CYS 537 B 3.41

12. 1330 CE1 TYR 153 A <--> 10510 CB CYS 562 B 3.86

13. 1330 CE1 TYR 153 A <--> 10511 SG CYS 562 B 3.73

14. 1331 CE2 TYR 153 A <--> 10541 CB LYS 567 B 3.29

15. 1331 CE2 TYR 153 A <--> 10542 CG LYS 567 B 3.77

16. 1332 CZ TYR 153 A <--> 10251 CB CYS 537 B 3.89

17. 1332 CZ TYR 153 A <--> 10510 CB CYS 562 B 3.87

18. 1332 CZ TYR 153 A <--> 10511 SG CYS 562 B 3.41

19. 1333 OH TYR 153 A <--> 10251 CB CYS 537 B 3.48

20. 1333 OH TYR 153 A <--> 10511 SG CYS 562 B 2.98

21. 1333 OH TYR 153 A <--> 10541 CB LYS 567 B 3.88

22. 1342 OD1 ASN 154 A <--> 10543 CD LYS 567 B 3.82

23. 1342 OD1 ASN 154 A <--> 10544 CE LYS 567 B 3.71

24. 1342 OD1 ASN 154 A <--> 10545 NZ LYS 567 B 2.92

25. 1368 NE ARG 156 A <--> 10250 O CYS 537 B 3.49

26. 1368 NE ARG 156 A <--> 10256 CB SER 538 B 3.89

27. 1370 CZ ARG 156 A <--> 10242 OD1 ASP 536 B 3.69

28. 1370 CZ ARG 156 A <--> 10250 O CYS 537 B 3.69

29. 1370 CZ ARG 156 A <--> 10256 CB SER 538 B 3.72

30. 1371 NH1 ARG 156 A <--> 10242 OD1 ASP 536 B 3.81

31. 1374 NH2 ARG 156 A <--> 10241 CG ASP 536 B 3.23

32. 1374 NH2 ARG 156 A <--> 10242 OD1 ASP 536 B 2.72

33. 1374 NH2 ARG 156 A <--> 10243 OD2 ASP 536 B 3.29

34. 1374 NH2 ARG 156 A <--> 10249 C CYS 537 B 3.63

35. 1374 NH2 ARG 156 A <--> 10250 O CYS 537 B 3.04

36. 1374 NH2 ARG 156 A <--> 10256 CB SER 538 B 3.56

37. 1374 NH2 ARG 156 A <--> 10257 OG SER 538 B 3.88

38. 1382 CB ALA 157 A <--> 10596 CG2 ILE 572 B 3.90

39. 1421 O ARG 160 A <--> 10619 CG PRO 575 B 3.59

40. 1409 CG ARG 160 A <--> 10608 O ILE 573 B 3.71

41. 1410 CD ARG 160 A <--> 10266 CB CYS 539 B 3.55

42. 1413 CZ ARG 160 A <--> 10261 N CYS 539 B 3.59

43. 1414 NH1 ARG 160 A <--> 10261 N CYS 539 B 3.48

44. 1414 NH1 ARG 160 A <--> 10263 CA CYS 539 B 3.44

45. 1414 NH1 ARG 160 A <--> 10266 CB CYS 539 B 3.62

46. 1414 NH1 ARG 160 A <--> 10615 SG CYS 574 B 3.03

47. 1417 NH2 ARG 160 A <--> 10596 CG2 ILE 572 B 3.47

48. 1417 NH2 ARG 160 A <--> 10597 CD1 ILE 572 B 3.59

49. 1425 CB SER 161 A <--> 10608 O ILE 573 B 3.73

50. 1426 OG SER 161 A <--> 10600 N ILE 573 B 2.93

51. 1426 OG SER 161 A <--> 10602 CA ILE 573 B 3.57

52. 1426 OG SER 161 A <--> 10607 C ILE 573 B 3.54

53. 1426 OG SER 161 A <--> 10608 O ILE 573 B 2.77

54. 1426 OG SER 161 A <--> 10603 CB ILE 573 B 3.86

55. 1458 CD2 PHE 164 A <--> 10605 CG2 ILE 573 B 3.67

56. 1469 CD ARG 165 A <--> 10606 CD1 ILE 573 B 3.75

57. 1470 NE ARG 165 A <--> 10606 CD1 ILE 573 B 3.69

58. 1472 CZ ARG 165 A <--> 10606 CD1 ILE 573 B 3.81

59. 1476 NH2 ARG 165 A <--> 10589 C SER 571 B 3.85

60. 1476 NH2 ARG 165 A <--> 10590 O SER 571 B 3.80

61. 1885 O ILE 211 A <--> 10527 CA SER 565 B 3.71

62. 1885 O ILE 211 A <--> 10531 C SER 565 B 3.71

63. 1885 O ILE 211 A <--> 10528 CB SER 565 B 3.59

64. 1885 O ILE 211 A <--> 10533 N GLY 566 B 2.79

65. 1885 O ILE 211 A <--> 10535 CA GLY 566 B 3.63

66. 1891 OD1 ASN 212 A <--> 10537 O GLY 566 B 3.84

67. 1897 N ILE 213 A <--> 10537 O GLY 566 B 3.25

68. 1904 C ILE 213 A <--> 10537 O GLY 566 B 3.75

69. 1902 CG2 ILE 213 A <--> 10537 O GLY 566 B 3.73

70. 1902 CG2 ILE 213 A <--> 10543 CD LYS 567 B 3.52

71. 1906 N THR 214 A <--> 10537 O GLY 566 B 3.33

72. 1909 CB THR 214 A <--> 10499 NZ LYS 561 B 3.60

73. 1910 OG1 THR 214 A <--> 10497 CD LYS 561 B 3.35

74. 1910 OG1 THR 214 A <--> 10498 CE LYS 561 B 3.48

75. 1910 OG1 THR 214 A <--> 10499 NZ LYS 561 B 2.97

76. 1910 OG1 THR 214 A <--> 10536 C GLY 566 B 3.50

77. 1910 OG1 THR 214 A <--> 10537 O GLY 566 B 2.94

78. 1912 CG2 THR 214 A <--> 10498 CE LYS 561 B 3.69

79. 1912 CG2 THR 214 A <--> 10499 NZ LYS 561 B 3.49

80. 1950 OE1 GLN 217 A <--> 10587 OG SER 571 B 3.80

81. 2053 CA GLY 229 A <--> 5309 CZ ARG 32 B 3.81

82. 2053 CA GLY 229 A <--> 5310 NH1 ARG 32 B 3.89

83. 2053 CA GLY 229 A <--> 5313 NH2 ARG 32 B 3.47

84. 2055 O GLY 229 A <--> 5310 NH1 ARG 32 B 3.76

85. 2092 CG2 VAL 233 A <--> 5306 CD ARG 32 B 3.80

86. 2092 CG2 VAL 233 A <--> 5310 NH1 ARG 32 B 3.49

87. 2121 CB ASN 236 A <--> 5297 OD1 ASP 31 B 3.71

88. 2123 OD1 ASN 236 A <--> 5288 C CYS 30 B 3.82

89. 2123 OD1 ASN 236 A <--> 5289 O CYS 30 B 3.09

90. 2123 OD1 ASN 236 A <--> 5294 CA ASP 31 B 3.39

91. 2158 CE1 PHE 240 A <--> 5356 CE LYS 37 B 3.57

92. 2160 CZ PHE 240 A <--> 5356 CE LYS 37 B 3.57

93. 2586 O GLY 287 A <--> 5260 N SER 27 B 2.85

94. 2586 O GLY 287 A <--> 5268 N LEU 28 B 3.71

95. 2587 N PRO 288 A <--> 5276 O LEU 28 B 3.71

96. 2588 CA PRO 288 A <--> 5276 O LEU 28 B 3.38

97. 2589 CB PRO 288 A <--> 5276 O LEU 28 B 3.65

98. 2602 CB VAL 290 A <--> 5260 N SER 27 B 3.26

99. 2604 CG2 VAL 290 A <--> 5260 N SER 27 B 3.76

100. 4998 CD GLU 540 A <--> 5586 NH2 ARG 63 B 3.38

101. 4999 OE1 GLU 540 A <--> 5580 NE ARG 63 B 3.67

102. 4999 OE1 GLU 540 A <--> 5582 CZ ARG 63 B 3.60

103. 4999 OE1 GLU 540 A <--> 5586 NH2 ARG 63 B 2.67

104. 5000 OE2 GLU 540 A <--> 5586 NH2 ARG 63 B 3.38

105. 5005 CA GLY 541 A <--> 5558 ND2 ASN 61 B 3.80

106. 5006 C GLY 541 A <--> 5558 ND2 ASN 61 B 3.36

107. 5007 O GLY 541 A <--> 5558 ND2 ASN 61 B 3.75

108. 5008 N PHE 542 A <--> 5558 ND2 ASN 61 B 3.31

109. 5010 CA PHE 542 A <--> 5558 ND2 ASN 61 B 3.74

110. 5018 C PHE 542 A <--> 5558 ND2 ASN 61 B 3.58

111. 5019 O PHE 542 A <--> 5558 ND2 ASN 61 B 3.02

112. 5023 CB ASN 543 A <--> 5798 CB SER 85 B 3.77

113. 5034 CB LYS 544 A <--> 6045 OH TYR 111 B 3.33

114. 5035 CG LYS 544 A <--> 6045 OH TYR 111 B 3.84

115. 5038 NZ LYS 544 A <--> 5813 O ASN 86 B 2.83

116. 5049 CD LYS 545 A <--> 6017 CE1 TYR 109 B 3.45

117. 5050 CE LYS 545 A <--> 6518 OD2 ASP 160 B 3.48

118. 5051 NZ LYS 545 A <--> 6516 CG ASP 160 B 3.12

119. 5051 NZ LYS 545 A <--> 6517 OD1 ASP 160 B 2.95

120. 5051 NZ LYS 545 A <--> 6518 OD2 ASP 160 B 2.69

121. 5072 CG LYS 547 A <--> 6018 CE2 TYR 109 B 3.52

122. 5072 CG LYS 547 A <--> 6019 CZ TYR 109 B 3.68

123. 5072 CG LYS 547 A <--> 6020 OH TYR 109 B 3.36

124. 5074 CE LYS 547 A <--> 6020 OH TYR 109 B 3.42

125. 5075 NZ LYS 547 A <--> 6020 OH TYR 109 B 2.89

126. 5094 SG CYS 549 A <--> 5558 ND2 ASN 61 B 3.36

127. 5094 SG CYS 549 A <--> 5799 OG SER 85 B 3.35

128. 5121 CA ALA 553 A <--> 5557 OD1 ASN 61 B 3.40

129. 5122 CB ALA 553 A <--> 5557 OD1 ASN 61 B 3.17

130. 5129 O GLY 554 A <--> 5792 CG2 THR 84 B 3.37

131. 5158 CB CYS 557 A <--> 5799 OG SER 85 B 3.70

132. 5159 SG CYS 557 A <--> 5557 OD1 ASN 61 B 3.64

133. 5159 SG CYS 557 A <--> 5558 ND2 ASN 61 B 3.73

134. 5159 SG CYS 557 A <--> 5799 OG SER 85 B 3.36

135. 5211 CE1 HIS 562 A <--> 7200 NE ARG 230 B 3.78

136. 5211 CE1 HIS 562 A <--> 7202 CZ ARG 230 B 3.46

137. 5211 CE1 HIS 562 A <--> 7203 NH1 ARG 230 B 3.48

138. 5211 CE1 HIS 562 A <--> 7206 NH2 ARG 230 B 3.84

139. 5211 CE1 HIS 562 A <--> 7471 CD ARG 257 B 3.88

140. 5247 O GLU 565 A <--> 7486 CB ASN 258 B 3.90

141. 5247 O GLU 565 A <--> 7487 CG ASN 258 B 3.37

142. 5247 O GLU 565 A <--> 7488 OD1 ASN 258 B 3.79

143. 5247 O GLU 565 A <--> 7489 ND2 ASN 258 B 3.19

144. 5258 C HIS 566 A <--> 7757 CB ASP 286 B 3.86

145. 5258 C HIS 566 A <--> 7758 CG ASP 286 B 3.57

146. 5258 C HIS 566 A <--> 7760 OD2 ASP 286 B 3.17

147. 5259 O HIS 566 A <--> 7758 CG ASP 286 B 3.81

148. 5259 O HIS 566 A <--> 7760 OD2 ASP 286 B 3.28

149. 5251 CB HIS 566 A <--> 7470 CG ARG 257 B 3.60

150. 5251 CB HIS 566 A <--> 7471 CD ARG 257 B 3.63

151. 5253 ND1 HIS 566 A <--> 7197 CB ARG 230 B 3.79

152. 5253 ND1 HIS 566 A <--> 7482 O ARG 257 B 3.68

153. 5253 ND1 HIS 566 A <--> 7486 CB ASN 258 B 3.83

154. 5255 CE1 HIS 566 A <--> 7197 CB ARG 230 B 3.49

155. 5255 CE1 HIS 566 A <--> 7211 N ASP 231 B 3.73

Salt bridges

<----- A T O M 1 -----> <----- A T O M 2 ----->

Atom Atom Res Res Atom Atom Res Res

no. name name no. Chain no. name name no. Chain Distance

1. 1374 NH2 ARG 156 A <--> 10242 OD1 ASP 536 B 2.72

2. 5000 OE2 GLU 540 A <--> 5586 NH2 ARG 63 B 2.67

3. 5051 NZ LYS 545 A <--> 6518 OD2 ASP 160 B 2.69

**Supplementary Material SM3.** Vaccine (Chain A)‌-TLR4 (Chain B) PDBsum interacting molecules.

Hydrogen bonds

<----- A T O M 1 -----> <----- A T O M 2 ----->

Atom Atom Res Res Atom Atom Res Res

no. name name no. Chain no. name name no. Chain Distance

1. 1226 O ALA 142 A <--> 5459 NZ LYS 47 B 2.94

2. 1333 OH TYR 153 A <--> 5465 N ILE 48 B 3.19

3. 1414 NH1 ARG 160 A <--> 5694 O SER 71 B 2.77

4. 1414 NH1 ARG 160 A <--> 5922 OE1 GLU 94 B 3.12

5. 1417 NH2 ARG 160 A <--> 5694 O SER 71 B 3.33

6. 1426 OG SER 161 A <--> 5666 ND1 HIS 68 B 2.87

7. 1426 OG SER 161 A <--> 5666 ND1 HIS 68 B 2.87

8. 1470 NE ARG 165 A <--> 5643 O LEU 66 B 3.09

9. 1476 NH2 ARG 165 A <--> 5634 O PRO 65 B 2.98

10. 1476 NH2 ARG 165 A <--> 5874 OE2 GLU 89 B 2.75

11. 1872 N GLY 210 A <--> 5487 OD2 ASP 50 B 3.29

12. 1877 N ILE 211 A <--> 5487 OD2 ASP 50 B 3.07

13. 1892 ND2 ASN 212 A <--> 5495 OD1 ASN 51 B 2.98

14. 1897 N ILE 213 A <--> 5486 OD1 ASP 50 B 2.93

15. 1950 OE1 GLN 217 A <--> 5438 N TYR 46 B 2.83

16. 1950 OE1 GLN 217 A <--> 5452 N LYS 47 B 3.18

17. 2019 NH1 ARG 224 A <--> 5403 OE2 GLU 42 B 2.70

18. 2022 NH2 ARG 224 A <--> 5402 OE1 GLU 42 B 2.61

19. 2335 NH2 ARG 257 A <--> 5267 OE2 GLU 27 B 2.62

20. 2335 NH2 ARG 257 A <--> 5495 OD1 ASN 51 B 2.85

21. 2344 OG SER 258 A <--> 5260 N GLU 27 B 3.13

22. 5212 NE2 HIS 562 A <--> 10754 OG SER 592 B 2.87

23. 5247 O GLU 565 A <--> 10556 ND2 ASN 571 B 3.11

Non-bonded contacts

<----- A T O M 1 -----> <----- A T O M 2 ----->

Atom Atom Res Res Atom Atom Res Res

no. name name no. Chain no. name name no. Chain Distance

1. 1225 C ALA 142 A <--> 5459 NZ LYS 47 B 3.86

2. 1226 O ALA 142 A <--> 5459 NZ LYS 47 B 2.94

3. 1326 CB TYR 153 A <--> 5734 CD1 PHE 75 B 3.70

4. 1326 CB TYR 153 A <--> 5736 CE1 PHE 75 B 3.78

5. 1328 CD1 TYR 153 A <--> 5711 CA SER 73 B 3.77

6. 1328 CD1 TYR 153 A <--> 5716 O SER 73 B 3.88

7. 1329 CD2 TYR 153 A <--> 5736 CE1 PHE 75 B 3.85

8. 1330 CE1 TYR 153 A <--> 5711 CA SER 73 B 3.89

9. 1331 CE2 TYR 153 A <--> 5465 N ILE 48 B 3.75

10. 1332 CZ TYR 153 A <--> 5465 N ILE 48 B 3.80

11. 1332 CZ TYR 153 A <--> 5471 CD1 ILE 48 B 3.85

12. 1333 OH TYR 153 A <--> 5454 CA LYS 47 B 3.59

13. 1333 OH TYR 153 A <--> 5463 C LYS 47 B 3.88

14. 1333 OH TYR 153 A <--> 5465 N ILE 48 B 3.19

15. 1333 OH TYR 153 A <--> 5471 CD1 ILE 48 B 3.34

16. 1410 CD ARG 160 A <--> 5921 CD GLU 94 B 3.65

17. 1410 CD ARG 160 A <--> 5922 OE1 GLU 94 B 3.18

18. 1410 CD ARG 160 A <--> 5923 OE2 GLU 94 B 3.34

19. 1411 NE ARG 160 A <--> 5922 OE1 GLU 94 B 3.63

20. 1413 CZ ARG 160 A <--> 5684 CA GLY 70 B 3.65

21. 1413 CZ ARG 160 A <--> 5685 C GLY 70 B 3.83

22. 1413 CZ ARG 160 A <--> 5687 N SER 71 B 3.58

23. 1413 CZ ARG 160 A <--> 5694 O SER 71 B 3.47

24. 1413 CZ ARG 160 A <--> 5922 OE1 GLU 94 B 3.61

25. 1414 NH1 ARG 160 A <--> 5685 C GLY 70 B 3.79

26. 1414 NH1 ARG 160 A <--> 5687 N SER 71 B 3.11

27. 1414 NH1 ARG 160 A <--> 5689 CA SER 71 B 3.66

28. 1414 NH1 ARG 160 A <--> 5693 C SER 71 B 3.59

29. 1414 NH1 ARG 160 A <--> 5694 O SER 71 B 2.77

30. 1414 NH1 ARG 160 A <--> 5690 CB SER 71 B 3.77

31. 1414 NH1 ARG 160 A <--> 5922 OE1 GLU 94 B 3.12

32. 1417 NH2 ARG 160 A <--> 5684 CA GLY 70 B 3.60

33. 1417 NH2 ARG 160 A <--> 5685 C GLY 70 B 3.74

34. 1417 NH2 ARG 160 A <--> 5687 N SER 71 B 3.85

35. 1417 NH2 ARG 160 A <--> 5694 O SER 71 B 3.33

36. 1425 CB SER 161 A <--> 5666 ND1 HIS 68 B 3.85

37. 1426 OG SER 161 A <--> 5664 CB HIS 68 B 3.80

38. 1426 OG SER 161 A <--> 5665 CG HIS 68 B 3.41

39. 1426 OG SER 161 A <--> 5666 ND1 HIS 68 B 2.87

40. 1426 OG SER 161 A <--> 5668 CE1 HIS 68 B 3.46

41. 1463 O PHE 164 A <--> 5667 CD2 HIS 68 B 3.79

42. 1458 CD2 PHE 164 A <--> 5902 OG1 THR 92 B 3.43

43. 1460 CE2 PHE 164 A <--> 5893 NE2 GLN 91 B 3.55

44. 1460 CE2 PHE 164 A <--> 5902 OG1 THR 92 B 3.21

45. 1468 CG ARG 165 A <--> 5664 CB HIS 68 B 3.84

46. 1470 NE ARG 165 A <--> 5643 O LEU 66 B 3.09

47. 1472 CZ ARG 165 A <--> 5643 O LEU 66 B 3.49

48. 1472 CZ ARG 165 A <--> 5647 CB ARG 67 B 3.55

49. 1472 CZ ARG 165 A <--> 5874 OE2 GLU 89 B 3.62

50. 1473 NH1 ARG 165 A <--> 5649 CD ARG 67 B 3.78

51. 1473 NH1 ARG 165 A <--> 5874 OE2 GLU 89 B 3.69

52. 1476 NH2 ARG 165 A <--> 5443 CD1 TYR 46 B 3.77

53. 1476 NH2 ARG 165 A <--> 5633 C PRO 65 B 3.43

54. 1476 NH2 ARG 165 A <--> 5634 O PRO 65 B 2.98

55. 1476 NH2 ARG 165 A <--> 5630 CB PRO 65 B 3.83

56. 1476 NH2 ARG 165 A <--> 5642 C LEU 66 B 3.50

57. 1476 NH2 ARG 165 A <--> 5643 O LEU 66 B 3.20

58. 1476 NH2 ARG 165 A <--> 5647 CB ARG 67 B 3.37

59. 1476 NH2 ARG 165 A <--> 5874 OE2 GLU 89 B 2.75

60. 1514 CB ALA 169 A <--> 5448 OH TYR 46 B 3.31

61. 1866 O PRO 208 A <--> 5525 CE2 PHE 54 B 3.90

62. 1866 O PRO 208 A <--> 5526 CZ PHE 54 B 3.20

63. 1862 CB PRO 208 A <--> 5511 CA PRO 53 B 3.81

64. 1863 CG PRO 208 A <--> 5500 O ASN 51 B 3.43

65. 1867 N GLY 209 A <--> 5489 O ASP 50 B 3.34

66. 1869 CA GLY 209 A <--> 5489 O ASP 50 B 3.27

67. 1869 CA GLY 209 A <--> 5487 OD2 ASP 50 B 3.55

68. 1870 C GLY 209 A <--> 5487 OD2 ASP 50 B 3.81

69. 1872 N GLY 210 A <--> 5487 OD2 ASP 50 B 3.29

70. 1877 N ILE 211 A <--> 5487 OD2 ASP 50 B 3.07

71. 1879 CA ILE 211 A <--> 5487 OD2 ASP 50 B 3.66

72. 1884 C ILE 211 A <--> 5486 OD1 ASP 50 B 3.83

73. 1884 C ILE 211 A <--> 5487 OD2 ASP 50 B 3.22

74. 1885 O ILE 211 A <--> 5487 OD2 ASP 50 B 3.18

75. 1881 CG1 ILE 211 A <--> 5487 OD2 ASP 50 B 3.88

76. 1886 N ASN 212 A <--> 5486 OD1 ASP 50 B 3.46

77. 1886 N ASN 212 A <--> 5487 OD2 ASP 50 B 3.66

78. 1888 CA ASN 212 A <--> 5486 OD1 ASP 50 B 3.35

79. 1895 C ASN 212 A <--> 5486 OD1 ASP 50 B 3.61

80. 1892 ND2 ASN 212 A <--> 5493 CB ASN 51 B 3.59

81. 1892 ND2 ASN 212 A <--> 5494 CG ASN 51 B 3.60

82. 1892 ND2 ASN 212 A <--> 5495 OD1 ASN 51 B 2.98

83. 1897 N ILE 213 A <--> 5486 OD1 ASP 50 B 2.93

84. 1902 CG2 ILE 213 A <--> 5473 O ILE 48 B 3.43

85. 1902 CG2 ILE 213 A <--> 5486 OD1 ASP 50 B 3.59

86. 1903 CD1 ILE 213 A <--> 5456 CG LYS 47 B 3.76

87. 1909 CB THR 214 A <--> 5475 CA PRO 49 B 3.78

88. 1909 CB THR 214 A <--> 5476 CB PRO 49 B 3.62

89. 1910 OG1 THR 214 A <--> 5273 CG PRO 28 B 3.48

90. 1910 OG1 THR 214 A <--> 5493 CB ASN 51 B 3.55

91. 1912 CG2 THR 214 A <--> 5475 CA PRO 49 B 3.18

92. 1912 CG2 THR 214 A <--> 5479 C PRO 49 B 3.54

93. 1912 CG2 THR 214 A <--> 5476 CB PRO 49 B 3.41

94. 1912 CG2 THR 214 A <--> 5481 N ASP 50 B 3.21

95. 1912 CG2 THR 214 A <--> 5486 OD1 ASP 50 B 3.81

96. 1912 CG2 THR 214 A <--> 5490 N ASN 51 B 3.26

97. 1912 CG2 THR 214 A <--> 5493 CB ASN 51 B 3.31

98. 1938 CD2 PHE 216 A <--> 5458 CE LYS 47 B 3.82

99. 1940 CE2 PHE 216 A <--> 5458 CE LYS 47 B 3.88

100. 1946 CA GLN 217 A <--> 5425 O ASN 44 B 3.66

101. 1954 C GLN 217 A <--> 5414 O LEU 43 B 3.56

102. 1955 O GLN 217 A <--> 5414 O LEU 43 B 3.16

103. 1955 O GLN 217 A <--> 5418 CB ASN 44 B 3.75

104. 1947 CB GLN 217 A <--> 5424 C ASN 44 B 3.84

105. 1947 CB GLN 217 A <--> 5425 O ASN 44 B 3.21

106. 1947 CB GLN 217 A <--> 5428 CA PHE 45 B 3.71

107. 1947 CB GLN 217 A <--> 5431 CD1 PHE 45 B 3.74

108. 1948 CG GLN 217 A <--> 5431 CD1 PHE 45 B 3.44

109. 1948 CG GLN 217 A <--> 5433 CE1 PHE 45 B 3.77

110. 1949 CD GLN 217 A <--> 5438 N TYR 46 B 3.78

111. 1949 CD GLN 217 A <--> 5452 N LYS 47 B 3.65

112. 1949 CD GLN 217 A <--> 5455 CB LYS 47 B 3.88

113. 1950 OE1 GLN 217 A <--> 5425 O ASN 44 B 3.65

114. 1950 OE1 GLN 217 A <--> 5436 C PHE 45 B 3.84

115. 1950 OE1 GLN 217 A <--> 5438 N TYR 46 B 2.83

116. 1950 OE1 GLN 217 A <--> 5440 CA TYR 46 B 3.50

117. 1950 OE1 GLN 217 A <--> 5450 C TYR 46 B 3.74

118. 1950 OE1 GLN 217 A <--> 5441 CB TYR 46 B 3.44

119. 1950 OE1 GLN 217 A <--> 5452 N LYS 47 B 3.18

120. 1950 OE1 GLN 217 A <--> 5457 CD LYS 47 B 3.57

121. 1951 NE2 GLN 217 A <--> 5455 CB LYS 47 B 3.41

122. 1951 NE2 GLN 217 A <--> 5457 CD LYS 47 B 3.45

123. 1951 NE2 GLN 217 A <--> 5458 CE LYS 47 B 3.81

124. 1960 OG1 THR 218 A <--> 5272 CB PRO 28 B 3.27

125. 1981 C LEU 220 A <--> 5420 OD1 ASN 44 B 3.70

126. 1982 O LEU 220 A <--> 5420 OD1 ASN 44 B 3.69

127. 1977 CB LEU 220 A <--> 5420 OD1 ASN 44 B 3.30

128. 1979 CD1 LEU 220 A <--> 5444 CD2 TYR 46 B 3.89

129. 1979 CD1 LEU 220 A <--> 5446 CE2 TYR 46 B 3.87

130. 1983 N ALA 221 A <--> 5418 CB ASN 44 B 3.79

131. 1985 CA ALA 221 A <--> 5418 CB ASN 44 B 3.85

132. 1986 CB ALA 221 A <--> 5413 C LEU 43 B 3.75

133. 1986 CB ALA 221 A <--> 5414 O LEU 43 B 3.60

134. 2018 CZ ARG 224 A <--> 5401 CD GLU 42 B 3.88

135. 2018 CZ ARG 224 A <--> 5402 OE1 GLU 42 B 3.55

136. 2018 CZ ARG 224 A <--> 5403 OE2 GLU 42 B 3.43

137. 2019 NH1 ARG 224 A <--> 5401 CD GLU 42 B 3.53

138. 2019 NH1 ARG 224 A <--> 5402 OE1 GLU 42 B 3.64

139. 2019 NH1 ARG 224 A <--> 5403 OE2 GLU 42 B 2.70

140. 2022 NH2 ARG 224 A <--> 5401 CD GLU 42 B 3.30

141. 2022 NH2 ARG 224 A <--> 5402 OE1 GLU 42 B 2.61

142. 2022 NH2 ARG 224 A <--> 5403 OE2 GLU 42 B 3.29

143. 2022 NH2 ARG 224 A <--> 5418 CB ASN 44 B 3.60

144. 2022 NH2 ARG 224 A <--> 5419 CG ASN 44 B 3.31

145. 2022 NH2 ARG 224 A <--> 5421 ND2 ASN 44 B 3.00

146. 2244 CG2 THR 250 A <--> 5298 OE1 GLU 31 B 3.48

147. 2299 O ARG 254 A <--> 5260 N GLU 27 B 3.54

148. 2338 C ARG 257 A <--> 5260 N GLU 27 B 3.73

149. 2326 CB ARG 257 A <--> 5260 N GLU 27 B 3.29

150. 2329 NE ARG 257 A <--> 5264 CG GLU 27 B 3.64

151. 2329 NE ARG 257 A <--> 5267 OE2 GLU 27 B 3.38

152. 2331 CZ ARG 257 A <--> 5267 OE2 GLU 27 B 3.43

153. 2335 NH2 ARG 257 A <--> 5265 CD GLU 27 B 3.71

154. 2335 NH2 ARG 257 A <--> 5267 OE2 GLU 27 B 2.62

155. 2335 NH2 ARG 257 A <--> 5494 CG ASN 51 B 3.61

156. 2335 NH2 ARG 257 A <--> 5495 OD1 ASN 51 B 2.85

157. 2335 NH2 ARG 257 A <--> 5496 ND2 ASN 51 B 3.59

158. 2340 N SER 258 A <--> 5260 N GLU 27 B 3.47

159. 2344 OG SER 258 A <--> 5260 N GLU 27 B 3.13

160. 2344 OG SER 258 A <--> 5269 O GLU 27 B 3.24

161. 5210 CD2 HIS 562 A <--> 10754 OG SER 592 B 3.88

162. 5211 CE1 HIS 562 A <--> 10753 CB SER 592 B 3.88

163. 5211 CE1 HIS 562 A <--> 10754 OG SER 592 B 3.77

164. 5212 NE2 HIS 562 A <--> 10753 CB SER 592 B 3.37

165. 5212 NE2 HIS 562 A <--> 10754 OG SER 592 B 2.87

166. 5247 O GLU 565 A <--> 10554 CG ASN 571 B 3.74

167. 5247 O GLU 565 A <--> 10555 OD1 ASN 571 B 3.52

168. 5247 O GLU 565 A <--> 10556 ND2 ASN 571 B 3.11

169. 5247 O GLU 565 A <--> 10773 CD1 ILE 594 B 3.81

170. 5250 CA HIS 566 A <--> 10556 ND2 ASN 571 B 3.21

171. 5251 CB HIS 566 A <--> 10556 ND2 ASN 571 B 3.76

172. 5251 CB HIS 566 A <--> 10749 O ARG 591 B 3.14

173. 5252 CG HIS 566 A <--> 10749 O ARG 591 B 3.67

174. 5252 CG HIS 566 A <--> 10752 CA SER 592 B 3.73

175. 5253 ND1 HIS 566 A <--> 10556 ND2 ASN 571 B 3.64

176. 5253 ND1 HIS 566 A <--> 10749 O ARG 591 B 3.71

177. 5253 ND1 HIS 566 A <--> 10752 CA SER 592 B 3.48

178. 5253 ND1 HIS 566 A <--> 10756 C SER 592 B 3.53

179. 5253 ND1 HIS 566 A <--> 10757 O SER 592 B 3.74

180. 5253 ND1 HIS 566 A <--> 10771 CG1 ILE 594 B 3.72

181. 5253 ND1 HIS 566 A <--> 10773 CD1 ILE 594 B 3.75

182. 5255 CE1 HIS 566 A <--> 10752 CA SER 592 B 3.82

183. 5255 CE1 HIS 566 A <--> 10756 C SER 592 B 3.46

184. 5255 CE1 HIS 566 A <--> 10757 O SER 592 B 3.23

185. 5255 CE1 HIS 566 A <--> 10771 CG1 ILE 594 B 3.69

186. 5255 CE1 HIS 566 A <--> 10773 CD1 ILE 594 B 3.71

187. 5256 NE2 HIS 566 A <--> 10757 O SER 592 B 3.65

Salt bridges

<----- A T O M 1 -----> <----- A T O M 2 ----->

Atom Atom Res Res Atom Atom Res Res

no. name name no. Chain no. name name no. Chain Distance

1. 1414 NH1 ARG 160 A <--> 5923 OE2 GLU 94 B 3.12

2. 1476 NH2 ARG 165 A <--> 5874 OE2 GLU 89 B 2.75

3. 2022 NH2 ARG 224 A <--> 5403 OE2 GLU 42 B 2.61

4. 2278 NH2 ARG 253 A <--> 5298 OE1 GLU 31 B 3.93

5. 2335 NH2 ARG 257 A <--> 5267 OE2 GLU 27 B 2.62

**Supplementary Material SM4.** Vaccine (Chain A)‌-MHC-I (Chain B) PDBsum interacting molecules.

Hydrogen bonds

<----- A T O M 1 -----> <----- A T O M 2 ----->

Atom Atom Res Res Atom Atom Res Res

no. name name no. Chain no. name name no. Chain Distance

1. 883 NZ LYS 102 A <--> 5872 OD2 ASP 61 B 3.35

2. 1333 OH TYR 153 A <--> 7020 OE2 GLU 173 B 2.67

3. 1470 NE ARG 165 A <--> 5811 OE2 GLU 55 B 2.67

4. 1476 NH2 ARG 165 A <--> 5772 O TRP 51 B 2.79

5. 1476 NH2 ARG 165 A <--> 5811 OE2 GLU 55 B 3.22

6. 1488 N THR 167 A <--> 5803 O GLN 54 B 3.28

7. 1511 N ALA 169 A <--> 5791 O GLU 53 B 3.27

8. 1910 OG1 THR 214 A <--> 7051 O LYS 176 B 2.83

9. 2022 NH2 ARG 224 A <--> 5789 OE2 GLU 53 B 2.65

10. 2252 OD1 ASN 251 A <--> 7147 NZ LYS 186 B 2.88

11. 2289 NE ARG 254 A <--> 7123 OD1 ASP 183 B 2.85

12. 2292 NH1 ARG 254 A <--> 7661 OD2 ASP 238 B 2.72

13. 2295 NH2 ARG 254 A <--> 7124 OD2 ASP 183 B 2.81

14. 2335 NH2 ARG 257 A <--> 7117 O THR 182 B 2.78

15. 2335 NH2 ARG 257 A <--> 7123 OD1 ASP 183 B 2.74

16. 4937 OG SER 533 A <--> 7992 N ARG 273 B 2.89

17. 5178 OE1 GLU 559 A <--> 7175 N MET 189 B 3.19

18. 5222 OE1 GLU 563 A <--> 8025 N GLU 275 B 2.83

19. 5259 O HIS 566 A <--> 8016 NE1 TRP 274 B 2.84

20. 5253 ND1 HIS 566 A <--> 7198 ND1 HIS 191 B 3.13

Non-bonded contacts

<----- A T O M 1 -----> <----- A T O M 2 ----->

Atom Atom Res Res Atom Atom Res Res

no. name name no. Chain no. name name no. Chain Distance

1. 829 OD2 ASP 95 A <--> 5904 CD ARG 65 B 3.51

2. 880 CG LYS 102 A <--> 5872 OD2 ASP 61 B 3.67

3. 881 CD LYS 102 A <--> 5872 OD2 ASP 61 B 3.36

4. 882 CE LYS 102 A <--> 5872 OD2 ASP 61 B 3.54

5. 883 NZ LYS 102 A <--> 5872 OD2 ASP 61 B 3.35

6. 892 CG PRO 103 A <--> 5822 CG PRO 57 B 3.68

7. 1332 CZ TYR 153 A <--> 7020 OE2 GLU 173 B 3.51

8. 1333 OH TYR 153 A <--> 6328 CE3 TRP 107 B 3.70

9. 1333 OH TYR 153 A <--> 6330 CZ3 TRP 107 B 3.38

10. 1333 OH TYR 153 A <--> 7017 CG GLU 173 B 3.71

11. 1333 OH TYR 153 A <--> 7018 CD GLU 173 B 3.58

12. 1333 OH TYR 153 A <--> 7020 OE2 GLU 173 B 2.67

13. 1342 OD1 ASN 154 A <--> 7045 CE LYS 176 B 3.45

14. 1342 OD1 ASN 154 A <--> 7046 NZ LYS 176 B 3.75

15. 1382 CB ALA 157 A <--> 7018 CD GLU 173 B 3.50

16. 1382 CB ALA 157 A <--> 7019 OE1 GLU 173 B 3.72

17. 1382 CB ALA 157 A <--> 7020 OE2 GLU 173 B 3.63

18. 1417 NH2 ARG 160 A <--> 7018 CD GLU 173 B 3.43

19. 1417 NH2 ARG 160 A <--> 7019 OE1 GLU 173 B 3.23

20. 1417 NH2 ARG 160 A <--> 7020 OE2 GLU 173 B 2.90

21. 1459 CE1 PHE 164 A <--> 6981 CZ ARG 170 B 3.50

22. 1459 CE1 PHE 164 A <--> 6982 NH1 ARG 170 B 3.34

23. 1459 CE1 PHE 164 A <--> 6985 NH2 ARG 170 B 3.79

24. 1461 CZ PHE 164 A <--> 6982 NH1 ARG 170 B 3.83

25. 1468 CG ARG 165 A <--> 5803 O GLN 54 B 3.47

26. 1468 CG ARG 165 A <--> 5811 OE2 GLU 55 B 3.76

27. 1469 CD ARG 165 A <--> 5803 O GLN 54 B 3.42

28. 1469 CD ARG 165 A <--> 5811 OE2 GLU 55 B 3.71

29. 1470 NE ARG 165 A <--> 5803 O GLN 54 B 3.63

30. 1470 NE ARG 165 A <--> 5806 CA GLU 55 B 3.76

31. 1470 NE ARG 165 A <--> 5808 CG GLU 55 B 3.83

32. 1470 NE ARG 165 A <--> 5809 CD GLU 55 B 3.29

33. 1470 NE ARG 165 A <--> 5811 OE2 GLU 55 B 2.67

34. 1472 CZ ARG 165 A <--> 5772 O TRP 51 B 3.70

35. 1472 CZ ARG 165 A <--> 5802 C GLN 54 B 3.87

36. 1472 CZ ARG 165 A <--> 5811 OE2 GLU 55 B 3.37

37. 1472 CZ ARG 165 A <--> 7027 CG ASN 174 B 3.87

38. 1472 CZ ARG 165 A <--> 7028 OD1 ASN 174 B 3.64

39. 1472 CZ ARG 165 A <--> 7029 ND2 ASN 174 B 3.70

40. 1473 NH1 ARG 165 A <--> 5772 O TRP 51 B 3.74

41. 1473 NH1 ARG 165 A <--> 5795 CB GLN 54 B 3.46

42. 1473 NH1 ARG 165 A <--> 5798 OE1 GLN 54 B 3.46

43. 1473 NH1 ARG 165 A <--> 7029 ND2 ASN 174 B 3.71

44. 1476 NH2 ARG 165 A <--> 5772 O TRP 51 B 2.79

45. 1476 NH2 ARG 165 A <--> 5808 CG GLU 55 B 3.53

46. 1476 NH2 ARG 165 A <--> 5809 CD GLU 55 B 3.76

47. 1476 NH2 ARG 165 A <--> 5811 OE2 GLU 55 B 3.22

48. 1476 NH2 ARG 165 A <--> 7027 CG ASN 174 B 3.36

49. 1476 NH2 ARG 165 A <--> 7028 OD1 ASN 174 B 2.93

50. 1476 NH2 ARG 165 A <--> 7029 ND2 ASN 174 B 3.65

51. 1484 CG PRO 166 A <--> 5813 O GLU 55 B 3.87

52. 1484 CG PRO 166 A <--> 5816 CA GLY 56 B 3.71

53. 1484 CG PRO 166 A <--> 5823 CD PRO 57 B 3.72

54. 1485 CD PRO 166 A <--> 5813 O GLU 55 B 3.77

55. 1485 CD PRO 166 A <--> 5814 N GLY 56 B 3.87

56. 1485 CD PRO 166 A <--> 5816 CA GLY 56 B 3.48

57. 1488 N THR 167 A <--> 5803 O GLN 54 B 3.28

58. 1490 CA THR 167 A <--> 5802 C GLN 54 B 3.83

59. 1490 CA THR 167 A <--> 5803 O GLN 54 B 3.17

60. 1494 CG2 THR 167 A <--> 5795 CB GLN 54 B 3.61

61. 1497 N TYR 168 A <--> 5813 O GLU 55 B 3.77

62. 1511 N ALA 169 A <--> 5791 O GLU 53 B 3.27

63. 1513 CA ALA 169 A <--> 5791 O GLU 53 B 3.77

64. 1514 CB ALA 169 A <--> 5790 C GLU 53 B 3.70

65. 1514 CB ALA 169 A <--> 5791 O GLU 53 B 3.38

66. 1514 CB ALA 169 A <--> 5785 CB GLU 53 B 3.84

67. 1902 CG2 ILE 213 A <--> 7044 CD LYS 176 B 3.87

68. 1908 CA THR 214 A <--> 7051 O LYS 176 B 3.61

69. 1914 O THR 214 A <--> 7055 CB GLU 177 B 3.75

70. 1909 CB THR 214 A <--> 7051 O LYS 176 B 3.37

71. 1910 OG1 THR 214 A <--> 7051 O LYS 176 B 2.83

72. 1910 OG1 THR 214 A <--> 7091 O GLN 180 B 3.39

73. 1912 CG2 THR 214 A <--> 7051 O LYS 176 B 3.25

74. 1912 CG2 THR 214 A <--> 7054 CA GLU 177 B 3.87

75. 1954 C GLN 217 A <--> 7055 CB GLU 177 B 3.70

76. 1954 C GLN 217 A <--> 7056 CG GLU 177 B 3.87

77. 1955 O GLN 217 A <--> 7056 CG GLU 177 B 3.65

78. 1955 O GLN 217 A <--> 7059 OE2 GLU 177 B 3.46

79. 1947 CB GLN 217 A <--> 7055 CB GLU 177 B 3.87

80. 1947 CB GLN 217 A <--> 7059 OE2 GLU 177 B 3.64

81. 1948 CG GLN 217 A <--> 7042 CB LYS 176 B 3.70

82. 1956 N THR 218 A <--> 7055 CB GLU 177 B 3.47

83. 1958 CA THR 218 A <--> 7055 CB GLU 177 B 3.56

84. 1958 CA THR 218 A <--> 7056 CG GLU 177 B 3.85

85. 1960 OG1 THR 218 A <--> 7055 CB GLU 177 B 3.24

86. 1977 CB LEU 220 A <--> 5799 NE2 GLN 54 B 3.87

87. 1979 CD1 LEU 220 A <--> 5799 NE2 GLN 54 B 3.80

88. 1985 CA ALA 221 A <--> 5752 CB PRO 50 B 3.79

89. 1986 CB ALA 221 A <--> 5752 CB PRO 50 B 3.83

90. 1986 CB ALA 221 A <--> 5753 CG PRO 50 B 3.65

91. 2016 NE ARG 224 A <--> 5788 OE1 GLU 53 B 3.65

92. 2016 NE ARG 224 A <--> 5789 OE2 GLU 53 B 3.43

93. 2018 CZ ARG 224 A <--> 5789 OE2 GLU 53 B 3.46

94. 2022 NH2 ARG 224 A <--> 5752 CB PRO 50 B 3.87

95. 2022 NH2 ARG 224 A <--> 5787 CD GLU 53 B 3.71

96. 2022 NH2 ARG 224 A <--> 5789 OE2 GLU 53 B 2.65

97. 2244 CG2 THR 250 A <--> 7132 O ALA 184 B 3.53

98. 2244 CG2 THR 250 A <--> 7138 C PRO 185 B 3.61

99. 2244 CG2 THR 250 A <--> 7139 O PRO 185 B 3.12

100. 2244 CG2 THR 250 A <--> 7140 N LYS 186 B 3.90

101. 2244 CG2 THR 250 A <--> 7142 CA LYS 186 B 3.77

102. 2251 CG ASN 251 A <--> 7147 NZ LYS 186 B 3.82

103. 2252 OD1 ASN 251 A <--> 7146 CE LYS 186 B 3.43

104. 2252 OD1 ASN 251 A <--> 7147 NZ LYS 186 B 2.88

105. 2278 NH2 ARG 253 A <--> 7130 CB ALA 184 B 3.80

106. 2288 CD ARG 254 A <--> 7123 OD1 ASP 183 B 3.85

107. 2288 CD ARG 254 A <--> 7355 CB SER 207 B 3.84

108. 2289 NE ARG 254 A <--> 7122 CG ASP 183 B 3.37

109. 2289 NE ARG 254 A <--> 7123 OD1 ASP 183 B 2.85

110. 2289 NE ARG 254 A <--> 7124 OD2 ASP 183 B 3.68

111. 2291 CZ ARG 254 A <--> 7122 CG ASP 183 B 3.75

112. 2291 CZ ARG 254 A <--> 7123 OD1 ASP 183 B 3.56

113. 2291 CZ ARG 254 A <--> 7124 OD2 ASP 183 B 3.62

114. 2291 CZ ARG 254 A <--> 7661 OD2 ASP 238 B 3.80

115. 2291 CZ ARG 254 A <--> 7675 CG2 THR 240 B 3.48

116. 2292 NH1 ARG 254 A <--> 7659 CG ASP 238 B 3.88

117. 2292 NH1 ARG 254 A <--> 7661 OD2 ASP 238 B 2.72

118. 2292 NH1 ARG 254 A <--> 7675 CG2 THR 240 B 3.68

119. 2295 NH2 ARG 254 A <--> 7104 NH2 ARG 181 B 3.75

120. 2295 NH2 ARG 254 A <--> 7122 CG ASP 183 B 3.35

121. 2295 NH2 ARG 254 A <--> 7123 OD1 ASP 183 B 3.41

122. 2295 NH2 ARG 254 A <--> 7124 OD2 ASP 183 B 2.81

123. 2295 NH2 ARG 254 A <--> 7675 CG2 THR 240 B 3.50

124. 2331 CZ ARG 257 A <--> 7117 O THR 182 B 3.79

125. 2331 CZ ARG 257 A <--> 7123 OD1 ASP 183 B 3.82

126. 2335 NH2 ARG 257 A <--> 7116 C THR 182 B 3.84

127. 2335 NH2 ARG 257 A <--> 7117 O THR 182 B 2.78

128. 2335 NH2 ARG 257 A <--> 7122 CG ASP 183 B 3.43

129. 2335 NH2 ARG 257 A <--> 7123 OD1 ASP 183 B 2.74

130. 4930 CA GLY 532 A <--> 8007 C ARG 273 B 3.70

131. 4930 CA GLY 532 A <--> 8008 O ARG 273 B 3.10

132. 4931 C GLY 532 A <--> 8008 O ARG 273 B 3.43

133. 4933 N SER 533 A <--> 8008 O ARG 273 B 3.69

134. 4936 CB SER 533 A <--> 7985 CA LEU 272 B 3.64

135. 4936 CB SER 533 A <--> 7989 CD2 LEU 272 B 3.65

136. 4936 CB SER 533 A <--> 7992 N ARG 273 B 3.90

137. 4937 OG SER 533 A <--> 7181 CE MET 189 B 3.55

138. 4937 OG SER 533 A <--> 7985 CA LEU 272 B 3.21

139. 4937 OG SER 533 A <--> 7990 C LEU 272 B 3.54

140. 4937 OG SER 533 A <--> 7986 CB LEU 272 B 3.39

141. 4937 OG SER 533 A <--> 7989 CD2 LEU 272 B 3.83

142. 4937 OG SER 533 A <--> 7992 N ARG 273 B 2.89

143. 4937 OG SER 533 A <--> 8008 O ARG 273 B 3.14

144. 4945 CG LYS 534 A <--> 7159 CG2 THR 187 B 3.75

145. 5136 CD2 PHE 555 A <--> 7169 CD2 HIS 188 B 3.88

146. 5136 CD2 PHE 555 A <--> 7171 NE2 HIS 188 B 3.35

147. 5138 CE2 PHE 555 A <--> 7171 NE2 HIS 188 B 3.67

148. 5177 CD GLU 559 A <--> 7169 CD2 HIS 188 B 3.56

149. 5177 CD GLU 559 A <--> 7183 O MET 189 B 3.69

150. 5178 OE1 GLU 559 A <--> 7169 CD2 HIS 188 B 3.78

151. 5178 OE1 GLU 559 A <--> 7175 N MET 189 B 3.19

152. 5178 OE1 GLU 559 A <--> 7183 O MET 189 B 3.53

153. 5179 OE2 GLU 559 A <--> 7169 CD2 HIS 188 B 3.11

154. 5214 C HIS 562 A <--> 8016 NE1 TRP 274 B 3.82

155. 5214 C HIS 562 A <--> 8020 CZ2 TRP 274 B 3.76

156. 5215 O HIS 562 A <--> 8016 NE1 TRP 274 B 3.38

157. 5215 O HIS 562 A <--> 8020 CZ2 TRP 274 B 3.87

158. 5207 CB HIS 562 A <--> 8020 CZ2 TRP 274 B 3.66

159. 5209 ND1 HIS 562 A <--> 7196 CB HIS 191 B 3.67

160. 5209 ND1 HIS 562 A <--> 8020 CZ2 TRP 274 B 3.76

161. 5211 CE1 HIS 562 A <--> 7205 O HIS 191 B 3.50

162. 5216 N GLU 563 A <--> 8016 NE1 TRP 274 B 3.78

163. 5216 N GLU 563 A <--> 8020 CZ2 TRP 274 B 3.84

164. 5218 CA GLU 563 A <--> 8014 CD1 TRP 274 B 3.71

165. 5218 CA GLU 563 A <--> 8016 NE1 TRP 274 B 3.21

166. 5218 CA GLU 563 A <--> 8018 CE2 TRP 274 B 3.63

167. 5224 C GLU 563 A <--> 8033 C GLU 275 B 3.79

168. 5225 O GLU 563 A <--> 8033 C GLU 275 B 3.48

169. 5219 CB GLU 563 A <--> 8025 N GLU 275 B 3.77

170. 5219 CB GLU 563 A <--> 8033 C GLU 275 B 3.30

171. 5219 CB GLU 563 A <--> 8034 O GLU 275 B 3.42

172. 5220 CG GLU 563 A <--> 8015 CD2 TRP 274 B 3.59

173. 5220 CG GLU 563 A <--> 8018 CE2 TRP 274 B 3.59

174. 5221 CD GLU 563 A <--> 8025 N GLU 275 B 3.81

175. 5222 OE1 GLU 563 A <--> 8008 O ARG 273 B 3.66

176. 5222 OE1 GLU 563 A <--> 8011 CA TRP 274 B 3.73

177. 5222 OE1 GLU 563 A <--> 8023 C TRP 274 B 3.73

178. 5222 OE1 GLU 563 A <--> 8025 N GLU 275 B 2.83

179. 5222 OE1 GLU 563 A <--> 8027 CA GLU 275 B 3.68

180. 5222 OE1 GLU 563 A <--> 8033 C GLU 275 B 3.61

181. 5222 OE1 GLU 563 A <--> 8034 O GLU 275 B 3.38

182. 5258 C HIS 566 A <--> 7201 CE1 HIS 191 B 3.90

183. 5258 C HIS 566 A <--> 7202 NE2 HIS 191 B 3.81

184. 5258 C HIS 566 A <--> 7806 OE2 GLU 254 B 3.76

185. 5259 O HIS 566 A <--> 7200 CD2 HIS 191 B 3.52

186. 5259 O HIS 566 A <--> 7201 CE1 HIS 191 B 3.56

187. 5259 O HIS 566 A <--> 7202 NE2 HIS 191 B 3.22

188. 5259 O HIS 566 A <--> 7806 OE2 GLU 254 B 3.66

189. 5259 O HIS 566 A <--> 8014 CD1 TRP 274 B 3.43

190. 5259 O HIS 566 A <--> 8016 NE1 TRP 274 B 2.84

191. 5251 CB HIS 566 A <--> 7198 ND1 HIS 191 B 3.48

192. 5251 CB HIS 566 A <--> 7201 CE1 HIS 191 B 3.42

193. 5252 CG HIS 566 A <--> 7198 ND1 HIS 191 B 3.74

194. 5252 CG HIS 566 A <--> 7201 CE1 HIS 191 B 3.72

195. 5253 ND1 HIS 566 A <--> 7198 ND1 HIS 191 B 3.13

196. 5253 ND1 HIS 566 A <--> 7201 CE1 HIS 191 B 3.33

Salt bridges

<----- A T O M 1 -----> <----- A T O M 2 ----->

Atom Atom Res Res Atom Atom Res Res

no. name name no. Chain no. name name no. Chain Distance

1. 883 NZ LYS 102 A <--> 5871 OD1 ASP 61 B 3.35

2. 1417 NH2 ARG 160 A <--> 7020 OE2 GLU 173 B 2.90

3. 1470 NE ARG 165 A <--> 5811 OE2 GLU 55 B 2.67

4. 2022 NH2 ARG 224 A <--> 5788 OE1 GLU 53 B 2.65

5. 2295 NH2 ARG 254 A <--> 7124 OD2 ASP 183 B 2.81

6. 2292 NH1 ARG 254 A <--> 7661 OD2 ASP 238 B 2.72

7. 2335 NH2 ARG 257 A <--> 7123 OD1 ASP 183 B 2.74

8. 5179 OE2 GLU 559 A <--> 7171 NE2 HIS 188 B 3.91

**Supplementary Material SM5.** Vaccine (Chain A)‌-MHC-II (Chain B) PDBsum interacting molecules.

Hydrogen bonds

<----- A T O M 1 -----> <----- A T O M 2 ----->

Atom Atom Res Res Atom Atom Res Res

no. name name no. Chain no. name name no. Chain Distance

1. 1342 OD1 ASN 154 A <--> 6106 NE2 HIS 81 B 2.73

2. 1374 NH2 ARG 156 A <--> 6159 OE1 GLU 87 B 2.67

3. 1910 OG1 THR 214 A <--> 6058 O THR 77 B 2.81

4. 2242 OG1 THR 250 A <--> 5845 OD2 ASP 57 B 2.68

5. 2292 NH1 ARG 254 A <--> 5549 OE2 GLU 28 B 2.64

6. 2295 NH2 ARG 254 A <--> 5549 OE2 GLU 28 B 3.30

7. 4900 NZ LYS 529 A <--> 5292 O PRO 5 B 2.81

8. 4941 N LYS 534 A <--> 5596 OH TYR 32 B 2.82

9. 5179 OE2 GLU 559 A <--> 5810 N GLY 54 B 2.70

Non-bonded contacts

<----- A T O M 1 -----> <----- A T O M 2 ----->

Atom Atom Res Res Atom Atom Res Res

no. name name no. Chain no. name name no. Chain Distance

1. 1326 CB TYR 153 A <--> 6139 O GLY 84 B 3.86

2. 1326 CB TYR 153 A <--> 6145 CG2 VAL 85 B 3.78

3. 1328 CD1 TYR 153 A <--> 6137 CA GLY 84 B 3.59

4. 1328 CD1 TYR 153 A <--> 6138 C GLY 84 B 3.75

5. 1328 CD1 TYR 153 A <--> 6139 O GLY 84 B 3.67

6. 1329 CD2 TYR 153 A <--> 6109 O HIS 81 B 3.61

7. 1329 CD2 TYR 153 A <--> 6104 CD2 HIS 81 B 3.79

8. 1329 CD2 TYR 153 A <--> 6106 NE2 HIS 81 B 3.78

9. 1330 CE1 TYR 153 A <--> 6137 CA GLY 84 B 3.76

10. 1331 CE2 TYR 153 A <--> 6109 O HIS 81 B 3.71

11. 1331 CE2 TYR 153 A <--> 6104 CD2 HIS 81 B 3.89

12. 1331 CE2 TYR 153 A <--> 6106 NE2 HIS 81 B 3.90

13. 1341 CG ASN 154 A <--> 6106 NE2 HIS 81 B 3.87

14. 1342 OD1 ASN 154 A <--> 6104 CD2 HIS 81 B 3.76

15. 1342 OD1 ASN 154 A <--> 6105 CE1 HIS 81 B 3.58

16. 1342 OD1 ASN 154 A <--> 6106 NE2 HIS 81 B 2.73

17. 1370 CZ ARG 156 A <--> 6159 OE1 GLU 87 B 3.56

18. 1371 NH1 ARG 156 A <--> 6159 OE1 GLU 87 B 3.59

19. 1374 NH2 ARG 156 A <--> 6158 CD GLU 87 B 3.82

20. 1374 NH2 ARG 156 A <--> 6159 OE1 GLU 87 B 2.67

21. 1892 ND2 ASN 212 A <--> 6064 CD1 TYR 78 B 3.62

22. 1892 ND2 ASN 212 A <--> 6066 CE1 TYR 78 B 3.62

23. 1902 CG2 ILE 213 A <--> 6104 CD2 HIS 81 B 3.75

24. 1902 CG2 ILE 213 A <--> 6106 NE2 HIS 81 B 3.75

25. 1908 CA THR 214 A <--> 6058 O THR 77 B 3.77

26. 1909 CB THR 214 A <--> 6058 O THR 77 B 3.61

27. 1910 OG1 THR 214 A <--> 6057 C THR 77 B 3.57

28. 1910 OG1 THR 214 A <--> 6058 O THR 77 B 2.81

29. 1910 OG1 THR 214 A <--> 6061 CA TYR 78 B 3.60

30. 1910 OG1 THR 214 A <--> 6062 CB TYR 78 B 3.79

31. 1910 OG1 THR 214 A <--> 6064 CD1 TYR 78 B 3.25

32. 1912 CG2 THR 214 A <--> 6057 C THR 77 B 3.86

33. 1912 CG2 THR 214 A <--> 6058 O THR 77 B 3.75

34. 1912 CG2 THR 214 A <--> 6053 CB THR 77 B 3.68

35. 1912 CG2 THR 214 A <--> 6054 OG1 THR 77 B 3.45

36. 1947 CB GLN 217 A <--> 6056 CG2 THR 77 B 3.43

37. 1948 CG GLN 217 A <--> 6056 CG2 THR 77 B 3.83

38. 1950 OE1 GLN 217 A <--> 6056 CG2 THR 77 B 3.65

39. 1960 OG1 THR 218 A <--> 5982 NE2 GLN 70 B 3.52

40. 1962 CG2 THR 218 A <--> 5979 CG GLN 70 B 3.47

41. 2159 CE2 PHE 240 A <--> 5874 OH TYR 60 B 3.42

42. 2160 CZ PHE 240 A <--> 5874 OH TYR 60 B 3.66

43. 2225 O GLY 247 A <--> 5341 CE3 TRP 9 B 3.45

44. 2225 O GLY 247 A <--> 5343 CZ3 TRP 9 B 3.25

45. 2245 C THR 250 A <--> 5889 CZ2 TRP 61 B 3.56

46. 2246 O THR 250 A <--> 5887 CE2 TRP 61 B 3.73

47. 2246 O THR 250 A <--> 5889 CZ2 TRP 61 B 3.22

48. 2246 O THR 250 A <--> 5891 CH2 TRP 61 B 3.86

49. 2241 CB THR 250 A <--> 5845 OD2 ASP 57 B 3.49

50. 2241 CB THR 250 A <--> 5889 CZ2 TRP 61 B 3.59

51. 2241 CB THR 250 A <--> 5891 CH2 TRP 61 B 3.48

52. 2242 OG1 THR 250 A <--> 5845 OD2 ASP 57 B 2.68

53. 2244 CG2 THR 250 A <--> 5338 NE1 TRP 9 B 3.78

54. 2244 CG2 THR 250 A <--> 5340 CE2 TRP 9 B 3.78

55. 2244 CG2 THR 250 A <--> 5342 CZ2 TRP 9 B 3.71

56. 2251 CG ASN 251 A <--> 5885 NE1 TRP 61 B 3.61

57. 2252 OD1 ASN 251 A <--> 5885 NE1 TRP 61 B 3.64

58. 2253 ND2 ASN 251 A <--> 5843 CG ASP 57 B 3.30

59. 2253 ND2 ASN 251 A <--> 5844 OD1 ASP 57 B 3.35

60. 2253 ND2 ASN 251 A <--> 5845 OD2 ASP 57 B 3.15

61. 2253 ND2 ASN 251 A <--> 5885 NE1 TRP 61 B 3.36

62. 2253 ND2 ASN 251 A <--> 5887 CE2 TRP 61 B 3.77

63. 2253 ND2 ASN 251 A <--> 5889 CZ2 TRP 61 B 3.58

64. 2288 CD ARG 254 A <--> 5882 CG TRP 61 B 3.71

65. 2288 CD ARG 254 A <--> 5883 CD1 TRP 61 B 3.77

66. 2288 CD ARG 254 A <--> 5884 CD2 TRP 61 B 3.62

67. 2288 CD ARG 254 A <--> 5885 NE1 TRP 61 B 3.74

68. 2288 CD ARG 254 A <--> 5887 CE2 TRP 61 B 3.65

69. 2291 CZ ARG 254 A <--> 5549 OE2 GLU 28 B 3.39

70. 2292 NH1 ARG 254 A <--> 5547 CD GLU 28 B 3.55

71. 2292 NH1 ARG 254 A <--> 5548 OE1 GLU 28 B 3.88

72. 2292 NH1 ARG 254 A <--> 5549 OE2 GLU 28 B 2.64

73. 2292 NH1 ARG 254 A <--> 5745 CE2 TYR 47 B 3.88

74. 2292 NH1 ARG 254 A <--> 5746 CZ TYR 47 B 3.78

75. 2292 NH1 ARG 254 A <--> 5747 OH TYR 47 B 3.03

76. 2292 NH1 ARG 254 A <--> 5999 NH2 ARG 71 B 3.32

77. 2295 NH2 ARG 254 A <--> 5364 CD1 LEU 11 B 3.45

78. 2295 NH2 ARG 254 A <--> 5365 CD2 LEU 11 B 3.76

79. 2295 NH2 ARG 254 A <--> 5549 OE2 GLU 28 B 3.30

80. 2295 NH2 ARG 254 A <--> 5573 SG CYS 30 B 3.35

81. 2335 NH2 ARG 257 A <--> 5388 CE1 PHE 13 B 3.83

82. 2344 OG SER 258 A <--> 5952 CD1 LEU 67 B 3.52

83. 4897 CG LYS 529 A <--> 5318 CE2 PHE 7 B 3.89

84. 4898 CD LYS 529 A <--> 5272 CB ARG 4 B 3.71

85. 4898 CD LYS 529 A <--> 5292 O PRO 5 B 3.25

86. 4898 CD LYS 529 A <--> 5318 CE2 PHE 7 B 3.74

87. 4899 CE LYS 529 A <--> 5292 O PRO 5 B 3.52

88. 4900 NZ LYS 529 A <--> 5292 O PRO 5 B 2.81

89. 4935 CA SER 533 A <--> 5596 OH TYR 32 B 3.55

90. 4939 C SER 533 A <--> 5596 OH TYR 32 B 3.64

91. 4936 CB SER 533 A <--> 5596 OH TYR 32 B 3.67

92. 4936 CB SER 533 A <--> 5807 CD2 LEU 53 B 3.65

93. 4941 N LYS 534 A <--> 5595 CZ TYR 32 B 3.46

94. 4941 N LYS 534 A <--> 5596 OH TYR 32 B 2.82

95. 4943 CA LYS 534 A <--> 5596 OH TYR 32 B 3.72

96. 4956 CB PRO 535 A <--> 5316 CD2 PHE 7 B 3.81

97. 4957 CG PRO 535 A <--> 5313 CB PHE 7 B 3.87

98. 4958 CD PRO 535 A <--> 5593 CE1 TYR 32 B 3.56

99. 4958 CD PRO 535 A <--> 5596 OH TYR 32 B 3.78

100. 5133 CB PHE 555 A <--> 5834 CB PRO 56 B 3.83

101. 5133 CB PHE 555 A <--> 5835 CG PRO 56 B 3.55

102. 5134 CG PHE 555 A <--> 5835 CG PRO 56 B 3.82

103. 5135 CD1 PHE 555 A <--> 5835 CG PRO 56 B 3.84

104. 5177 CD GLU 559 A <--> 5810 N GLY 54 B 3.42

105. 5178 OE1 GLU 559 A <--> 5810 N GLY 54 B 3.42

106. 5178 OE1 GLU 559 A <--> 5812 CA GLY 54 B 3.90

107. 5179 OE2 GLU 559 A <--> 5803 CA LEU 53 B 3.46

108. 5179 OE2 GLU 559 A <--> 5808 C LEU 53 B 3.54

109. 5179 OE2 GLU 559 A <--> 5810 N GLY 54 B 2.70

110. 5179 OE2 GLU 559 A <--> 5812 CA GLY 54 B 3.64

111. 5179 OE2 GLU 559 A <--> 5815 N ARG 55 B 3.65

112. 5221 CD GLU 563 A <--> 5807 CD2 LEU 53 B 3.78

113. 5222 OE1 GLU 563 A <--> 5807 CD2 LEU 53 B 3.57

Salt bridges

<----- A T O M 1 -----> <----- A T O M 2 ----->

Atom Atom Res Res Atom Atom Res Res

no. name name no. Chain no. name name no. Chain Distance

1. 1374 NH2 ARG 156 A <--> 6160 OE2 GLU 87 B 2.67

2. 2292 NH1 ARG 254 A <--> 5549 OE2 GLU 28 B 2.64

**Supplementary Material SM6.** Codon usage was adapted to *Escherichia coli* (strain K12) and the cDNA sequence of the vaccine construct.

ATGGCTAAACTGTCTACCGACGAACTGCTGGACGCTTTCAAAGAAATGACCCTGCTGGAACTGTCTGACTTCGTTAAAAAATTCGAAGAAACCTTCGAAGTTACCGCTGCTGCTCCGGTTGCTGTTGCTGCTGCTGGTGCTGCTCCGGCTGGTGCTGCTGTTGAAGCTGCTGAAGAACAGTCTGAATTCGACGTTATCCTGGAAGCTGCTGGTGACAAAAAAATCGGTGTTATCAAAGTTGTTCGTGAAATCGTTTCTGGTCTGGGTCTGAAAGAAGCTAAAGACCTGGTTGACGGTGCTCCGAAACCGCTGCTGGAAAAAGTTGCTAAAGAAGCTGCTGACGAAGCTAAAGCTAAACTGGAAGCTGCTGGTGCTACCGTTACCGTTAAAGAAGCTGCTGCTAAATGGACCGCTGGTGCTGCTGCTTACTACGCTGCTTACAAAGTTGGTGGTAACTACAACTACCGTGCTGCTTACCGTTCTTACTCTTTCCGTCCGACCTACGCTGCTTACAAAACCTTCCCGCCGACCGAACCGAAAGCTGCTTACACCCTGGCTATCCTGACCGCTCTGCGTGCTGCTTACATCACCGTTGCTACCTCTCGTACCCTGGGTCCGGGTCCGGGTGGTATCAACATAACTAGGTTCCAGACCCTGCTCGCTCTGCACCGTGGTCCGGGTCCGGGTGACTACTCTGTTCTGTACAACCTGGCTCCGTTCTTCACCTTCAAAGGTCCGGGTCCGGGTACCAACTCTCGTCGTCGTGCTCGTTCTGTTGCTTCTCAGTCTATCGGTCCGGGTCCGGGTGCTCTGGCTCTGCTGCTGCTGGACCGTCTGAACCAGCTGGAATCTGGTCCGGGTCCGGGTGTTGGTCTGATGTGGCTGTCTTACTTCATCGCTTCTTTCCGTCTGGGCCCGGGGCCGGGCTTCTACGTATACAGCCGTGTTAAAAACCTGAACTCTTCTCGTGTTGGTCCGGGTCCGGGTGCTAAATTCGTTGCTGCTTGGACCCTGAAAGCTGCTGCTGGTCCGGGTCCGGGTAAAATGAAAGACCTGTCTCCGCGTTGGTACTTCTACTACCTGGGTGGTCCGGGTCCGGGTACCTTCAAATGCTACGGTGTTTCTCCGACCAAACTGAACGACCTGGGTCCGGGTCCGGGTGTTACCCTGTTCGTTGCTCTGTACGACTACGAAGCTCGTACCGAAGACGACCTCAGCTTCCACAAAGGTGAGAAATTCCAGATCCTGAACTCTTCTGAAGGTGACTGGTGGGAAGCTCGTTCTCTGACCACCGGTGAAACCGGTTACATCCCGTCTAACTACGTTGCTCCGGTTGACTCTATCAAAAAAATCACCCCGGGTACCAACACCTCTAACAAAAAACAGTCTTACGGTTTCCAGCCGACCAACAAAAAACGTGAACCGGAAGACCTGCCGCAGGGTAAAAAACGTGGTGGTGACGGTAAAATGAAAGACAAAAAACCGCTGCTGGAATCTGAACTGGTTATCAAAAAACGTGTTAAAAACCTGAACTCTTCTCGTAAAAAATCTACCGAAATCCAGGCTGGTAACTGCTACTTCCCGAAAAAAATCGGTTCTAAACCGTGCAATGGTGTTGAAGGCTTCAACAAAAAAAATAAACCGTGCAATGGTGTAGCTGGCTTCAACTGCCACGAGCACGAACACGAACACGAACAC
