# Supplementary material for: Immunoinformatics design of a structural proteins driven multi-epitope candidate vaccine against different SARS-CoV-2 variants based on fynomer
Source: Sci Rep. 2024 May 4;14:10297. doi: 10.1038/s41598-024-61025-2 (PMC11069592; doi:10.1038/s41598-024-61025-2)
Supplement: Supplementary file 2 — Supplementary Tables. [file 41598_2024_61025_MOESM2_ESM.docx]

**List of supplementary tables**

| **Structural Protein** | **Protein ID (NCBI)** | **Size (aa)** | **Antigenicity** | **ANTIGENpro** | **Aliphatic index** | **GRAVY** |
| --- | --- | --- | --- | --- | --- | --- |
| Surface Glycoprotein | QHD43416.1 | 1273 | 0.4646 (Antigen) | 0.717053 | 84.67 | -0.079 |
| Nucleocapsid Phosphoprotein | QHD43423.2 | 419 | 0.5059 (Antigen) | 0.939976 | 52.53 | -0.971 |
| Membrane Glycoprotein | QHD43419.1 | 222 | 0.5102 (Antigen) | 0.232547 | 120.86 | 0.446 |
| Envelope Protein | QHD43418.1 | 75 | 0.6025 (Antigen) | 0.064102 | 144.00 | 1.128 |

**Supplementary Table S1.** Details of the target proteins and their antigenicity scores for Wuhan-Hu-1 (MN908947.3).

| **Structural Protein** | **Protein ID (NCBI)** | **Size (aa)** | **Antigenicity** | **ANTIGENpro** | **Aliphatic index** | **GRAVY** |
| --- | --- | --- | --- | --- | --- | --- |
| Surface Glycoprotein | QUD52764.1 | 1271 | 0.4716 (Antigen) | 0.746593 | 84.50 | -0.085 |
| Nucleocapsid Phosphoprotein | QUD52772.1 | 419 | 0.5034 (Antigen) | 0.942455 | 52.53 | -0.943 |
| Membrane Glycoprotein | QUD52767.1 | 222 | 0.4896 (Antigen) | 0.200877 | 119.10 | 0.423 |
| Envelope Protein | QUD52766.1 | 75 | 0.6025 (Antigen) | 0.064102 | 144.00 | 1.128 |

**Supplementary Table S2.** Details of the target protein and their antigenicity score for Delta (B.1.617.2) (MZ009823.1).

| **Structural Protein** | **Protein ID (NCBI)** | **Size (aa)** | **Antigenicity** | **ANTIGENpro** | **Aliphatic index** | **GRAVY** |
| --- | --- | --- | --- | --- | --- | --- |
| Surface Glycoprotein | UFO69279.1 | 1270 | 0.4759 (Antigen) | 0.735600 | 84.95 | -0.080 |
| Nucleocapsid Phosphoprotein | UFO69287.1 | 416 | 0.5133 (Antigen) | 0.938396 | 53.85 | -0.953 |
| Membrane Glycoprotein | UFO69282.1 | 222 | 0.5259 (Antigen) | 0.182465 | 120.41 | 0.449 |
| Envelope Protein | UFO69281.1 | 75 | 0.5977 (Antigen) | 0.053355 | 149.20 | 1.197 |

**Supplementary Table S3.** Details of the target protein and their antigenicity score for Omicron (B.1.1.529) (OL672836.1).

| **Epitopes** | **Supertypes** | **HLA class I alleles** | **IC50** | **Immunogenicity** | **Antigenicity** | **Allergenicity/Toxicity** |
| --- | --- | --- | --- | --- | --- | --- |
| FVFLVLLPL  (2-10) | A2, A26, B8, B62 | HLA-A*02:50, HLA-A*68:23, HLA-B*27:20, HLA-A*02:06, HLA-B*40:13, HLA-A*02:17, HLA-A*02:01, HLA-B*15:02, HLA-A*02:02, HLA-C*12:03, HLA-A*32:07, HLA-A*68:02, HLA-A*02:03, HLA-A*02:11, HLA-A*32:15, HLA-C*03:03, HLA-B*15:17, HLA-C*14:02, HLA-B*39:01, HLA-B*35:01, HLA-A*02:16 | 4.66/11.87/12.74/14.04/16.86/21.11/25.41/27.24/31.04/33.97/36.14/43.11/54.19/63.72/65.65/82.71/99.70/101.53/120.61/165.03/186.41 | 0.04076 | 0.8601  (Antigen) | Non-Allergen/ Non-Toxin |
| VFLVLLPLV  (3-11) | A24 | HLA-A*32:07, HLA-B*27:20, HLA-A*68:23, HLA-A*02:50, HLA-C*14:02, HLA-A*02:16, HLA-C*12:03, HLA-A*02:06, HLA-B*40:13 | 12.61/12.77/17.28/25.65/37.72/49.25/49.56/71.70/83.54 | 0.00086 | 0.4651  (Antigen) | Non-Allergen/ Non-Toxin |
| VNLRTRTQL  (16-24) | B39 | HLA-B*27:20, HLA-C*03:03, HLA-A*32:07, HLA-A*68:23, HLA-A*02:50, HLA-B*15:02, HLA-B*40:13, HLA-C*14:02, HLA-C*14:02, HLA-C*12:03, HLA-A*02:17, HLA-B*14:02 | 7.98/19.61/21.28/24.30/29.66/97.10/119.93/120.12/120.12/130.68/178.92/195.00 | 0.10008 | 1.4448  (Antigen) | Non-Allergen/ Non-Toxin |
| VTWFHAIHV  (62-70) | A2 | HLA-A*68:23, HLA-A*02:11, HLA-B*27:20, HLA-A*02:06, HLA-C*12:03, HLA-A*02:16, HLA-A*32:07, HLA-C*14:02, HLA-A*02:50, HLA-A*69:01, HLA-B*40:13, HLA-A*02:01, HLA-C*15:02, HLA-A*32:15 | 2.69/10.89/27.95/37.89/40.29/40.97/57.41/112.88/117.00/119.63/133.63/161.84/171.95/182.91 | 0.38925 | 0.5426  (Antigen) | Non-Allergen/ Non-Toxin |
| HVSGTNGTK  (69-77) | A3 | HLA-A*32:07, HLA-A*68:23, HLA-C*03:03, HLA-C*12:03, HLA-B*40:13, HLA-A*68:01, HLA-A*11:01, HLA-A*30:01 | 7.56/9.13/23.96/34.69/81.83/91.51/168.77/198.59 | 0.06339 | 1.0956  (Antigen) | Non-Allergen/ Non-Toxin |
| VLPFNDGVY  (83-91) | A1, B62 | HLA-B*27:20, HLA-A*32:07, HLA-C*12:03, HLA-A*68:23, HLA-C*14:02, HLA-A*32:15, HLA-B*40:13, HLA-A*30:02, HLA-B*15:02, HLA-A*02:17 | 6.23/19.81/20.05/33.47/37.98/59.19/61.93/83.64/94.02/144.09 | 0.1815 | 0.4642  (Antigen) | Non-Allergen/ Non-Toxin |
| LPFNDGVYF  (84-92) | B7 | HLA-B*35:01, HLA-A*32:07, HLA-B*15:03, HLA-B*40:13, HLA-C*12:03, HLA-A*32:15, HLA-A*68:23, HLA-B*42:01, HLA-B*27:20, HLA-B*53:01, HLA-C*03:03 | 23.31/23.55/24.89/29.98/43.97/48.33/51.85/56.33/75.59/127.02/182.22 | 0.11767 | 0.5593  (Antigen) | Non-Allergen/ Non-Toxin |
| GVYFASTEK  (89-97) | A3 | HLA-A*68:23, HLA-C*03:03, HLA-B*27:20, HLA-A*11:01, HLA-C*12:03, HLA-A*32:07, HLA-A*03:01, HLA-C*14:02, HLA-C*15:02, HLA-A*68:01, HLA-A*30:01 | 5.18/11.47/20.20/27.30/29.05/29.99/54.05/91.54/101.02/123.16/199.51 | 0.09023 | 0.7112  (Antigen) | Non-Allergen/ Non-Toxin |
| YSKHTPINL  (204-212) | B39 | HLA-C*12:03, HLA-A*02:50, HLA-B*27:20, HLA-A*02:17, HLA-A*68:23, HLA-B*15:02, HLA-A*32:07, HLA-C*03:03, HLA-B*15:17, HLA-C*14:02, HLA-C*15:02 | 14.00/16.22/16.65/24.30/31.24/44.07/45.81/51.47/112.90/117.65/126.59 | 0.09845 | 1.0547  (Antigen) | Non-Allergen/ Non-Toxin |
| WTAGAAAYY  (258-266) | A1, A26, B58, B62 | HLA-A*68:23, HLA-B*15:17, HLA-C*03:03, HLA-A*26:02, HLA-A*29:02, HLA-A*32:15, HLA-B*40:13, HLA-C*12:03, HLA-A*30:02, HLA-B*15:02, HLA-A*26:01, HLA-A*32:07, HLA-B*35:01,HLA-B*27:20, HLA-A*68:01, HLA-C*14:02 | 1.53/4.65/6.81/16.11/25.27/40.20/52.23/52.74/58.81/71.81/78.45/82.42/83.09/89.84/141.41/195.26 | 0.15259 | 0.6306  (Antigen) | Non-Allergen/ Non-Toxin |
| GAAAYYVGY  (261-269) | A1, B58, B62 | HLA-C*03:03, HLA-C*12:03, HLA-A*68:23, HLA-A*32:07, HLA-B*27:20, HLA-A*26:02, HLA-B*40:13, HLA-B*15:17, HLA-A*32:15, HLA-B*35:01 | 14.84/16.99/17.89/33.42/35.43/47.56/79.97/91.35/160.41/172.41 | 0.09963 | 0.6604  (Antigen) | Non-Allergen/ Non-Toxin |
| AAAYYVGYL  (262-270) | B7 | HLA-C*03:03, HLA-A*68:23, HLA-A*02:50, HLA-B*27:20, HLA-B*15:02, HLA-C*12:03, HLA-A*02:02, HLA-A*32:07, HLA-B*40:13, HLA-B*15:17, HLA-A*32:15, HLA-A*26:02 | 4.54/12.81/13.09/21.25/48.33/61.54/87.50/95.73/119.10/132.34/163.77/190.24 | 0.07068 | 0.4605  (Antigen) | Non-Allergen/ Non-Toxin |
| VRFPNITNL  (327-335) | B27, B39 | HLA-B*27:20, HLA-C*06:02, HLA-C*07:01, HLA-B*40:13, HLA-C*07:02, HLA-C*12:03, HLA-A*02:50, HLA-A*32:07, HLA-A*68:23, HLA-B*15:02, HLA-C*03:03, HLA-A*32:15, HLA-C*14:02 | 1.31/5.80/14.39/18.19/25.65/35.33/35.74/42.76/53.54/75.02/75.44/89.17/119.29 | 0.1748 | 1.1141  (Antigen) | Non-Allergen/ Non-Toxin |
| FNATRFASV  (342-350) | B8 | HLA-B*27:20, HLA-A*02:50, HLA-A*68:23, HLA-C*03:03, HLA-C*12:03, HLA-B*40:13, HLA-B*08:01, HLA-A*32:07, HLA-A*68:02, HLA-A*32:15, HLA-A*02:02, HLA-A*02:06, HLA-B*54:01, HLA-A*02:03 | 3.22/4.70/17.89/19.84/25.18/32.87/49.37/49.66/52.31/76.78/89.33/114.96/153.06/194.51 | 0.14872 | 0.5609  (Antigen) | Non-Allergen/ Non-Toxin |
| GQTGKIADY  (413-421) | B27, B62 | HLA-B*27:20, HLA-A*68:23, HLA-A*32:07, HLA-C*03:03, HLA-C*12:03, HLA-B*40:13, HLA-A*32:15, HLA-A*30:02 | 7.47/9.67/10.56/15.68/36.07/38.45/76.95/157.92 | 0.00796 | 1.4019  (Antigen) | Non-Allergen/ Non-Toxin |
| SKVGGNYNY  (443-451) | B27 | HLA-A*32:07, HLA-A*68:23, HLA-B*15:03, HLA-C*12:03, HLA-B*40:13, HLA-A*32:15, HLA-B*15:02, HLA-C*03:03, HLA-B*27:20 | 14.09/15.73/21.78/23.39/25.87/38.39/46.58/51.59/59.63 | 0.06751 | 0.9111  (Antigen) | Non-Allergen/ Non-Toxin |
| YQPYRVVVL  (505-513) | A24, B8, B39, B62 | HLA-B*27:20, HLA-A*02:17, HLA-B*40:13, HLA-A*02:50, HLA-B*15:03, HLA-A*32:07, HLA-A*68:23, HLA-C*12:03, HLA-A*02:06, HLA-A*32:15, HLA-A*02:11, HLA-B*39:01, HLA-B*15:02, HLA-B*15:01, HLA-C*14:02, HLA-C*03:03 | 2.26/7.00/8.58/21.04/21.33/21.67/33.47/37.77/68.16/68.58/74.01/75.92/92.94/181.97/198.89/199.80 | 0.1409 | 0.5964  (Antigen) | Non-Allergen/ Non-Toxin |
| PYRVVVLSF  (507-515) | A24 | HLA-C*14:02, HLA-A*32:07, HLA-B*27:20, HLA-A*24:03, HLA-A*23:01, HLA-C*12:03, HLA-C*07:02, HLA-A*68:23, HLA-A*32:15, HLA-B*42:01, HLA-B*15:02, HLA-A*24:02 | 9.45/11.91/16.92/43.00/46.71/61.97/66.86/68.50/102.62/127.57/173.87/193.92 | 0.03138 | 1.0281  (Antigen) | Non-Allergen/ Non-Toxin |
| QLTPTWRVY  (628-636) | A1, B62 | HLA-B*27:20, HLA-A*32:07, HLA-C*03:03, HLA-A*68:23, HLA-C*12:03, HLA-C*14:02, HLA-B*15:02, HLA-C*07:02, HLA-B*40:13, HLA-A*32:15 | 1.89/10.69/29.48/43.12/44.17/54.77/90.20/121.66/129.99/137.48 | 0.31555 | 1.2119  (Antigen) | Non-Allergen/ Non-Toxin |
| IGAGICASY  (666-674) | B58, B62 | HLA-C*03:03, HLA-A*68:23, HLA-C*12:03, HLA-B*27:20, HLA-A*32:07, HLA-A*32:15, HLA-B*40:13, HLA-A*30:02, HLA-B*35:01 | 8.74/22.73/32.37/33.30/35.56/42.19/77.61/82.12/151.90 | 0.06201 | 0.6368  (Antigen) | Non-Allergen/ Non-Toxin |
| LGAENSVAY  (699-707) | B62 | HLA-B*35:01, HLA-B*27:20, HLA-C*03:03, HLA-C*12:03, HLA-A*32:07, HLA-A*68:23, HLA-B*40:13, HLA-B*15:01, HLA-B*15:02, HLA-B*15:03 | 8.58/20.34/20.92/45.94/51.52/56.72/103.49/122.17/148.67/160.35 | 0.00912 | 0.4173  (Antigen) | Non-Allergen/ Non-Toxin |
| IAIPTNFTI  (712-720) | A24, B58 | HLA-C*03:03, HLA-A*02:50, HLA-C*12:03, HLA-B*27:20, HLA-B*15:17, HLA-B*58:01, HLA-A*32:07, HLA-A*02:17, HLA-B*40:13, HLA-A*68:23, HLA-B*53:01, HLA-C*15:02, HLA-A*02:06, HLA-A*32:15 | 6.02/9.84/11.04/16.53/19.62/26.62/31.84/40.79/45.28/49.51/120.74/126.30/186.44/198.72 | 0.18523 | 0.7052  (Antigen) | Non-Allergen/ Non-Toxin |
| IPTNFTISV  (714-722) | B7 | HLA-A*02:50, HLA-C*12:03, HLA-A*68:23, HLA-A*32:07, HLA-B*27:20, HLA-B*42:01, HLA-B*07:02, HLA-B*40:13, HLA-B*54:01 | 13.31/18.24/20.12/23.60/54.63/113.17/116.92/132.71/192.69 | 0.17229 | 0.8820  (Antigen) | Non-Allergen/ Non-Toxin |
| FTISVTTEI  (718-726) | A2, A26, B58 | HLA-A*68:23, HLA-A*68:02, HLA-C*03:03, HLA-A*02:50, HLA-A*02:06, HLA-C*12:03, HLA-C*15:02, HLA-B*27:20, HLA-A*02:03, HLA-A*32:07, HLA-A*02:02, HLA-B*40:13, HLA-B*15:17, HLA-A*02:11, HLA-B*58:01, HLA-A*02:17, HLA-A*02:16, HLA-A*02:01, HLA-A*32:15, HLA-A*69:01, HLA-C*14:02 | 6.77/11.68/18.09/30.84/32.92/34.61/37.62/43.50/50.22/52.12/52.60/55.96/64.22/94.69/106.24/106.57/113.10/129.15/130.09/137.04/177.67 | 0.04473 | 0.8535  (Antigen) | Non-Allergen/ Non-Toxin |
| TISVTTEIL  (719-727) | B39 | HLA-A*02:50, HLA-A*32:07, HLA-B*27:20, HLA-C*03:03, HLA-A*68:23, HLA-C*12:03, HLA-B*15:02, HLA-B*40:13, HLA-A*02:17 | 7.43/14.02/22.72/31.66/55.17/75.19/84.76/90.14/192.60 | 0.22444 | 0.4795  (Antigen) | Non-Allergen/ Non-Toxin |
| NRALTGIAV  (764-772) | B27 | HLA-B*27:20, HLA-A*02:50, HLA-A*68:23, HLA-C*12:03, HLA-B*40:13, HLA-A*32:07, HLA-B*39:01, HLA-C*14:02, HLA-B*40:13, HLA-C*03:03, HLA-C*06:02 | 2.97/15.78/27.02/37.42/42.18/51.52/59.21/59.65/123.85/177.25/177.75 | 0.20642 | 0.5302  (Antigen) | Non-Allergen/ Non-Toxin |
| TLADAGFIK  (827-835) | A3 | HLA-A*32:07, HLA-C*03:03, HLA-A*68:23, HLA-B*27:20, HLA-A*11:01, HLA-A*68:01, HLA-C*12:03, HLA-B*40:13, HLA-A*03:01 | 21.13/45.14/55.55/55.91/59.47/75.94/107.94/124.43/130.55 | 0.28158 | 0.5781  (Antigen) | Non-Allergen/ Non-Toxin |
| ARDLICAQK  (846-854) | B27 | HLA-B*27:20, HLA-C*12:03, HLA-A*68:23, HLA-A*32:07, HLA-C*05:01, HLA-C*08:02, HLA-B*40:13, HLA-A*32:15 | 2.91/8.91/31.24/41.88/89.26/121.10/145.18/156.04 | 0.04023 | 0.8157  (Antigen) | Non-Allergen/ Non-Toxin |
| GLTVLPPLL  (857-865) | A2 | HLA-A*02:50, HLA-B*27:20, HLA-A*32:07, HLA-A*68:23, HLA-C*03:03, HLA-A*02:16, HLA-A*02:02, HLA-B*15:02, HLA-C*14:02, HLA-C*12:03 | 9.62/10.31/12.76/32.49/62.03/87.39/108.14/121.68/122.63/185.87 | 0.01706 | 0.6621  (Antigen) | Non-Allergen/ Non-Toxin |
| WTFGAGAAL  (886-894) | A26, B62 | HLA-A*68:23, HLA-B*15:17, HLA-C*03:03, HLA-B*40:13, HLA-B*15:02, HLA-C*12:03, HLA-A*32:07, HLA-A*32:15, HLA-A*02:50, HLA-C*14:02, HLA-A*68:02, HLA-A*02:11, HLA-B*39:01, HLA-B*27:20, HLA-A*02:17, HLA-A*26:02 | 2.58/5.76/10.63/12.21/12.59/39.19/43.55/48.89/52.02/52.79/56.96/69.71/82.30/136.92/145.76/185.06 | 0.19798 | 0.4918  (Antigen) | Non-Allergen/ Non-Toxin |
| AGAALQIPF  (890-898) | B62 | HLA-C*03:03, HLA-B*27:20, HLA-A*68:23, HLA-B*15:03, HLA-A*32:07, HLA-A*32:15, HLA-A*02:50, HLA-C*12:03, HLA-B*15:02 | 14.11/36.09/39.87/44.37/47.42/60.42/67.64/141.64/162.64 | 0.03807 | 0.4855  (Antigen) | Non-Allergen/ Non-Toxin |
| AALQIPFAM  (892-900) | B7, B58 | HLA-C*03:03, HLA-C*12:03, HLA-A*68:23, HLA-B*35:01, HLA-A*02:17, HLA-A*32:07, HLA-B*15:17, HLA-B*42:01, HLA-B*40:13 | 2.91/28.39/28.95/33.15/35.45/61.66/113.42/118.50/138.01 | 0.12066 | 0.7747  (Antigen) | Non-Allergen/ Non-Toxin |
| AEIRASANL  (1016-1024) | B44 | HLA-A*02:50, HLA-C*03:03, HLA-B*27:20, HLA-B*40:01, HLA-A*68:23, HLA-A*02:17, HLA-B*15:02, HLA-C*12:03, HLA-A*32:07, HLA-B*40:02, HLA-B*44:03 | 11.09/11.98/15.75/25.87/33.63/41.46/55.87/70.01/89.75/93.51/174.37 | 0.00689 | 0.7082  (Antigen) | Non-Allergen/ Non-Toxin |
| GVVFLHVTY  (1059-1067) | B62 | HLA-A*68:23, HLA-C*12:03, HLA-B*27:20, HLA-A*32:07, HLA-C*03:03, HLA-A*29:02, HLA-A*32:15 | 6.55/23.29/32.17/34.67/47.93/57.51/80.76 | 0.20837 | 1.4104  (Antigen) | Non-Allergen/ Non-Toxin |
| VVFLHVTYV  (1060-1068) | A2 | HLA-A*68:23, HLA-C*12:03, HLA-C*06:02, HLA-A*02:03, HLA-A*02:11, HLA-B*27:20, HLA-A*32:07, HLA-A*02:06, HLA-A*02:50, HLA-C*07:01, HLA-A*02:16, HLA-B*40:13, HLA-A*02:01, HLA-A*02:02, HLA-A*68:02, HLA-A*02:19, HLA-A*32:15, HLA-C*14:02 | 4.61/12.30/17.01/17.65/19.06/20.53/22.96/27.13/29.25/34.05/45.65/51.39/64.87/67.14/98.30/104.89/125.96/135.40 | 0.1278 | 1.5122  (Antigen) | Non-Allergen/ Non-Toxin |
| HWFVTQRNF  (1101-1109) | A24 | HLA-B*40:13, HLA-B*15:03, HLA-A*32:07, HLA-B*27:20, HLA-A*68:23, HLA-A*24:03, HLA-C*12:03, HLA-C*07:02, HLA-C*14:02, HLA-B*15:02, HLA-A*32:15, HLA-A*02:50 | 6.35/11.88/14.09/15.68/27.14/31.80/33.43/53.35/62.03/70.83/151.79/186.73 | 0.0482 | 0.7460  (Antigen) | Non-Allergen/ Non-Toxin |
| KEIDRLNEV  (1181-1189) | B44 | HLA-A*02:50, HLA-C*12:03, HLA-A*68:23, HLA-B*40:13, HLA-A*02:06, HLA-A*32:07, HLA-B*40:02, HLA-C*15:02, HLA-B*27:20, HLA-A*32:15 | 2.73/16.41/36.37/42.55/43.20/59.71/61.21/94.49/100.80/130.09 | 0.15852 | 0.5300  (Antigen) | Non-Allergen/ Non-Toxin |
| YIKWPWYIW  (1209-1217) | B58 | HLA-C*12:03, HLA-A*32:07, HLA-B*27:20, HLA-A*02:50, HLA-A*68:23, HLA-B*40:13, HLA-A*32:15, HLA-C*03:03 | 5.20/24.72/28.87/35.99/61.05/91.39/120.01/181.80 | 0.42524 | 0.9673  (Antigen) | Non-Allergen/ Non-Toxin |
| LIAIVMVTI  (1224-1232) | A2 | HLA-A*32:07, HLA-A*02:50, HLA-B*27:20, HLA-C*12:03, HLA-A*02:11, HLA-B*40:13, HLA-A*32:15, HLA-C*03:03, HLA-A*02:03, HLA-A*02:12, HLA-A*68:23, HLA-A*32:01, HLA-A*68:02 | 23.55/26.13/35.27/47.12/49.01/69.01/80.39/111.58/118.29/124.36/137.95/153.44/199.79 | 0.07904 | 1.1121  (Antigen) | Non-Allergen/ Non-Toxin |
| IAIVMVTIM  (1225-1233) | B58 | HLA-C*03:03, HLA-A*68:23, HLA-C*12:03, HLA-B*15:17, HLA-B*27:20, HLA-A*32:07, HLA-B*35:01, HLA-A*26:02, HLA-B*42:01, HLA-B*40:13, HLA-B*15:03, HLA-B*58:01, HLA-A*02:17 | 4.96/15.09/16.52/24.02/39.85/52.00/62.60/64.76/76.51/94.82/98.87/112.27/177.28 | 0.06312 | 1.1339  (Antigen) | Non-Allergen/ Non-Toxin |

**Supplementary Table S4.** CTL epitopes in spike protein.

| **Epitopes (Position)** | **Core Sequence** | **HLA class II alleles** | **IC50** | **Antigenicity** | **Allergenicity/Toxicity** |
| --- | --- | --- | --- | --- | --- |
| QDLFLPFFSNVTWFH  (52-66) | FLPFFSNVT | HLA-DRB1*15:01, HLA-DRB1*01:01, HLA-DRB1*04:04, HLA-DRB1*04:01, HLA-DRB1*04:05, HLA-DPA1*01:03/DPB1*02:01, HLA-DPA1*01/DPB1*04:01, HLA-DPA1*02:01/DPB1*01:01, HLA-DPA1*03:01/DPB1*04:02 | 34.00-56.00-73.00-89.00-104.00-66.00-69.00-93.00-115.00 | 0.4159  (Antigen) | Non-Allergen/ Non-Toxin |
| FLPFFSNVTWFHAIH  (55-69) | FFSNVTWFH | HLA-DRB1*15:01, HLA-DRB1*04:04, HLA-DRB1*04:01, HLA-DPA1*01:03/DPB1*02:01 | 102.00/152.00/160.00/123.00 | 0.4883  (Antigen) | Non-Allergen/ Non-Toxin |
| QSLLIVNNATNVVIK  (115-129) | LLIVNNATN/LIVNNATNV | HLA-DRB1*13:02, HLA-DRB1*04:04, HLA-DRB1*01:01, HLA-DRB1*04:01, HLA-DRB1*07:01, HLA-DQA1*01:02/DQB1*06:02 | 3.00/23.00/50.00/84.00/121.00/127.00 | 0.4343  (Antigen) | Non-Allergen/ Non-Toxin |
| NCTFEYVSQPFLMDL  (165-179) | YVSQPFLMD | HLA-DRB1*01:01, HLA-DRB1*07:01, HLA-DPA1*02:01/DPB1*01:01, HLA-DPA1*01:03/DPB1*02:01, HLA-DPA1*01/DPB1*04:01, HLA-DPA1*03:01/DPB1*04:02 | 73.00/167.00/62.00/62.00/104.00/120.00 | 0.5206  (Antigen) | Non-Allergen/ Non-Toxin |
| TFEYVSQPFLMDLEG  (167-181) | YVSQPFLMD | HLA-DPA1*01:03/DPB1*02:01, HLA-DPA1*02:01/DPB1*01:01, HLA-DPA1*01/DPB1*04:01, HLA-DPA1*03:01/DPB1*04:02 | 97.00/98.00/111.00/133.00 | 0.4481 (Antigen) | Non-Allergen/ Non-Toxin |
| FEYVSQPFLMDLEGK  (168-182) | FNATRFASV | HLA-DPA1*01:03/DPB1*02:01, HLA-DPA1*02:01/DPB1*01:01, HLA-DPA1*01/DPB1*04:01, HLA-DPA1*03:01/DPB1*04:02 | 102.00/102.00/122.00/135.00 | 0.8278  (Antigen) | Non-Allergen/ Non-Toxin |
| TPINLVRDLPQGFSA  (208-222) | LVRDLPQGF | HLA-DRB3*01:01, HLA-DRB1*03:01, HLA-DRB4*01:01 | 70.00/76.00/141.00 | 0.5531  (Antigen) | Non-Allergen/ Non-Toxin |
| QGFSALEPLVDLPIG  (218-232) | FSALEPLVD | HLA-DRB1*04:04, HLA-DRB1*04:05 | 37.00/61.00 | 0.6177  (Antigen) | Non-Allergen/ Non-Toxin |
| PIGINITRFQTLLAL  (230-244) | ITRFQTLLA | HLA-DRB4*01:01, HLA-DRB1*04:04, HLA-DRB1*15:01, HLA-DRB1*04:01, HLA-DRB1*07:01, HLA-DRB1*09:01, HLA-DPA1*01:03/DPB1*02:01, HLA-DPA1*03:01/DPB1*04:02, HLA-DPA1*01/DPB1*04:01, HLA-DPA1*02:01/DPB1*01:01 | 39.00/46.00/155.00/178.00/192.00/200.00/23.00/47.00/73.00/113.00 | 0.8877  (Antigen) | Non-Allergen/ Non-Toxin |
| IGINITRFQTLLALH  (231-245) | ITRFQTLLA | HLA-DRB4*01:01, HLA-DRB1*04:04, HLA-DRB1*15:01, HLA-DRB1*04:01, HLA-DRB1*07:01, HLA-DRB1*09:01, HLA-DPA1*01:03/DPB1*02:01, HLA-DPA1*03:01/DPB1*04:02, HLA-DPA1*01/DPB1*04:01, HLA-DPA1*02:01/DPB1*01:01 | 40.00/47.00/156.00/179.00/179.00/191.00/200.00/23.00/47.00/67.00/95.00 | 0.8391  (Antigen) | Non-Allergen/ Non-Toxin |
| GINITRFQTLLALHR  (232-246) | FQTLLALHR | HLA-DRB5*01:01, HLA-DRB1*04:04, HLA-DRB1*01:01, HLA-DRB1*11:01, HLA-DRB1*04:05, HLA-DRB1*04:01, HLA-DRB1*15:01, HLA-DRB4*01:01, HLA-DRB1*07:01, HLA-DRB1*09:01, HLA-DPA1*01:03/DPB1*02:01, HLA-DPA1*03:01/DPB1*04:02, HLA-DPA1*02:01/DPB1*01:01, HLA-DPA1*01/DPB1*04:01 | 2.00/10.00/11.00/41.00/66.00/76.00/93.00/105.00/129.00/141.00/65.00/96.00/120.00/153.00 | 0.5582  (Antigen) | Non-Allergen/ Non-Toxin |
| INITRFQTLLALHRS  (233-247) | FQTLLALHR | HLA-DRB5*01:01, HLA-DRB1*04:04, HLA-DRB1*01:01, HLA-DRB1*11:01, HLA-DRB1*04:05, HLA-DRB1*04:01, HLA-DRB1*15:01, HLA-DRB4*01:01, HLA-DRB1*07:01, HLA-DRB1*09:01, HLA-DPA1*01:03/DPB1*02:01, HLA-DPA1*03:01/DPB1*04:02, HLA-DPA1*02:01/DPB1*01:01, HLA-DPA1*01/DPB1*04:01 | 2.00/10.00/11.00/40.00/66.00/76.00/98.00/103.00/130.00/145.00/69.00/95.00/131.00/155.00 | 0.4118  (Antigen) | Non-Allergen/ Non-Toxin |
| RFQTLLALHRSYLTP  (237-251) | FQTLLALHR | HLA-DRB5*01:01, HLA-DRB1*01:01, HLA-DRB1*04:04, HLA-DRB1*11:01, HLA-DRB1*15:01, HLA-DRB1*07:01, HLA-DRB1*04:01 | 5.00/20.00/38.00/44.00/45.00/128.00/155.00 | 0.5470  (Antigen) | Non-Allergen/ Non-Toxin |
| FQTLLALHRSYLTPG  (238-252) | FQTLLALHR | HLA-DRB5*01:01, HLA-DRB1*15:01, HLA-DRB1*04:04, HLA-DRB1*11:01, HLA-DRB1*01:01, HLA-DRB1*07:01, HLA-DRB1*04:01, HLA-DRB1*04:05 | 5.00/46.00/38.00/44.00/20.00/134.00/158.00/167.00 | 0.5789  (Antigen) | Non-Allergen/ Non-Toxin |
| SSGWTAGAAAYYVGY  (255-269) | TAGAAAYYV | HLA-DRB1*09:01, HLA-DQA1*05:01/DQB1*03:01 | 147.00/15.00 | 0.4136  (Antigen) | Non-Allergen/ Non-Toxin |
| GWTAGAAAYYVGYLQ  (257-271) | TAGAAAYYV | HLA-DQA1*05:01/DQB1*03:01 | 21.00 | 0.5669  (Antigen) | Non-Allergen/ Non-Toxin |
| WTAGAAAYYVGYLQP  (258-272) | TAGAAAYYV | HLA-DQA1*05:01/DQB1*03:01 | 42.00 | 0.6533  (Antigen) | Non-Allergen/ Non-Toxin |
| YQTSNFRVQPTESIV  (313-327) | FRVQPTESI | HLA-DRB1*01:01, HLA-DRB1*04:01 | 58.00/185.00 | 0.7563  (Antigen) | Non-Allergen/ Non-Toxin |
| NDLCFTNVYADSFVI  (338-402) | FTNVYADSF | HLA-DRB1*07:01 | 178.00 | 0.6466  (Antigen) | Non-Allergen/ Non-Toxin |
| RFASVYAWNRKRISN  (346-360) | YAWNRKRIS | HLA-DRB1*11:01, HLA-DRB5*01:01 | 19.00/25.00 | 0.4243  (Antigen) | Non-Allergen/ Non-Toxin |
| YRVVVLSFELLHAPA  (508-522) | VLSFELLHA | HLA-DRB1*03:01, HLA-DRB4*01:01, HLA-DPA1*02:01/DPB1*01:01, HLA-DPA1*03:01/DPB1*04:02, HLA-DPA1*01:03/DPB1*02:01, HLA-DPA1*01/DPB1*04:01 | 110.00/125.00/13.00/19.00/20.00/77.00 | 0.7072 (Antigen) | Non-Allergen/ Non-Toxin |
| RVVVLSFELLHAPAT  (509-523) | VLSFELLHA | HLA-DRB1*01:01, HLA-DRB1*04:04, HLA-DRB1*03:01, HLA-DRB1*09:01, HLA-DRB1*11:01, HLA-DRB1*04:01, HLA-DRB5*01:01, HLA-DPA1*02:01/DPB1*01:01, HLA-DPA1*03:01/DPB1*04:02, HLA-DPA1*01:03/DPB1*02:01 | 5.00/16.00/111.00/128.00/147.00/194.00/199.00/34.00/47.00/58.00 | 0.7485  (Antigen) | Non-Allergen/ Non-Toxin |
| VVVLSFELLHAPATV  (510-524) | FELLHAPAT | HLA-DRB1*01:01, HLA-DRB1*04:04, HLA-DRB1*09:01, HLA-DRB1*11:01, HLA-DRB1*04:01, HLA-DPA1*02:01/DPB1*01:01, HLA-DPA1*03:01/DPB1*04:02, HLA-DPA1*01:03/DPB1*02:01 | 5.00/15.00/123.00/142.00/194.00/36.00/50.00/61.00 | 0.8083 (Antigen) | Non-Allergen/ Non-Toxin |
| VVLSFELLHAPATVC  (511-525) | FELLHAPAT | HLA-DRB1*01:01, HLA-DRB1*04:04, HLA-DRB1*09:01, HLA-DRB1*11:01 | 5.00/16.00/127.00/144.00 | 0.8618  (Antigen) | Non-Allergen/ Non-Toxin |
| VLSFELLHAPATVCG  (512-526) | FELLHAPAT | HLA-DRB1*01:01, HLA-DRB1*04:04, HLA-DRB1*09:01 | 5.00/16.00/128.00/140.00 | 0.4784 (Antigen) | Non-Allergen/ Non-Toxin |
| YECDIPIGAGICASY  (660-674) | PIGAGICAS | HLA-DQA1*05:01/DQB1*03:01 | 20.00 | 0.6775 (Antigen) | Non-Allergen/ Non-Toxin |
| ECDIPIGAGICASYQ  (661-675) | PIGAGICAS | HLA-DQA1*05:01/DQB1*03:01 | 20.00 | 0.7566  (Antigen) | Non-Allergen/ Non-Toxin |
| CDIPIGAGICASYQT  (662-676) | PIGAGICAS | HLA-DQA1*05:01/DQB1*03:01 | 21.00 | 0.7935  (Antigen) | Non-Allergen/ Non-Toxin |
| DIPIGAGICASYQTQ  (663- 677) | PIGAGICAS | HLA-DQA1*05:01/DQB1*03:01 | 21.00 | 1.1088 (Antigen) | Non-Allergen/ Non-Toxin |
| IPIGAGICASYQTQT  (664-678) | PIGAGICAS | HLA-DQA1*05:01/DQB1*03:01 | 34.00 | 0.9628  (Antigen) | Non-Allergen/ Non-Toxin |
| QSIIAYTMSLGAENS  (690-704) | YTMSLGAEN | HLA-DRB1*04:04, HLA-DRB1*09:01 | 120.00/178.00 | 0.5728 (Antigen) | Non-Allergen/ Non-Toxin |
| AIPTNFTISVTTEIL  (713-727) | FTISVTTEI | HLA-DRB1*07:01, HLA-DRB1*09:01 | 13.00/130.00 | 0.6806 (Antigen) | Non-Allergen/ Non-Toxin |
| IPTNFTISVTTEILP  (714-728) | FTISVTTEI | HLA-DRB1*07:01, HLA-DRB1*09:01 | 14.00/131.00 | 0.8294 (Antigen) | Non-Allergen/ Non-Toxin |
| PTNFTISVTTEILPV  (715-729) | FTISVTTEI | HLA-DRB1*07:01, HLA-DRB1*09:01 | 13.00/127.00 | 1.1349 (Antigen) | Non-Allergen/ Non-Toxin |
| TNFTISVTTEILPVS  (716-730) | FTISVTTEI | HLA-DRB1*07:01, HLA-DRB1*09:01 | 13.00/127.00 | 1.1691 (Antigen) | Non-Allergen/ Non-Toxin |
| NFTISVTTEILPVSM  (717-731) | FTISVTTEI | HLA-DRB1*07:01 | 38.00 | 1.2136 (Antigen) | Non-Allergen/ Non-Toxin |
| TQLNRALTGIAVEQD  (761-775) | LTGIAVEQD | HLA-DRB1*01:01, HLA-DRB1*09:01, HLA-DQA1*01:02/DQB1*06:02, HLA-DQA1*05:01/DQB1*03:01 | 64.00/ 151.00/93.00/132.00 | 0.4153 (Antigen) | Non-Allergen/ Non-Toxin |
| LNRALTGIAVEQDKN  (763-777) | LTGIAVEQD | HLA-DQA1*01:02/DQB1*06:02 | 127.00 | 0.4710 (Antigen) | Non-Allergen/ Non-Toxin |
| ALLAGTITSGWTFGA  (876-890) | LLAGTITSG | HLA-DQA1*05:01/DQB1*03:01 | 157.00 | 0.4100 (Antigen) | Non-Allergen/ Non-Toxin |
| ITSGWTFGAGAALQI  (882-896) | FGAGAALQI | HLA-DRB1*09:01, HLA-DRB1*01:01, HLA-DRB1*07:01, HLA-DQA1*05:01/DQB1*03:01 | 42.00/46.00/64.00/14.00 | 0.4483 (Antigen) | Non-Allergen/ Non-Toxin |
| GWTFGAGAALQIPFA  (885-899) | FGAGAALQI | HLA-DRB1*09:01, HLA-DRB1*01:01, HLA-DRB1*07:01, HLA-DQA1*05:01/DQB1*03:01, HLA-DQA1*01:02/DQB1*06:02 | 44.00/59.00/66.00/14.00/133.00 | 0.4665  (Antigen) | Non-Allergen/ Non-Toxin |
| WTFGAGAALQIPFAM  (886-900) | FGAGAALQI | HLA-DRB1*09:01, HLA-DRB1*01:01, HLA-DRB1*07:01, HLA-DQA1*05:01/DQB1*03:01, HLA-DQA1*01:02/DQB1*06:02 | 46.00/61.00/66.00/21.00/134.00 | 0.6670  (Antigen) | Non-Allergen/ Non-Toxin |
| TFGAGAALQIPFAMQ  (887-901) | FGAGAALQI | HLA-DRB1*09:01, HLA-DRB1*07:01, HLA-DQA1*05:01/DQB1*03:01, HLA-DQA1*01:02/DQB1*06:02 | 130.00/187.00/31.00/156.00 | 0.6932 (Antigen) | Non-Allergen/ Non-Toxin |
| FGAGAALQIPFAMQM  (888-902) | FGAGAALQI | HLA-DRB1*09:01, HLA-DRB1*07:01, HLA-DQA1*05:01/DQB1*03:01, HLA-DQA1*01:02/DQB1*06:02 | 131.00/146.00/61.00/178.00 | 0.7854 (Antigen) | Non-Allergen/ Non-Toxin |
| AALQIPFAMQMAYRF  (892-906) | FAMQMAYRF | HLA-DRB1*01:01, HLA-DRB5*01:01 | 29.00/32.00 | 0.9108 (Antigen) | Non-Allergen/ Non-Toxin |
| LQIPFAMQMAYRFNG  (894-908) | FAMQMAYRF | HLA-DRB1*01:01, HLA-DRB5*01:01 | 24.00/34.00 | 0.7205 (Antigen) | Non-Allergen/ Non-Toxin |
| QIPFAMQMAYRFNGI  (895-909) | FAMQMAYRF | HLA-DRB1*01:01, HLA-DRB5*01:01 | 22.00/32.00 | 0.9573 (Antigen) | Non-Allergen/ Non-Toxin |
| IPFAMQMAYRFNGIG  (896-910) | FAMQMAYRF | HLA-DRB1*01:01, HLA-DRB5*01:01 | 23.00/32.00 | 1.2828 (Antigen) | Non-Allergen/ Non-Toxin |
| MQMAYRFNGIGVTQN  (900-914) | YRFNGIGVT | HLA-DRB1*04:04 | 92.00 | 1.3025 (Antigen) | Non-Allergen/ Non-Toxin |
| RAAEIRASANLAATK  (1014-1028) | IRASANLAA | HLA-DRB1*01:01, HLA-DRB1*09:01, HLA-DRB1*13:02, HLA-DRB1*04:01, HLA-DRB1*04:04, HLA-DQA1*05:01/DQB1*03:01, HLA-DQA1*01:02/DQB1*06:02 | 43.00/108.00/108.00/132.00/163.00/39.00/124.00 | 0.5709 (Antigen) | Non-Allergen/ Non-Toxin |
| GVVFLHVTYVPAQEK  (1059-1073) | HVTYVPAQE | HLA-DRB1*01:01, HLA-DRB1*04:04, HLA-DRB1*01:02, HLA-DRB1*04:02, HLA-DRB1*04:02, HLA-DPA1*01:03/DPB1*02:01, DQA1*04:01-DQB1*04:02 | 90.00/168.00/102.00 | 1.1043  (Antigen) | Non-Allergen/ Non-Toxin |
| FPREGVFVSNGTHWF  (1089-1103) | FVSNGTHWF | HLA-DRB3*01:01 | 68.00 | 0.40381 (Antigen) | Non-Allergen/ Non-Toxin |

**Supplementary Table S5.** HTL epitopes in spike protein.

| **Epitopes (Position)** | **Supertypes** | **HLA class I alleles** | **IC50** | **Immunogenicity** | | **Antigenicity** | **Allergenicity/Toxicity** |
| --- | --- | --- | --- | --- | --- | --- | --- |
| QRNAPRITF  (9-17) | B27 | HLA-B*27:20, HLA-A*32:07, HLA-C*07:02, HLA-A*68:23, HLA-C*07:01, HLA-B*15:02, HLA-C*06:02, HLA-C*12:03, HLA-B*15:03, HLA-C*03:03, HLA-A*32:15, HLA-C*14:02, HLA-A*02:50 | 2.42/27.16/42.18/43.02/53.35/61.55/63.65/75.02/86.91/104.13/140.36/181.39/190.64 | | 0.21019 | 0.4654  (Antigen) | Non-Allergen/ Non-Toxin |
| SSPDDQIGY  (78-86) | A1, A26, B62 | HLA-A*32:07, HLA-A*68:23, HLA-C*12:03, HLA-B*40:13, HLA-A*02:17, HLA-A*32:15, HLA-B*27:20, HLA-C*07:01 | 7.63/11.36/15.21/19.13/53.16/83.99/108.01/166.78 | | 0.0634 | 0.5260  (Antigen) | Non-Allergen/ Non-Toxin |
| DLSPRWYFY  (103-111) | A1, A3, A26 | HLA-B*27:20, HLA-A*32:07, HLA-A*32:15, HLA-C*03:03, HLA-C*12:03, HLA-A*29:02, HLA-A*68:23, HLA-B*40:13, HLA-C*07:02 | 7.21/10.69/31.42/36.52/38.83/44.03/60.36/69.49/191.94 | | 0.25933 | 1.7645  (Antigen) | Non-Allergen/ Non-Toxin |
| LSPRWYFYY  (104-112) | A1, A3, A26, B58, A62 | HLA-A*02:17, HLA-B*27:20, HLA-C*12:03, HLA-B*40:13, HLA-A*32:07, HLA-A*68:23, HLA-B*15:17, HLA-A*32:15, HLA-A*29:02, HLA-A*01:01 | 4.00/4.84/14.49/31.61/31.69/39.69/51.25/69.22/116.88/199.77 | | 0.35734 | 1.2832  (Antigen) | Non-Allergen/ Non-Toxin |
| SPRWYFYYL  (105-113) | B7, B8 | HLA-A*02:17, HLA-B*07:02, HLA-A*68:23, HLA-B*08:01, HLA-A*32:07, HLA-B*27:20, HLA-B*15:02, HLA-B*42:01, HLA-B*40:13, HLA-A*02:50, HLA-C*12:03, HLA-A*32:15 | 20.30/20.88/22.22/23.36/23.66/31.73/50.96/62.05/67.28/79.83/92.30/130.39 | | 0.34101 | 0.7340  (Antigen) | Non-Allergen/ Non-Toxin |
| NNAAIVLQL  (153-161) | B39 | HLA-A*02:50, HLA-B*27:20, HLA-C*03:03, HLA-A*32:07, HLA-A*68:23, HLA-B*40:13, HLA-B*15:02, HLA-C*12:03, HLA-C*07:02, HLA-A*32:15 | 5.58/19.12/25.38/29.65/55.81/77.43/93.80/117.54/127.69/138.43 | | 0.14349 | 0.8662  (Antigen) | Non-Allergen/ Non-Toxin |
| LPQGTTLPK  (161-169) | B7 | HLA-C*12:03, HLA-A*32:07, HLA-A*68:23, HLA-C*03:03, HLA-B*27:20, HLA-A*02:17, HLA-C*14:02, HLA-A*11:01 | 23.67/32.96/52.20/81.02/84.81/86.42/143.09/176.31 | | 0.055 | 0.4172  (Antigen) | Non-Allergen/ Non-Toxin |
| GDAALALLL  (215-223) | B44 | HLA-B*27:20, HLA-A*02:50, HLA-A*32:07, HLA-A*68:23, HLA-C*03:03, HLA-B*15:02, HLA-B*40:13, HLA-C*12:03 | 14.97/20.14/29.65/60.22/78.81/82.45/149.59/149.69 | | 0.06226 | 0.4529  (Antigen) | Non-Allergen/ Non-Toxin |
| GMSRIGMEV  (316-324) | A2 | HLA-B*27:20, HLA-A*02:50, HLA-A*02:11, HLA-A*02:16, HLA-A*32:07, HLA-A*02:12, HLA-A*68:23, HLA-A*02:03, HLA-A*02:02, HLA-C*12:03, HLA-A*02:19, HLA-A*02:01, HLA-B*40:13, HLA-A*02:06, HLA-C*15:02, HLA-C*14:02 | 1.76/2.40/10.24/11.98/15.52/16.58/17.73/19.72/26.79/30.35/74.60/115.37/136.43/140.78/149.42/192.58 | | 0.07018 | 0.6287  (Antigen) | Non-Allergen/ Non-Toxin |
| SRIGMEVTP  (318-326) | B27 | HLA-A*32:07, HLA-B*27:20, HLA-C*12:03, HLA-C*03:03, HLA-B*40:13, HLA-A*32:15, HLA-A*68:23, HLA-C*07:02, HLA-C*07:01, HLA-A*02:50 | 14.52/15.97/30.84/34.64/43.04/52.26/55.04/106.94/132.48/192.40 | | 0.05807 | 1.6178  (Antigen) | Non-Allergen/ Non-Toxin |
| EVTPSGTWL  (323-331) | A26 | HLA-A*68:23, HLA-A*32:07, HLA-C*03:03, HLA-B*27:20, HLA-B*15:02, HLA-A*26:02, HLA-A*32:15, HLA-C*12:03, HLA-A*68:02, HLA-A*02:17, HLA-B*40:13 | 8.29/10.84/17.72/26.76/42.58/83.81/103.57/121.95/134.76/177.28/196.30 | | 0.03442 | 0.4548  (Antigen) | Non-Allergen/ Non-Toxin |
| TWLTYTGAI  (329-337) | A24 | HLA-B*27:20, HLA-C*14:02, HLA-A*32:07, HLA-C*03:03, HLA-A*68:23, HLA-A*24:03, HLA-B*40:13, HLA-A*02:50, HLA-C*12:03, HLA-A*02:17, HLA-A*32:15 | 8.11/16.85/20.04/37.03/53.30/64.20/76.90/77.12/80.57/151.23/161.15 | | 0.11986 | 0.5439  (Antigen) | Non-Allergen/ Non-Toxin |
| KTFPPTEPK  (361-369) | A3 | HLA-A*11:01, HLA-A*68:23, HLA-A*30:01, HLA-B*27:20, HLA-C*12:03, HLA-B*40:13, HLA-A*32:07, HLA-C*14:02, HLA-A*03:01, HLA-A*31:01, HLA-A*68:01, HLA-C*03:03, HLA-A*32:01 | 7.31/8.01/11.40/13.41/15.31/16.17/22.80/54.02/68.35/90.67/112.84/147.43/161.78 | | 0.1306 | 0.7571  (Antigen) | Non-Allergen/ Non-Toxin |

**Supplementary Table S6.** CTL epitopes in nucleocapsid protein.

| **Epitopes (Position)** | **Core Sequence** | **HLA class II alleles** | **IC50** | **Antigenicity** | **Allergenicity/Toxicity** |
| --- | --- | --- | --- | --- | --- |
| TASWFTALTQHGKED  (49-63) | FTALTQHGK | HLA-DRB5*01:01, HLA-DRB1*01:01 | 147.00/184.00 | 0.4491  (Antigen) | Non-Allergen/ Non-Toxin |
| ASWFTALTQHGKEDL  (50-64) | FTALTQHGK | HLA-DRB5*01:01, HLA-DRB1*01:01 | 136.00/173.00 | 0.4116  (Antigen) | Non-Allergen/ Non-Toxin |
| QIGYYRRATRRIRGG  (83-97) | YRRATRRIR/  YYRRATRRI | HLA-DRB1*11:01, HLA-DRB5*01:01, HLA-DRB1*01:01, HLA-DRB1*07:01 | 12.00/35.00/176.00/190.00 | 0.4614  (Antigen) | Non-Allergen/ Non-Toxin |
| IGYYRRATRRIRGGD  (84-98) | YRRATRRIR/  YYRRATRRI | HLA-DRB1*11:01, HLA-DRB5*01:01, HLA-DRB1*01:01, HLA-DRB1*07:01 | 13.00/37.00/178.00/192.00 | 0.6649  (Antigen) | Non-Allergen/ Non-Toxin |
| SPRWYFYYLGTGPEA  (105-119) | FYYLGTGPE | HLA-DRB1*01:01 | 93.00 | 0.8767  (Antigen) | Non-Allergen/ Non-Toxin |
| PRWYFYYLGTGPEAG  (106-120) | YYLGTGPEA | HLA-DRB1*01:01, HLA-DQA1*05:01/DQB1*03:01 | 63.00/125.00 | 0.8083  (Antigen) | Non-Allergen/ Non-Toxin |
| RWYFYYLGTGPEAGL  (107-121) | YYLGTGPEA | HLA-DRB1*01:01, HLA-DRB1*09:01, HLA-DQA1*05:01/DQB1*03:01 | 192.00/82.00 | 0.7505  (Antigen) | Non-Allergen/ Non-Toxin |
| WYFYYLGTGPEAGLP  (108-122) | YYLGTGPEA/  LGTGPEAGL | HLA-DRB1*01:01, HLA-DRB1*09:01, HLA-DQA1*05:01/DQB1*03:01 | 59.00/197.00/77.00 | 0.7188  (Antigen) | Non-Allergen/ Non-Toxin |
| GTRNPANNAAIVLQL  (147-161) | RNPANNAAI | HLA-DRB1*13:02, HLA-DQA1*01:02/DQB1*06:02, HLA-DQA1*05:01/DQB1*03:01 | 167.00/21.00/128.00 | 0.4463  (Antigen) | Non-Allergen/ Non-Toxin |
| AGNGGDAALALLLLD  (211-225) | GNGGDAAL/DAALALLLL | HLA-DQA1*05:01/DQB1*03:01, HLA-DQA1*01:02/DQB1*06:02 | 46.00/152.00 | 0.5829  (Antigen) | Non-Allergen/ Non-Toxin |
| AALALLLLDRLNQLE  (217-231) | LALLLLDRL/  LLLDRLNQL | HLA-DRB4*01:01, HLA-DRB1*03:01, HLA-DRB1*01:01, HLA-DRB1*11:01, HLA-DPA1*03:01/DPB1*04:02, HLA-DPA1*02:01/DPB1*01:01, HLA-DPA1*01:03/DPB1*02:01 | 17.00/63.00/67.00/82.00/52.00/146.00/178.00 | 0.6031  (Antigen) | Non-Allergen/ Non-Toxin |
| ALALLLLDRLNQLES  (218-232) | LLLDRLNQL/  LALLLLDRL | HLA-DRB4*01:01, HLA-DRB1*03:01, HLA-DRB1*11:01, HLA-DRB1*04:04, HLA-DRB1*01:01, HLA-DPA1*03:01/DPB1*04:02, HLA-DPA1*01:03/DPB1*02:01, HLA-DPA1*02:01/DPB1*01:01 | 28.00/63.00/81.00/85.00/156.00/62.00/172.00/177.00 | 0.5057  (Antigen) | Non-Allergen/ Non-Toxin |
| LALLLLDRLNQLESK  (219-233) | LLLDRLNQL/  LALLLLDRL | HLA-DRB4*01:01, HLA-DRB1*03:01, HLA-DRB1*11:01, HLA-DRB1*04:04, HLA-DRB1*01:01, HLA-DPA1*03:01/DPB1*04:02, HLA-DPA1*01:03/DPB1*02:01, HLA-DPA1*02:01/DPB1*01:01 | 28.00/62.00/83.00/95.00/178.00/60.00/170.00/175.00 | 0.7357  (Antigen) | Non-Allergen/ Non-Toxin |
| ATKAYNVTQAFGRRG  (264-278) | YNVTQAFGR | HLA-DRB5*01:01 | 30.00 | 0.7146  (Antigen) | Non-Allergen/ Non-Toxin |
| TKAYNVTQAFGRRGP  (265-279) | YNVTQAFGR | HLA-DRB5*01:01 | 32.00 | 0.5975  (Antigen) | Non-Allergen/ Non-Toxin |
| KAYNVTQAFGRRGPE  (266-280) | YNVTQAFGR | HLA-DRB5*01:01 | 32.00 | 0.6104  (Antigen) | Non-Allergen/ Non-Toxin |
| HWPQIAQFAPSASAF  (300-314) | IAQFAPSAS | HLA-DRB1*01:01, HLA-DRB1*09:01, HLA-DRB1*04:04, HLA-DQA1*05:01/DQB1*03:01 | 65.00/172.00/187.00/47.00 | 0.4320  (Antigen) | Non-Allergen/ Non-Toxin |
| QIAQFAPSASAFFGM  (303-317) | FAPSASAFF | HLA-DRB1*07:01, HLA-DRB1*09:01, HLA-DRB1*01:01, HLA-DRB5*01:01, HLA-DQA1*05:01/DQB1*03:01 | 32.00/56.00/75.00/95.00/46.00 | 0.4032  (Antigen) | Non-Allergen/ Non-Toxin |
| AQFAPSASAFFGMSR  (305-319) | FAPSASAFF | HLA-DRB1*07:01, HLA-DRB1*09:01, HLA-DRB5*01:01, HLA-DRB1*01:01, HLA-DQA1*05:01/DQB1*03:01 | 34.00/57.00/99.00/114.00/89.00 | 0.5266  (Antigen) | Non-Allergen/ Non-Toxin |
| QFAPSASAFFGMSRI  (306-320) | FAPSASAFF | HLA-DRB1*07:01, HLA-DRB1*09:01, HLA-DQA1*05:01/DQB1*03:01 | 102.00/170.00/171.00 | 0.4658  (Antigen) | Non-Allergen/ Non-Toxin |
| SASAFFGMSRIGMEV  (310-324) | FFGMSRIGM | HLA-DRB1*11:01, HLA-DRB1*01:01 | 71.00/105.00 | 0.6584  (Antigen) | Non-Allergen/ Non-Toxin |
| ASAFFGMSRIGMEVT  (311-325) | FFGMSRIGM | HLA-DRB1*11:01, HLA-DRB1*01:01 | 70.00/93.00 | 0.8620  (Antigen) | Non-Allergen/ Non-Toxin |
| SGTWLTYTGAIKLDD  (327-341) | LTYTGAIKL | HLA-DRB1*07:01, HLA-DRB1*01:01, HLA-DPA1*03:01/DPB1*04:02 | 15.00/99.00/174.00 | 0.6215  (Antigen) | Non-Allergen/ Non-Toxin |
| GTWLTYTGAIKLDDK  (328-342) | LTYTGAIKL | HLA-DRB1*07:01, HLA-DRB1*01:01, HLA-DPA1*03:01/DPB1*04:02 | 15.00/97.00/170.00 | 0.9934  (Antigen) | Non-Allergen/ Non-Toxin |
| TWLTYTGAIKLDDKD  (329-343) | LTYTGAIKL | HLA-DRB1*07:01, HLA-DRB1*01:01, HLA-DPA1*03:01/DPB1*04:02 | 15.00/116.00/170.00 | 1.2416  (Antigen) | Non-Allergen/ Non-Toxin |
| QKKQQTVTLLPAADL  (386-400) | TVTLLPAAD | HLA-DRB1*01:01, HLA-DRB1*04:04, HLA-DQA1*01:02/DQB1*06:02 | 24.00/26.00/154.00 | 0.7662  (Antigen) | Non-Allergen/ Non-Toxin |
| QQTVTLLPAADLDDF  (389-403) | VTLLPAADL | HLA-DRB1*01:01, HLA-DRB1*04:04 | 23.00/25.00 | 0.4614  (Antigen) | Non-Allergen/ Non-Toxin |
| QTVTLLPAADLDDFS  (390-404) | VTLLPAADL | HLA-DRB1*01:01, HLA-DRB1*04:04 | 24.00/25.00 | 0.5213  (Antigen) | Non-Allergen/ Non-Toxin |

**Supplementary Table S7.** HTL epitopes in nucleocapsid protein.

| **Epitopes (Position)** | **Supertypes** | **HLA class I alleles** | **IC50** | **Immunogenicity** | | **Antigenicity** | **Allergenicity/Toxicity** |
| --- | --- | --- | --- | --- | --- | --- | --- |
| QWNLVIGFL  (19-27) | A24 | HLA-B*27:20, HLA-A*02:50, HLA-A*02:17, HLA-A*32:07, HLA-A*68:23, HLA-A*24:03, HLA-B*15:02, HLA-C*12:03, HLA-A*32:15, HLA-B*40:13, HLA-C*03:03 | 3.20/14.90/25.38/30.83/33.17/62.30/69.53/76.95/134.04/172.15/180.97 | | 0.24922 | 1.4357  (Antigen) | Non-Allergen/ Non-Toxin |
| WNLVIGFLF  (20-28) | A24 | HLA-B*27:20, HLA-A*32:07, HLA-A*02:50, HLA-B*40:13, HLA-A*68:23, HLA-C*03:03, HLA-A*32:15, HLA-C*12:03 | 12.80/15.77/20.66/29.91/40.15/51.24/103.81/137.47 | | 0.29176 | 1.2349 (Antigen) | Non-Allergen/ Non-Toxin |
| NLVIGFLFL  (21-29) | A2 | HLA-A*02:50, HLA-B*27:20, HLA-B*15:02, HLA-A*32:07, HLA-C*03:03, HLA-A*02:06, HLA-A*02:02, HLA-A*68:23, HLA-A*02:11, HLA-C*12:03, HLA-A*02:03, HLA-C*14:02, HLA-A*02:01, HLA-A*32:15, HLA-A*02:12 | 4.37/9.51/20.95/37.24/42.71/47.04/57.14/82.17/94.26/95.76/123.58/135.71/140.96/156.40/171.27 | | 0.34956 | 1.2917 (Antigen) | Non-Allergen/ Non-Toxin |
| VIGFLFLTW  (23-31) | B58 | HLA-B*27:20, HLA-A*32:07, HLA-C*12:03, HLA-A*32:15, HLA-A*68:23, HLA-B*40:13, HLA-A*02:50, HLA-B*58:01 | 12.54/16.86/17.95/33.05/74.43/78.15/136.52/164.93 | | 0.24152 | 1.1465 (Antigen) | Non-Allergen/ Non-Toxin |
| FLFLTWICL  (26-34) | A2, B39, B62 | HLA-A*02:16, HLA-A*02:11, HLA-B*27:20, HLA-A*02:50, HLA-B*15:02, HLA-A*02:01, HLA-A*32:07, HLA-B*40:13, HLA-A*02:02, HLA-A*02:19, HLA-A*02:12, HLA-A*68:23, HLA-C*14:02, HLA-C*03:03, HLA-C*12:03, HLA-A*02:06, HLA-A*02:17, HLA-A*02:03, HLA-A*32:15, HLA-B*15:03, HLA-C*07:02 | 3.36/4.78/4.99/5.06/14.52/16.90/18.36/20.50/23.12/25.27/25.50/26.10/31.66/33.85/45.10/50.41/50.54/80.71/127.13/175.42/195.96 | | 0.35397 | 1.4835 (Antigen) | Non-Allergen/ Non-Toxin |
| LFLTWICLL  (27-35) | A24 | HLA-A*32:07, HLA-A*02:50, HLA-B*27:20, HLA-C*03:03, HLA-A*68:23, HLA-C*14:02, HLA-A*24:03, HLA-A*32:15, HLA-B*15:02, HLA-A*02:17, HLA-C*12:03, HLA-B*40:13 | 15.74/16.00/17.55/20.25/46.63/56.57/63.31/87.54/102.61/141.46/146.28/157.37 | | 0.32446 | 0.8734 (Antigen) | Non-Allergen/ Non-Toxin |
| LTWICLLQF  (29-37) | B58 | HLA-B*15:17, HLA-A*68:23, HLA-B*15:03, HLA-A*32:07, HLA-A*32:15, HLA-B*27:20, HLA-C*14:02, HLA-B*58:01, HLA-C*12:03 | 3.64/15.12/37.24/56.89/78.56/108.01/129.60/141.02/166.42 | | 0.06584 | 1.1393 (Antigen) | Non-Allergen/ Non-Toxin |
| RFLYIIKLI  (44-52) | A24 | HLA-B*27:20, HLA-A*32:07, HLA-C*03:03, HLA-A*24:03, HLA-B*40:13, HLA-A*68:23, HLA-C*14:02, HLA-C*12:03, HLA-A*32:15, HLA-A*02:50 | 5.89/7.60/28.41/31.44/32.50/33.17/66.31/81.13/107.95/167.57 | | 0.05908 | 0.4257 (Antigen) | Non-Allergen/ Non-Toxin |
| LYIIKLIFL  (46-54) | A24 | HLA-A*02:50, HLA-C*14:02, HLA-A*32:07, HLA-A*68:23, HLA-B*27:20, HLA-A*24:03, HLA-C*03:03, HLA-C*12:03, HLA-A*02:17, HLA-A*32:15 | 13.06/17.16/22.33/25.98/39.94/42.22/62.17/69.37/73.56/116.47 | | 0.1374 | 0.4865 (Antigen) | Non-Allergen/ Non-Toxin |
| KLIFLWLLW  (50-58) | B58 | HLA-B*27:20, HLA-A*32:07, HLA-B*58:01, HLA-A*02:50, HLA-A*32:01, HLA-A*32:15, HLA-C*12:03, HLA-B*57:01, HLA-B*15:17, HLA-B*40:13, HLA-A*68:23 | 2.46/10.35/11.46/19.06/22.53/32.60/43.17/56.47/82.92/94.39/98.57 | | 0.34287 | 0.4968 (Antigen) | Non-Allergen/ Non-Toxin |
| LLWPVTLAC  (56-64) | A2 | HLA-B*27:20, HLA-A*02:50, HLA-C*14:02, HLA-A*32:07, HLA-C*12:03, HLA-A*02:11, HLA-A*32:15, HLA-A*02:01, HLA-A*68:23 | 11.15/31.49/40.23/57.94/110.20/151.47/158.94/164.47/165.86 | | 0.15098 | 0.8193 (Antigen) | Non-Allergen/ Non-Toxin |
| LWPVTLACF  (57-65) | A24 | HLA-A*24:03, HLA-A*68:23, HLA-A*32:07, HLA-B*27:20, HLA-C*14:02, HLA-C*12:03, HLA-B*40:13, HLA-B*15:03, HLA-A*02:50, HLA-A*24:02 | 10.34/25.63/32.81/36.09/43.21/44.79/77.79/96.40/136.21/147.10 | | 0.06682 | 1.1590 (Antigen) | Non-Allergen/ Non-Toxin |
| WPVTLACFV  (58-66) | B7 | HLA-A*02:50, HLA-B*27:20, HLA-C*12:03, HLA-A*68:23, HLA-B*40:13, HLA-B*42:01, HLA-A*32:07, HLA-C*03:03, HLA-A*32:15 | 0.84/6.83/18.93/22.68/36.05/40.15/55.85/111.58/134.66 | | 0.10139 | 0.9626  (Antigen) | Non-Allergen/ Non-Toxin |
| ACFVLAAVY  (63-71) | B62 | HLA-C*12:03, HLA-A*68:23, HLA-B*27:20, HLA-B*40:13, HLA-B*15:03, HLA-A*32:07, HLA-A*32:15, HLA-C*03:03, HLA-A*30:02, HLA-B*15:02 | 10.38/10.51/17.00/33.18/34.76/38.46/43.17/91.32/154.33/175.07 | | 0.16271 | 1.1400  (Antigen) | Non-Allergen/ Non-Toxin |
| FVLAAVYRI  (65-73) | A2 | HLA-A*02:11, HLA-A*02:50, HLA-A*02:17, HLA-A*68:23, HLA-A*32:07, HLA-A*02:06, HLA-C*03:03, HLA-A*02:12, HLA-B*40:13, HLA-A*02:16, HLA-A*02:01, HLA-B*27:20, HLA-A*02:19, HLA-A*02:02, HLA-A*69:01, HLA-A*68:02, HLA-C*12:03, HLA-A*32:15, HLA-A*02:03, HLA-A*32:01 | 5.09/12.11/15.51/16.51/20.09/22.51/23.20/29.22/29.84/32.99/40.37/45.44/63.78/77.98/90.96/93.02/94.88/113.04/131.20/192.28 | | 0.13985 | 0.5136  (Antigen) | Non-Allergen/ Non-Toxin |
| SYFIASFRL  (94-102) | A24, B39 | HLA-A*32:07, HLA-B*40:13, HLA-A*02:50, HLA-A*24:03, HLA-B*27:20, HLA-B*15:02, HLA-A*02:17, HLA-A*68:23, HLA-C*14:02, HLA-C*12:03, HLA-C*07:02, HLA-A*32:15, HLA-A*23:01 | 6.62/8.43/19.15/20.25/23.52/23.67/25.68/26.10/36.19/40.19/41.89/48.22/186.39 | | 0.18333 | 0.4821  (Antigen) | Non-Allergen/ Non-Toxin |
| SELVIGAVI  (136-144) | B44 | HLA-A*32:07, HLA-C*03:03, HLA-A*68:23, HLA-B*40:01, HLA-C*12:03, HLA-B*40:13, HLA-B*40:02, HLA-B*27:20, HLA-A*02:50, HLA-A*32:15, HLA-B*44:03 | 12.53/28.41/38.34/47.08/56.52/59.28/70.28/72.69/88.76/104.05/187.27 | | 0.25658 | 0.6409  (Antigen) | Non-Allergen/ Non-Toxin |
| LVIGAVILR  (138-146) | A3 | HLA-A*68:23, HLA-A*68:01, HLA-A*32:07, HLA-C*03:03, HLA-C*12:03, HLA-A*31:01, HLA-A*02:50, HLA-A*32:15, HLA-A*02:17, HLA-A*11:01 | 9.11/16.34/23.49/36.52/42.67/100.10/141.00/148.33/158.00/160.06 | | 0.2601 | 1.1027  (Antigen) | Non-Allergen/ Non-Toxin |
| ITVATSRTL  (168-176) | B62 | HLA-C*03:03, HLA-A*68:23, HLA-B*15:17, HLA-A*32:07, HLA-A*02:50, HLA-C*15:02, HLA-B*15:02, HLA-C*12:03, HLA-B*27:20, HLA-A*02:17, HLA-C*14:02, HLA-B*40:13, HLA-A*02:11 | 3.66/5.49/13.14/33.81/45.41/47.25/51.43/59.59/103.39/113.67/116.57/123.00/134.37 | | 0.0012 | 0.5800  (Antigen) | Non-Allergen/ Non-Toxin |
| AGDSGFAAY  (188-196) | A1 | HLA-C*12:03, HLA-C*05:01, HLA-A*68:23, HLA-B*27:20, HLA-A*32:07, HLA-B*15:02, HLA-C*14:02, HLA-A*30:02, HLA-B*35:01, HLA-B*40:13, HLA-C*03:03, HLA-A*32:15 | 6.26/18.35/24.30/39.76/61.10/93.80/115.77/131.05/141.76/152.03/177.66/181.23 | | 0.03981 | 0.9095  (Antigen) | Non-Allergen/ Non-Toxin |

**Supplementary Table S8.** CTL epitopes in membrane protein.

| **Epitopes (Position)** | **Core Sequence** | **HLA class II alleles** | **IC50** | **Antigenicity** | **Allergenicity/Toxicity** |
| --- | --- | --- | --- | --- | --- |
| LLQFAYANRNRFLYI  (34-48) | FAYANRNRF | HLA-DRB5*01:01, HLA-DRB1*11:01, HLA-DRB1*07:01, HLA-DPA1*01:03/DPB1*02:01 | 51.00/89.00/173.00/185.00 | 0.7387 (Antigen) | Non-Allergen/ Non-Toxin |
| LAAVYRINWITGGIA  (67-81) | YRINWITGG | HLA-DRB1*01:01 | 206.00 | 1.0581  (Antigen) | Non-Allergen/ Non-Toxin |
| AAVYRINWITGGIAI  (68-82) | INWITGGIA | HLA-DRB1*01:01 | 187.00 | 0.9197  (Antigen) | Non-Allergen/ Non-Toxin |
| AVYRINWITGGIAIA  (69-83) | INWITGGIA | HLA-DRB1*01:01, HLA-DQA1*05:01/DQB1*03:01 | 157.00/64.00 | 0.9497  (Antigen) | Non-Allergen/ Non-Toxin |
| VYRINWITGGIAIAM  (70-84) | INWITGGIA | HLA-DRB1*01:01, HLA-DQA1*05:01/DQB1*03:01 | 101.00/14.00 | 0.9236  (Antigen) | Non-Allergen/ Non-Toxin |
| YRINWITGGIAIAMA  (71-85) | ITGGIAIAM | HLA-DRB1*01:01, HLA-DQA1*05:01/DQB1*03:01, HLA-DQA1*01:02/DQB1*06:02 | 96.00/11.00/60.00 | 1.1274  (Antigen) | Non-Allergen/ Non-Toxin |
| RINWITGGIAIAMAC  (72-86) | ITGGIAIAM | HLA-DRB1*01:01, HLA-DQA1*05:01/DQB1*03:01, HLA-DQA1*01:02/DQB1*06:02 | 124.00/11.00/61.00 | 1.1629  (Antigen) | Non-Allergen/ Non-Toxin |
| INWITGGIAIAMACL  (73-87) | ITGGIAIAM | HLA-DRB1*01:01, HLA-DQA1*05:01/DQB1*03:01, HLA-DQA1*01:02/DQB1*06:02 | 112.00/11.00/58.00 | 1.1352  (Antigen) | Non-Allergen/ Non-Toxin |
| NWITGGIAIAMACLV  (74-88) | ITGGIAIAM | HLA-DRB1*01:01, HLA-DQA1*05:01/DQB1*03:01, HLA-DQA1*01:02/DQB1*06:02 | 55.00/14.00/57.00 | 0.8998  (Antigen) | Non-Allergen/ Non-Toxin |
| WITGGIAIAMACLVG  (75-89) | IAIAMACLV | HLA-DRB1*01:01, HLA-DQA1*05:01/DQB1*03:01, HLA-DQA1*01:02/DQB1*06:02 | 95.00/22.00/62/00 | 0.8261  (Antigen) | Non-Allergen/ Non-Toxin |
| ITGGIAIAMACLVGL  (76-90) | IAIAMACLV | HLA-DRB1*01:01, HLA-DQA1*05:01/DQB1*03:01, HLA-DQA1*01:02/DQB1*06:02 | 55.00/30.00/123.00 | 0.9310  (Antigen) | Non-Allergen/ Non-Toxin |
| TGGIAIAMACLVGLM  (77-91) | IAIAMACLV | HLA-DRB1*01:01, HLA-DQA1*05:01/DQB1*03:01, HLA-DQA1*01:02/DQB1*06:02 | 55.00/68.00/133.00 | 0.8302  (Antigen) | Non-Allergen/ Non-Toxin |
| LVGLMWLSYFIASFR  (87-101) | LVGLMWLSY/  LMWLSYFIA | HLA-DRB5*01:01, HLA-DRB1*04:04, HLA-DRB1*01:01, HLA-DRB1*04:05, HLA-DRB1*15:01, HLA-DRB1*04:01, HLA-DPA1*01:03/DPB1*02:01, HLA-DPA1*02:01/DPB1*01:01, HLA-DPA1*01/DPB1*04:01 | 31.00/34.00/78.00/82.00/113.00/189.00/47.00/102.00/128.00 | 0.5535  (Antigen) | Non-Allergen/ Non-Toxin |
| VGLMWLSYFIASFRL  (88-102) | LSYFIASFR/  WLSYFIASF | HLA-DRB5*01:01, HLA-DRB1*04:04, HLA-DRB1*01:01, HLA-DRB1*15:01, HLA-DRB1*04:05, HLA-DRB1*07:01, HLA-DRB1*04:01, HLA-DPA1*01:03/DPB1*02:01, HLA-DPA1*02:01/DPB1*01:01, HLA-DPA1*01/DPB1*04:01 | 29.00/33.00/57.00/71.00/78.00/143.00/182.00/46.00/102.00/127.00 | 0.6658  (Antigen) | Non-Allergen/ Non-Toxin |
| SFRLFARTRSMWSFN  (99-113) | FARTRSMWS/  FRLFARTRS | HLA-DRB1*11:01, HLA-DRB1*01:01, HLA-DRB1*07:01, HLA-DRB1*04:04, HLA-DRB1*04:01 | 38.00/86.00/98.00/139.00/187.00 | 0.7955  (Antigen) | Non-Allergen/ Non-Toxin |
| FRLFARTRSMWSFNP  (100-114) | FARTRSMWS/  FRLFARTRS | HLA-DRB1*11:01, HLA-DRB1*01:01, HLA-DRB1*07:01, HLA-DRB1*04:04 | 39.00/97.00/120.00/134.00 | 0.8873  (Antigen) | Non-Allergen/ Non-Toxin |
| SELVIGAVILRGHLR  (136-150) | LVIGAVILR | HLA-DRB1*01:01, HLA-DQA1*05:01/DQB1*03:01 | 82.00/78.00 | 0.6768  (Antigen) | Non-Allergen/ Non-Toxin |
| LVIGAVILRGHLRIA  (138-152) | AVILRGHLR | HLA-DRB1*01:01, HLA-DQA1*05:01/DQB1*03:01 | 96.00/149.00 | 0.8769  (Antigen) | Non-Allergen/ Non-Toxin |
| VIGAVILRGHLRIAG  (139-153) | AVILRGHLR | HLA-DRB1*01:01, HLA-DRB1*11:01, HLA-DQA1*05:01/DQB1*03:01 | 98.00/162.00/171.00 | 0.4903  (Antigen) | Non-Allergen/ Non-Toxin |

**Supplementary Table S9.** HTL epitopes in membrane protein.

| **Epitopes (Position)** | **Supertypes** | **HLA class I alleles** | **IC50** | **Immunogenicity** | **Antigenicity** | **Allergenicity/Toxicity** |
| --- | --- | --- | --- | --- | --- | --- |
| NSVLLFLAF  (15-23) | B62 | HLA-B*27:20, HLA-A*32:07, HLA-A*32:15, HLA-B*15:02, HLA-A*68:23, HLA-C*03:03, HLA-C*12:03, HLA-B*40:13, HLA-B*15:03, HLA-B*15:17, HLA-C*14:02, HLA-B*35:01 | 10.60/46.67/65.20/69.37/75.81/81.96/84.95/93.09/97.96/106.58/132.01/135.07 | 0.11514 | 0.4134  (Antigen) | Non-Allergen/ Non-Toxin |
| SVLLFLAFV  (16-24) | A2, A26 | HLA-A*68:23, HLA-A*02:50, HLA-A*32:07, HLA-A*02:06, HLA-A*69:01, HLA-C*12:03, HLA-A*02:17, HLA-A*02:01, HLA-A*02:11, HLA-B*27:20, HLA-A*02:16, HLA-A*02:19, HLA-A*02:03, HLA-A*02:02, HLA-A*68:02 | 4.26/10.89/11.35/14.53/22.58/28.72/37.90/48.42/49.24/56.03/67.37/98.56/107.13/109.14/117.92 | 0.19022 | 0.4765  (Antigen) | Non-Allergen/ Non-Toxin |
| VLLFLAFVV  (17-25) | A2 | HLA-A*02:11, HLA-A*02:16, HLA-A*02:19, HLA-A*02:12, HLA-B*27:20, HLA-A*02:50, HLA-A*02:01, HLA-A*32:07, HLA-A*02:06, HLA-A*68:23, HLA-C*12:03, HLA-A*02:02, HLA-A*02:03, HLA-A*32:15, HLA-C*14:02 | 1.67/1.73/1.89/4.57/5.89/8.61/9.97/19.59/20.91/47.50/52.14/87.90/99.07/117.55/159.44 | 0.26315 | 0.5677  (Antigen) | Non-Allergen/ Non-Toxin |
| LLFLAFVVF  (18-26) | B8, B62 | HLA-B*15:03, HLA-B*15:01, HLA-B*27:20, HLA-A*32:07, HLA-A*32:01, HLA-C*12:03, HLA-A*32:15, HLA-B*40:13, HLA-A*68:23, HLA-C*14:02, HLA-B*15:02, HLA-C*03:03, HLA-A*02:50 | 1.97/8.51/20.86/27.48/31.91/48.44/61.41/67.13/107.58/121.79/157.11/166.57/169.12 | 0.2341 | 0.8144  (Antigen) | Non-Allergen/ Non-Toxin |
| LFLAFVVFL  (19-27) | A24 | HLA-A*32:07, HLA-C*03:03, HLA-A*02:50, HLA-A*02:17, HLA-A*24:03, HLA-B*27:20, HLA-A*68:23, HLA-C*12:03, HLA-B*40:13, HLA-B*42:01, HLA-A*02:02, HLA-A*02:11, HLA-A*02:06 | 15.74/16.96/23.29/37.55/42.61/42.80/72.06/114.34/115.59/139.87/158.85/166.08/193.88 | 0.29187 | 0.4568  (Antigen) | Non-Allergen/ Non-Toxin |
| FLAFVVFLL  (20-28) | A2, A24, B8, B39 | HLA-A*02:02, HLA-A*02:50, HLA-A*02:01, HLA-B*27:20, HLA-A*02:16, HLA-A*02:03, HLA-A*02:12, HLA-A*02:19, HLA-A*02:11, HLA-A*02:06, HLA-B*15:02, HLA-C*03:03, HLA-A*32:07, HLA-A*68:02, HLA-B*40:13, HLA-A*68:23, HLA-A*32:15, HLA-A*02:17 | 0.41/1.15/2.05/2.46/3.17/4.18/4.19/4.37/4.42/11.26/16.83/23.15/30.48/34.08/42.26/48.83/82.84/196.18 | 0.30188 | 0.5308  (Antigen) | Non-Allergen/ Non-Toxin |
| LAFVVFLLV  (21-29) | A2 | HLA-C*12:03, HLA-A*02:50, HLA-A*32:07, HLA-B*40:13, HLA-B*27:20, HLA-A*68:23, HLA-A*02:06, HLA-C*15:02, HLA-A*68:02, HLA-A*32:15 | 3.78/22.81/35.73/36.46/37.10/38.43/50.76/68.29/86.41/164.15 | 0.2141 | 0.7976  (Antigen) | Non-Allergen/ Non-Toxin |
| FVVFLLVTL  (23-31) | A2, A26, B8 | HLA-A*02:50, HLA-A*68:23, HLA-C*03:03, HLA-A*02:06, HLA-B*27:20, HLA-A*32:07, HLA-A*02:11, HLA-B*15:02, HLA-A*02:17, HLA-A*02:02, HLA-B*40:13, HLA-A*02:12, HLA-A*26:02, HLA-C*12:03, HLA-A*32:15, HLA-A*68:02, HLA-A*02:03, HLA-A*02:01 | 5.74/9.11/13.60/16.05/17.51/29.85/33.36/39.28/39.68/43.35/53.07/59.25/63.14/67.79/70.34/85.22/91.19/118.33 | 0.16748 | 0.7403  (Antigen) | Non-Allergen/ Non-Toxin |
| VFLLVTLAI  (25-33) | A24 | HLA-B*27:20, HLA-A*32:07, HLA-C*14:02, HLA-A*24:03, HLA-B*40:13, HLA-A*68:23, HLA-C*12:03, HLA-A*23:01, HLA-A*32:15 | 10.72/17.74/18.47/62.59/64.25/73.24/90.20/113.61/122.81 | 0.07548 | 0.8134  (Antigen) | Non-Allergen/ Non-Toxin |
| FLLVTLAIL  (26-34) | A2, B8, B39 | HLA-A*02:12, HLA-A*02:19, HLA-A*02:50, HLA-A*02:16, HLA-B*27:20, HLA-C*03:03, HLA-A*02:01, HLA-A*02:02, HLA-B*15:02, HLA-A*02:11, HLA-A*32:07, HLA-A*68:23, HLA-A*02:03, HLA-C*14:02, HLA-A*02:06, HLA-A*02:17, HLA-B*40:13 | 4.80/4.83/6.11/7.10/8.28/15.36/17.95/18.53/20.33/21.05/27.04/28.55/33.18/50.65/52.30/63.77/101.84 | 0.17608 | 0.9645  (Antigen) | Non-Allergen/ Non-Toxin |
| VTLAILTAL  (29-37) | A2 | HLA-A*68:23, HLA-C*03:03, HLA-C*14:02, HLA-A*32:07, HLA-A*02:11, HLA-A*02:06, HLA-B*15:17, HLA-B*27:20, HLA-C*12:03, HLA-A*32:15 | 5.46/14.11/39.13/39.17/56.01/56.82/73.40/121.75/153.18/179.99 | 0.21055 | 0.6140  (Antigen) | Non-Allergen/ Non-Toxin |
| TLAILTALR  (30-38) | A3 | HLA-B*27:20, HLA-A*68:01, HLA-A*32:07, HLA-A*02:50, HLA-A*68:23, HLA-A*31:01, HLA-C*12:03, HLA-A*32:15, HLA-C*03:03, HLA-A*33:01, HLA-B*40:13 | 8.26/19.25/20.60/29.25/35.54/78.42/106.71/114.88/114.97/127.95/171.36 | 0.1989 | 0.7223  (Antigen) | Non-Allergen/ Non-Toxin |
| LAILTALRL  (31-39) | B58 | HLA-C*03:03, HLA-B*15:17, HLA-C*12:03, HLA-A*02:50, HLA-C*15:02, HLA-A*68:23, HLA-A*02:17, HLA-B*27:20, HLA-A*32:07, HLA-B*15:02 | 19.21/20.17/32.59/36.24/42.01/50.66/53.41/54.26/61.24/119.73 | 0.12755 | 0.8872  (Antigen) | Non-Allergen/ Non-Toxin |

**Supplementary Table S10.** CTL epitopes in envelope protein.

| **Epitopes**  **(Position)** | **Core Sequence** | **HLA class II alleles** | **IC50** | **Antigenicity** | **Allergenicity/Toxicity** |
| --- | --- | --- | --- | --- | --- |
| VFLLVTLAILTALRL  (25-39) | LLVTLAILT/  LVTLAILTA/  FLLVTLAIL | HLA-DRB1*01:01, HLA-DRB1*07:01, HLA-DRB1*04:04, HLA-DRB4*01:01, HLA-DRB1*15:01, HLA-DRB5*01:01, HLA-DRB1*11:01, HLA-DRB1*04:01, HLA-DRB1*04:05, HLA-DRB1*09:01, HLA-DPA1*03:01/DPB1*04:02, HLA-DPA1*02:01/DPB1*01:01, HLA-DPA1*01:03/DPB1*02:01, HLA-DQA1*01:02/DQB1*06:02 | 6.00/25.00/55.00/56.00/58.00/74.00/95.00/98.00/122.00/144.00/44.00/82.00/114.00/189.00 | 0.7218 | Non-Allergen/ Non-Toxin |
| FLLVTLAILTALRLC  (26-40) | LAILTALRL/  LVTLAILTA/  FLLVTLAIL | HLA-DRB1*01:01, HLA-DRB1*07:01, HLA-DRB4*01:01, HLA-DRB1*15:01, HLA-DRB5*01:01, HLA-DRB1*04:04, HLA-DRB1*04:01, HLA-DRB1*11:01, HLA-DRB1*09:01, HLA-DRB1*04:05, HLA-DPA1*03:01/DPB1*04:02, HLA-DPA1*02:01/DPB1*01:01, HLA-DPA1*01:03/DPB1*02:01, HLA-DQA1*01:02/DQB1*06:02 | 6.00/27.00/59.00/69.00/76.00/77.00/105.00/108.00/147.00/161.00/49.00/102.00/124.00/193.00 | 0.6311 | Non-Allergen/ Non-Toxin |
| LVTLAILTALRLCAY  (28-42) | LAILTALRL/  ILTALRLCA | HLA-DRB1*01:01, HLA-DRB1*07:01, HLA-DRB4*01:01, HLA-DRB1*11:01, HLA-DRB1*15:01, HLA-DRB5*01:01, HLA-DRB1*04:04, HLA-DRB1*09:01, HLA-DRB1*04:01, HLA-DPA1*03:01/DPB1*04:02, HLA-DPA1*01:03/DPB1*02:01, HLA-DQA1*01:02/DQB1*06:02 | 6.00/27.00/63.00/68.00/71.00/74.00/107.00/151.00/192.00/115.00/191.00/108.00 | 0.4070 | Non-Allergen/ Non-Toxin |
| VTLAILTALRLCAYC  (29-43) | LAILTALRL/  ILTALRLCA | HLA-DRB1*01:01, HLA-DRB1*07:01, HLA-DRB4*01:01, HLA-DRB1*11:01, HLA-DRB1*15:01, HLA-DRB5*01:01, HLA-DRB1*04:04, HLA-DRB1*09:01, HLA-DPA1*03:01/DPB1*04:02, HLA-DQA1*01:02/DQB1*06:02 | 6.00/29.00/66.00/71.00/79.00/82.00/142.00/154.00/120.00/122.00 | 0.8599 | Non-Allergen/ Non-Toxin |
| TLAILTALRLCAYCC  (30-44) | LAILTALRL/  ILTALRLCA | HLA-DRB1*01:01, HLA-DRB1*07:01, HLA-DRB1*11:01, HLA-DRB4*01:01, HLA-DQA1*01:02/DQB1*06:02 | 19.00/90.00/107.00/197.00/128.00 | 0.7304 | Non-Allergen/ Non-Toxin |
| VKPSFYVYSRVKNLN  (52-66) | YVYSRVKNL/  FYVYSRVKN | HLA-DRB1*07:01, HLA-DRB1*11:01, HLA-DRB1*01:01, HLA-DRB5*01:01, HLA-DPA1*03:01/DPB1*04:02 | 42.00/70.00/123.00/175.00/137.00 | 1.2319 | Non-Allergen/ Non-Toxin |
| KPSFYVYSRVKNLNS  (53-67) | YVYSRVKNL/  FYVYSRVKN | HLA-DRB1*07:01, HLA-DRB1*11:01, HLA-DRB1*01:01, HLA-DRB1*04:04, HLA-DRB1*04:05, HLA-DRB1*04:01, HLA-DRB5*01:01, HLA-DPA1*03:01/DPB1*04:02 | 29.00/45.00/50.00/51.00/73.00/79.00/161.00/134.00 | 0.8229 | Non-Allergen/ Non-Toxin |
| PSFYVYSRVKNLNSS  (54-68) | YVYSRVKNL | HLA-DRB1*07:01, HLA-DRB1*11:01, HLA-DRB1*01:01, HLA-DRB1*04:04, HLA-DRB1*04:05, HLA-DRB1*04:01, HLA-DPA1*03:01/DPB1*04:02 | 29.00/45.00/48.00/50.00/72.00/78.00/133.00 | 0.7986 | Non-Allergen/ Non-Toxin |
| SFYVYSRVKNLNSSR  (55-69) | YVYSRVKNL/  YSRVKNLNS | HLA-DRB1*07:01, HLA-DRB1*01:01, HLA-DRB1*04:04, HLA-DRB1*11:01, HLA-DRB1*04:05, HLA-DRB1*04:01, HLA-DPA1*03:01/DPB1*04:02 | 29.00/47.00/50.00/55.00/71.00/79.00/139.00 | 0.6291 | Non-Allergen/ Non-Toxin |
| FYVYSRVKNLNSSRV  (56-70) | YSRVKNLNS/  YVYSRVKNL | HLA-DRB1*01:01, HLA-DRB1*04:04, HLA-DRB1*07:01, HLA-DRB1*11:01, HLA-DRB1*04:01, HLA-DRB1*04:05, HLA-DRB1*09:01, HLA-DRB1*13:02, HLA-DRB1*15:01 | 16.00/34.00/43.00/58.00/65.00/68.00/97.00/134.00/183.00 | 0.6103 | Non-Allergen/ Non-Toxin |
| YVYSRVKNLNSSRVP  (57-71) | YSRVKNLNS/  VKNLNSSRV | HLA-DRB1*01:01, HLA-DRB1*04:04, HLA-DRB1*07:01, HLA-DRB1*11:01, HLA-DRB1*04:01, HLA-DRB1*04:05, HLA-DRB1*09:01, HLA-DRB1*13:02, HLA-DRB1*15:01 | 17.00/35.00/44.00/62.00/66.00/69.00/100.00/137.00/197.00 | 0.4492 | Non-Allergen/ Non-Toxin |

**Supplementary Table S11.** HTL epitopes in envelope protein.

| **Protein** | **Epitope** | **IFN-γ** | **IL-4** | **IL-10** | **Antigenicity** | **Allergenicity** | **Toxicity** |
| --- | --- | --- | --- | --- | --- | --- | --- |
| Spike | TFKCYGVSPTKLNDL  (25-39) | Positive | Inducer | Inducer | 1.4626  (Antigen) | Non-Allergen | Non-Toxin |
| Spike | INITRFQTLLALHRS  (233-247) | Positive | Inducer | Inducer | 0.4118  (Antigen) | Non-Allergen | Non-Toxin |
| Spike | TRFASVYAWNRKRIS  (345-359) | Positive | Inducer | Inducer | 0.4963  (Antigen) | Non-Allergen | Non-Toxin |
| Nucleocapsid | KMKDLSPRWYFYYLG  (100-114) | Positive | Inducer | Inducer | 1.4297  (Antigen) | Non-Allergen | Non-Toxin |
| Membrane | TLACFVLAAVYRINW  (60-74) | Positive | Inducer | Inducer | 1.3132  (Antigen) | Non-Allergen | Non-Toxin |

**Supplementary Table S12.** List of candidates IFN-γ, IL-4 and IL-10 epitopes.

| **Proteins** | **Linear epitopes**  **(Position)** | **Antigenicity** | **Allergenicity** | **Toxicity** |
| --- | --- | --- | --- | --- |
| Spike | QCVNLRTRTQ  (14-23) | 1.4012 (Antigen) | Non-Allergen | Non-Toxin |
| Spike | RTRTQLPPAY  (19-28) | 1.2188 (Antigen) | Non-Allergen | Non-Toxin |
| Spike | REPEDLPQG  (208-216) | 0.7997 (Antigen) | Non-Allergen | Non-Toxin |
| Spike | HRSYLTPGDSSSGWTA  (245-260) | 0.6017 (Antigen) | Non-Allergen | Non-Toxin |
| Spike | LAPFFTFKCY  (368-377) | 1.2082 (Antigen) | Non-Allergen | Non-Toxin |
| Spike | STFKCYGVSPTKLNDL  (375-390) | 1.2492 (Antigen) | Non-Allergen | Non-Toxin |
| Spike | PGQTGNIADY  (409-418) | 1.2278 (Antigen) | Non-Allergen | Non-Toxin |
| Spike | SKVGGNYNYR  (441-450) | 1.4437 (Antigen) | Non-Allergen | Non-Toxin |
| Spike | STEIQAGNCYFP  (469-481) | 0.6932 (Antigen) | Non-Allergen | Non-Toxin |
| Spike | LQSYSFRPTY  (489-498) | 0.7731 (Antigen) | Non-Allergen | Non-Toxin |
| Spike | ITPGTNTSN  (598-606) | 1.0158 (Antigen) | Non-Allergen | Non-Toxin |
| Spike | TQTNSRRRAR  (674-683) | 0.7501 (Antigen) | Non-Allergen | Non-Toxin |
| Spike | RRRARSVASQ  (679-688) | 0.9085 (Antigen) | Non-Allergen | Non-Toxin |
| Spike | GSFCTQLKRA  (754-763) | 1.1487 (Antigen) | Non-Allergen | Non-Toxin |
| Spike | GVSVITPGTNTSNQVA  (1154-1169) | 0.4651 (Antigen) | Non-Allergen | Non-Toxin |
| Nucleocapsid | TGSNQNGERSGARSKQ  (24-39) | 0.6333 (Antigen) | Non-Allergen | Non-Toxin |
| Nucleocapsid | RGGDGKMKD  (95-103) | 0.8805 (Antigen) | Non-Allergen | Non-Toxin |
| Nucleocapsid | KSAAEASKKPRQKRTA  (249-264) | 0.4636 (Antigen) | Non-Allergen | Non-Toxin |
| Membrane | NGTITVEEL  (5-13) | 0.9235 (Antigen) | Non-Allergen | Non-Toxin |
| Membrane | PLLESELVI  (132-140) | 0.5354 (Antigen) | Non-Allergen | Non-Toxin |
| Membrane | NTDHSSSSD  (207-215) | 0.4505 (Antigen) | Non-Allergen | Non-Toxin |
| Envelope | YVYSRVKNLNSSRVPD  (57-72) | 0.5457 (Antigen) | Non-Allergen | Non-Toxin |
| Envelope | RVKNLNSSR  (61-69) | 0.8998 (Antigen) | Non-Allergen | Non-Toxin |

**Supplementary Table S13.** Details of linear/continuous B-cell (LBL) epitopes for target proteins.

| **Epitope Category** | **Population/area** | **Class I** | | |
| --- | --- | --- | --- | --- |
|  |  | **Coverage^1^** | **Average_hit^2^** | **pc90^3^** |
| **CTL Epitopes** | World | 81.14% | 2.74 | 0.53 |
|  | East Asia | 83.36% | 3.13 | 0.6 |
|  | Northeast Asia | 87.1% | 2.77 | 0.78 |
|  | South Asia | 87.05% | 2.96 | 0.77 |
|  | Southeast Asia | 80.36% | 2.09 | 0.51 |
|  | Southwest Asia | 75.37% | 2.21 | 0.41 |
|  | Europe | 83.23% | 2.99 | 0.6 |
|  | East Africa | 68.67% | 1.63 | 0.32 |
|  | West Africa | 76.64% | 1.88 | 0.43 |
|  | Central Africa | 70.28% | 1.73 | 0.34 |
|  | North Africa | 75.89% | 2.36 | 0.41 |
|  | South Africa | 88.43% | 2.87 | 0.86 |
|  | West Indies | 72.05% | 1.43 | 0.36 |
|  | North America | 80.99% | 2.53 | 0.53 |
|  | Central America | 4.14% | 0.07 | 0.1 |
|  | South America | 73.77% | 2.32 | 0.38 |
|  | Oceania | 73.37% | 2.09 | 0.38 |
|  | **Average** | 74.23 | 2.22 | 0.49 |
|  | **Standard deviation** | 18.47 | 0.73 | 0.19 |
| **Epitope Category** | **Population/area** | **Class II** | | |
|  |  | **Coverage^a^** | **Average_hit^b^** | **pc90^c^** |
| **HTL Epitopes** | World | 82.85% | 3.44 | 0.58 |
|  | East Asia | 79.46% | 3.01 | 0.49 |
|  | Northeast Asia | 59.57% | 1.89 | 0.25 |
|  | South Asia | 76.09% | 3.08 | 0.42 |
|  | Southeast Asia | 58.43% | 1.81 | 0.24 |
|  | Southwest Asia | 45.13% | 1.5 | 0.18 |
|  | Europe | 87.32% | 3.88 | 0.79 |
|  | East Africa | 68.53% | 2.11 | 0.32 |
|  | West Africa | 64.59% | 2.15 | 0.28 |
|  | Central Africa | 62.67% | 1.85 | 0.27 |
|  | North Africa | 75.76% | 2.68 | 0.41 |
|  | South Africa | 32.1% | 0.41 | 0.15 |
|  | West Indies | 69.25% | 2.68 | 0.33 |
|  | North America | 89.31% | 3.94 | 0.94 |
|  | Central America | 33.54% | 1.23 | 0.15 |
|  | South America | 49.51% | 1.75 | 0.2 |
|  | Oceania | 59.15% | 1.83 | 0.24 |
|  | **Average** | 64.31 | 2.31 | 0.37 |
|  | **Standard deviation** | 16.62 | 0.92 | 0.22 |

| **Epitope Category** | **Population/area** | **Class combined** | | |
| --- | --- | --- | --- | --- |
|  |  | **Coverage^a^** | **Average_hit^b^** | **pc90^C^** |
| **CTL and HTL Epitopes** | World | 96.77% | 6.18 | 2.05 |
|  | East Asia | 96.58% | 6.13 | 2.12 |
|  | Northeast Asia | 94.78% | 4.66 | 1.41 |
|  | South Asia | 96.9% | 6.04 | 2.01 |
|  | Southeast Asia | 91.84% | 3.89 | 1.12 |
|  | Southwest Asia | 86.48% | 3.71 | 0.74 |
|  | Europe | 97.87% | 6.88 | 2.5 |
|  | East Africa | 90.14% | 3.74 | 1.01 |
|  | West Africa | 91.73% | 4.03 | 1.15 |
|  | Central Africa | 88.91% | 3.59 | 0.9 |
|  | North Africa | 94.15% | 5.04 | 1.49 |
|  | South Africa | 92.14% | 3.28 | 1.12 |
|  | West Indies | 91.4% | 4.11 | 1.12 |
|  | North America | 97.97% | 6.48 | 2.61 |
|  | Central America | 36.29% | 1.3 | 0.16 |
|  | South America | 86.76% | 4.08 | 0.76 |
|  | Oceania | 89.12% | 3.92 | 0.92 |
|  | **Average** | 89.4 | 4.53 | 1.36 |
|  | **Standard deviation** | 13.76 | 1.39 | 0.65 |

^1^ projected population coverage

^2^ average number of epitope hits / HLA combinations recognized by the population

^3^ minimum number of epitope hits / HLA combinations recognized by 90% of the population

**Supplementary Table S14.** Population coverage selected CTL and HTL epitope in the vaccine construct.

| **Model** | **GDT-HA** | **RMSD** | **MolProbity** | **Clash score** | **Poor rotamers** | **Rama favoured** |
| --- | --- | --- | --- | --- | --- | --- |
| Initial model | 1.0000 | 0.000 | 2.761 | 56.0 | 1.6 | 94.3 |
| Model 1 | 0.9832 | 0.287 | 1.954 | 15.9 | 0.5 | 96.3 |
| Model 2 | 0.9792 | 0.310 | 1.934 | 15.8 | 0.5 | 96.5 |
| Model 3 | 0.9814 | 0.304 | 1.958 | 15.4 | 0.7 | 96.1 |
| Model 4 | 0.9837 | 0.299 | 1.973 | 15.4 | 0.7 | 95.9 |
| Model 5 | 0.9854 | 0.293 | 1.982 | 15.8 | 0.5 | 95.9 |

**Supplementary Table S15.** Quality scores of the models predicted by GalaxyRefne.

| **Structures** | **Active residues** |
| --- | --- |
| Vaccine | 140, 143, 144, 147, 148, 150, 151, 152, 153, 155, 156, 157, 159, 160, 161, 162, 163, 164, 526, 539, 540, 541, 542, 543, 544, 545, 546, 547, 557 |
| TLR2 | 63, 65, 66, 85, 87, 89, 322, 323, 326, 347, 348, 349, 350, 516, 537, 538, 539, 540, 541, 543, 544, 547, 550, 570, 571, 572, 573, 574, 575 |
| TLR4 | 35, 43, 44, 45, 46, 47, 48, 50, 51, 53, 54, 55, 65, 66, 67, 68, 70, 71, 72, 75, 76, 78, 79, 92, 96, 99, 100, 102, 124 |
| MHC I | 3, 27, 107, 108, 109, 111, 158, 159, 162, 163, 166, 167, 170, 188, 189, 190, 191, 192, 193, 194, 195, 200, 202, 204, 248, 251, 254, 274, 275 |
| MHC II | 7, 9, 10, 14, 15, 16, 24, 26, 28, 29, 30, 31, 33, 40, 41, 42, 43, 44, 45, 46, 47, 48, 49, 50, 51, 52, 54, 82, 143 |

**Supplementary Table S16.** The active residues in the candidate vaccine and receptors as predicted by CPORT.
